# Single‐Atom Ruthenium Sites on Cobalt‐Titanium Surfaces for Efficient and Selective Chloride Electrolysis

**DOI:** 10.1002/smll.72934

**Published:** 2026-02-23

**Authors:** Nael G. Yasri, Pawan Kumar, Md Golam Kibria, Edward P. L. Roberts

**Affiliations:** ^1^ Department of Chemical and Petroleum Engineering University of Calgary Calgary Alberta Canada

**Keywords:** chloride electrocatalysis, chlorine evolution reaction (CER), nanoclusters catalysts, ruthenium‐based electrocatalysts, seawater‐like solutions, single‐atom catalysts (SACs)

## Abstract

The development of efficient, stable, cost‐effective catalysts for seawater electrolysis is crucial to achieving carbon neutrality and sustainability. Isolated ruthenium single‐atom sites in Ru–O coordination environments are incorporated onto Co_2_TiO_4_/Ti supports via an electrostatic approach using Ru–ethylenediaminetetraacetic acid (EDTA) complexes, enabling atomic‐Ru dispersion through interactions between [Ru(EDTA)]^−^ and metal hydroxide precursors. The resulting Ru(SA)–Co_2_TiO_4_/Ti catalyst, with an ultra‐low Ru loading of 0.08 mg cm^−^^2^ (<0.1%), achieves an overpotential of 26.2 mV at 10 mA cm^−^^2^, a Tafel slope of 39.2 mV dec^−^^1^, and 87% selectivity toward active chlorine in seawater‐relevant sodium chloride solutions, outperforming state‐of‐the‐art CER systems while using less Ru. Compared to Ru(NC)–Co_2_TiO_4_/Ti—prepared by direct RuCl_3_ deposition without chelating agents and featuring RuO_2_ nanoclusters (NC)—the single‐site structure in Ru(SA)–Co_2_TiO_4_/Ti shows significantly improved atomic utilization and durability. Electrochemical analysis indicates that both catalysts share a rate‐determining second electron transfer step, yet Ru(SA)–Co_2_TiO_4_/Ti exhibits proton‐independent CER kinetics across pH 4.5–9. Structural characterization and X‐ray absorption spectroscopy (XANES and EXAFS) confirm the presence of oxygen‐coordinated, mixed‐valence Ru^3+^/Ru^4+^ single‐atom Ru within the orthorhombic Co_2_TiO_4_ phase, accompanied by increased Co^δ+^ and oxygen vacancies, promoting Cl^−^ adsorption and facilitating Cl_2_ formation via a positive‐valent chlorine intermediate.

## Graphical abstract

This study presents an advanced electrocatalyst featuring atomically dispersed Ru single‐atom sites anchored on a cobalt–titanium oxide support. The use of a Ru–EDTA precursor enables uniform dispersion and prevents agglomeration during thermal treatment. The catalyst achieves high chlorine selectivity and operational stability in seawater‐like media, offering a scalable solution for sustainable chlorine generation and electrochemical water treatment.

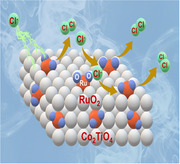

## 1 Introduction

Harnessing natural resources to convert energy into clean fuels—using seawater as a feedstock— is crucial for achieving carbon neutrality and sustainable development. Seawater is increasingly utilized in sustainable electrochemical processes such as direct seawater electrolysis for hydrogen production, electro‐desalination for clean water, and electrolytic mineralization to capture greenhouse gases [1]. However, developing active and stable anode catalysts for oxygen and chlorine oxidation remains challenging [2].

Anode stability during seawater electrolysis is compromised by the formation of highly corrosive chlorine‐based species, as well as highly oxidizing oxygenated intermediates generated under anodic conditions [3, 4, 5]. These reactive species contribute to oxidative degradation of electrode materials, leading to passivation, structural damage, and reduced catalytic performance [6]. The chlorine evolution reaction (CER) occurs in parallel with the oxygen evolution reaction (OER) at the anode. While CER is thermodynamically less favorable (E° = 1.36 V_RHE_) than OER (1.23 V_RHE_), its two‐electron mechanism renders it kinetically more accessible [7]. Moreover, the prominence of each pathway is influenced by factors such as applied potential, pH, chloride ion concentration, catalyst composition, interfacial adsorption behavior, and local mass transport conditions [8]. In industrial Cl_2_ production, high selectivity and catalyst stability are essential. While highly concentrated chloride electrolytes used in the chlor‐alkali process, operated under optimized anode conditions, can achieve high current densities (>1 A cm^−^^2^) and near‐complete Cl_2_ selectivity [9], seawater systems suffer from reduced ionic strength and parasitic OER, often lowering Cl_2_ selectivity to ∼85–90% and limiting current densities to <0.2 A cm^−^^2^ [4, 9, 10].

Various CER catalysts have been explored, including noble metal‐based, earth‐abundant transition‐metal‐based, and carbon‐based materials. Among them, mixed metal oxides (MMOs)—particularly those containing Ir or Ru—are among the most promising due to their low CER overpotentials and excellent durability [11, 12]. Alloying them with earth‐abundant metals improves stability while reducing cost [12]. However, because conventional MMO catalysts operate through the OCl‐mediated pathways—where CER proceeds through surface O–Cl intermediates that bind to the same oxygen adsorption sites used by OER intermediates (OH and O)—their chlorine selectivity is intrinsically constrained by CER–OER scaling, as detailed in several comprehensive mechanistic analyses [13, 14, 15]. These limitations highlight the need for alternative active‐site motifs that do not rely on O–Cl intermediates and instead activate chloride through fundamentally different coordination environments, as emphasized in recent reviews proposing next‐generation CER catalyst design strategies beyond traditional MMO systems [16, 17].

Recent advances in single‐atom catalysts (SACs) demonstrate that isolated metal centers enable unique Cl^−^ adsorption behavior and access alternative chloride‐activation pathways beyond those of OCl‐mediated MMO catalysts [16, 17]. Recent studies further show that high CER selectivity with suppressed OER arises from a Cl‐mediated reaction pathway that decouples chlorine evolution from oxygen adsorption, thereby breaking the intrinsic CER–OER scaling relations characteristic of MMOs and highlighting the critical role of SACs in suppressing parasitic OER [16, 17].

Beyond mechanistic considerations, reducing the amount of precious metals (i.e., Ru and Ir, <30 at%) without compromising catalytic activity and stability remains an important goal [10, 12]. However, even at reduced loadings, conventional RuO_2_ nanoparticles are prone to aggregate under anodic conditions, leading to poor atomic utilization and compromising catalytic performance [13, 14, 15, 16, 17]. Achieving further reductions in Ru usage—ideally to isolated single‐atom levels—while maintaining high activity is therefore a critical challenge in catalyst design [10, 12, 18].

Diluting precious metals to atomic dispersion levels (<0.2 at%) can dramatically improve catalytic activity due to enhanced surface energy, coordination effects, and maximized metal utilization [18]. For example, Choi et al. demonstrated that doping only 3–4 at% Ru into Ru_0_._09_Co_2_._91_O_4_ nanoparticles significantly enhanced CER performance via interfacial charge modulation and the generation of additional active sites [10]. Despite these advantages, synthesizing Ru‐based SACs on oxide supports remains challenging. Metal precursors often suffer from limited interfacial anchoring, and subsequent thermal treatment can promote migration and agglomeration into clusters, reducing atomic efficiency and selectivity [19, 20]. Although various strategies—including co‐precipitation, atomic layer deposition, galvanic replacement, and wet impregnation—have been explored to stabilize isolated Ru sites, persistent challenges remain, such as aggregation, dispersion control, and thermal sensitivity [21, 22]. Therefore, new approaches are required to prevent clustering and enable stable implantation of Ru single atoms into robust oxide lattices.

Traditional SAC preparation methods that rely on defect or vacancy trapping through impregnation often suffer from suppressed activity and side reactions arising from heterogeneous coordination environments [20]. Similarly, SAC synthesis via thermal annealing of metal salt‐impregnated support is frequently plagued by agglomeration due to the high mobility of energetically single‐atom species. In contrast, thermal fusion of ligand‐trapped metal complexes—such as porphyrins, phthalocyanines, and metal‐organic frameworks supported on graphene via π‐π interactions—has been widely used to generate atomically dispersed metal sites on carbon supports [23]. However, unlike graphitic support, metal oxide substrates lack π–π interactions, rendering the stabilization and implantation of single atoms particularly challenging; metal complexes therefore tend to aggregate before successful incorporation into the inorganic lattice [20, 24]. For effective implantation into an oxide host, metal‐ligand complexes must be sufficiently stable to prevent premature agglomeration at low temperatures, yet labile enough to implant metal centers and integrate into the host lattice at elevated temperatures.

In this context, ethylenediaminetetraacetic acid (EDTA) is identified in this work as a suitable metal‐ligand precursor, compared with porphyrins or phthalocyanines, for oxide‐supported SACs. Metal–EDTA complexes exhibit relatively mild decomposition temperature (<300 °C) [25] and can form stable, well‐defined charged metal complexes, such as the negative [Ru(EDTA)]^−^ complex. This charged nature enables electrostatic attachment to oxide‐support—rather than *π–π* interactions—thereby anchoring metal centers, suppressing migration, and minimizing agglomeration during thermal processing [26].

Building on this approach, we developed Ru single atoms implanted on a metal‐based electrode (Ru(SA)–Co_2_TiO_4_/Ti) by thermally annealing of Ru(III)‐EDTA complex anchored onto Co‐Ti hydroxide supports. This route enabled uniform implantation of Ru as isolated single‐atom sites within the Co_2_TiO_4_ spinel lattice, yielding a robust and conductive oxide matrix that promotes durability and stabilizes CER‐active centers. In this architecture, atomically dispersed Ru sites serve as the primary CER‐active centers, while the cobalt sublattice facilitates charge transport and redox mediation. Structural and spectroscopic analyses (XPS, XAFS) confirm the presence of isolated Ru sites stabilized within the oxide lattice, while electrochemical measurements further support their distinct single‐site catalytic behavior. The resulting catalyst, with an ultra‐low Ru loading of 0.08 mg cm^−^^2^ (<0.1%), delivers a low CER overpotential of 26.2 mV at 10 mA cm^−^^2^, a Tafel slope of 39.2 mV dec^−^^1^, and 87% selectivity toward active chlorine formation, while maintaining stable operation at 20 mA cm^−^^2^ for 72 h. Although complete selectivity is not achieved, this performance is consistent with the intrinsic limitations of seawater systems, where parasitic OER and reduced ionic strength constrain selectivity to ∼85%–90% [4, 9, 10]. Importantly, our catalyst emphasizes stability, atomic efficiency, and mechanistic clarity, exhibiting proton‐ and pH‐independent behavior, while overcoming the common inherent drawbacks of nanoparticulate metal dispersion.

## 2 Results and Discussion

**FIGURE 1:**
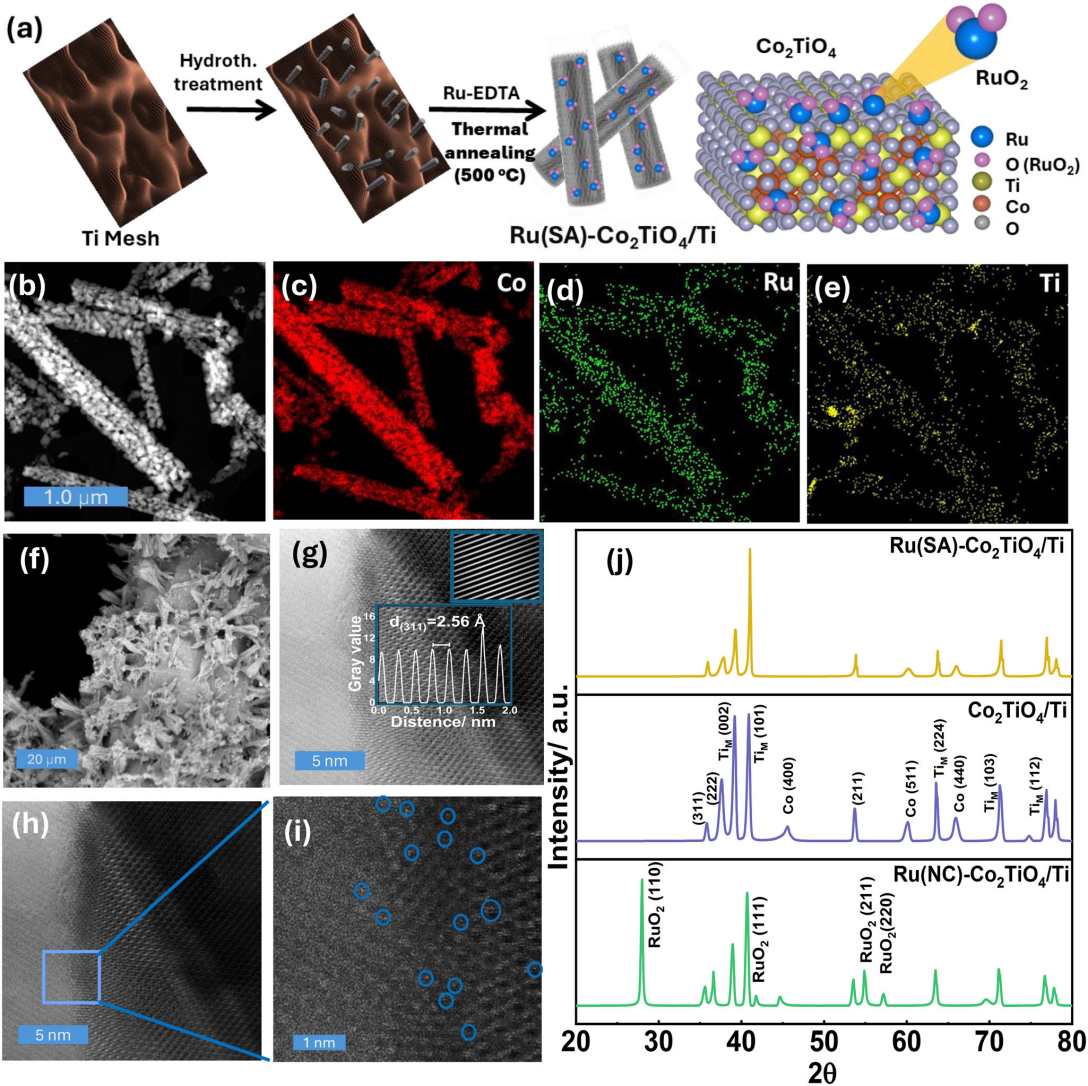
(a) Schematic illustration of the Ru(SA)–Co_2_TiO_4_/Ti catalyst synthesis protocol, (b) SEM images and the corresponding elemental distribution mapping for (c) Co, (d) Ru, and (e) Ti, of the Ru(SA)–Co_2_TiO_4_/Ti catalyst. (f) SEM image of the surface morphology of Ru(SA)–Co_2_TiO_4_/Ti catalyst, (g) HRTEM image of the prepared Ru(SA)–Co_2_TiO_4_/Ti, inset shows measured interplanar distance d(311) = 2.56 Å, along with the number of cycles and line profiles used for d‐spacing determination. (h) AC‐HAADF‐STEM images of Ru(SA)–Co_2_TiO_4_/Ti (scale bar: 5 nm), (i) magnified image of panel ‘h’ (scale bar 1 nm) highlighting atomically dispersed high‐Z contrast features consistent with isolated Ru single‐atom sites anchored within the Co_2_TiO_4_ lattice. (j) XRD patterns of (from bottom to top) Ru(NC)–Co_2_TiO_4_/Ti, Co_2_TiO_4_/Ti, and Ru(SA)–Co_2_TiO_4_/Ti; reference crystal planes are shown in Figure S5.

The synthesis of single‐atom catalysts (SACs) often utilizes metal‐entrapped organic templates, where thermal decomposition facilitates the formation of isolated metal centers with metal–oxygen coordination environments [18]. In this work, EDTA, with its multiple coordinating sites (e.g., carboxyl and amine groups), exhibits strong coordination with Ru (log K_f_ = 19.8), forming a negatively charged Ru–EDTA complex that enables electrostatic anchoring onto hydroxylated oxide surfaces [27]. This strong coordination effectively stabilizes Ru in the single‐atom state and prevents aggregation during thermal treatments [28]. Ru‐EDTA was prepared in crystal form (Sections S1.1 and S2.1) and incorporated into hydroxy Co‐Ti precursors through a three‐step process: (1) hydrothermal synthesis of hydroxy Co‐Ti structures on a Ti mesh support, where pre‐treatment with CoCl_2_ under reductive conditions (urea/ethanol) promoted hydroxide growth [29], providing abundant surface hydroxyl sites for stable precursor attachment [30]; (2) loading Ru‐EDTA onto the hydroxy Co‐Ti surface; and (3) annealing in air at 500°C (Figure 1a; Section S1.0). The resulting Ru(SA)–Co_2_TiO_4_/Ti catalyst was compared to Ru(NC)–Co_2_TiO_4_/Ti (synthesized with RuCl_3_ instead of Ru‐EDTA) and Co_2_TiO_4_/Ti (without Ru), highlighting the critical roles of EDTA complexed Ru in achieving atomic dispersion and improved catalytic performance and stability for the CER.

Raman spectroscopy confirms well‐defined Ru–EDTA coordination in the crystalline precursor, whereas these Ru–N, Ru–O, and carboxylate‐related vibrational features are completely absent after annealing, demonstrating full removal of the EDTA chelating ligand in the as‐prepared Ru(SA)–Co_2_TiO_4_/Ti catalyst (Figure S1 and Table S1). Scanning electron microscopy (SEM) images of Ru(SA)–Co_2_TiO_4_/Ti, Ru(NC)–Co_2_TiO_4_/Ti, and Co_2_TiO_4_/Ti catalysts reveal similar rough surface morphologies with nanorod‐like structures aligned on the Ti mesh (Figure 1f, Figure S2a–c). Energy‐dispersive X‐ray (EDX) mapping confirms uniform Ru distribution in Ru(SA)–Co_2_TiO_4_/Ti (Figure 1b–e). In contrast, Ru(NC)–Co_2_TiO_4_/Ti shows Ru aggregated in distinct regions, separate from Co and Ti (Figure S3). Inductively coupled plasma (ICP) analysis reveals higher Ru loading (0.19 mg cm^−^^2^) in Ru(NC)–Co_2_TiO_4_/Ti compared to 0.08 mg cm^−^^2^ for Ru(SA)–Co_2_TiO_4_/Ti (Section S1.3).

High‐angle annular dark‐field scanning transmission electron microscopy (HAADF‐STEM) and aberration‐corrected AC‐HAADF‐STEM reveal brighter dots with higher Z‐contrast across the nanoparticle surface, consistent with Ru atoms individually anchored within lattice fringes of Ru(SA)–Co_2_TiO_4_/Ti catalyst (Figure 1h, i and Figure S4). However, the low resolution of Ru single‐atom sites is attributed to the limitations of direct visualization on transition metal oxides, often affected by weak Z‐contrast and beam‐induced migration—an issue well documented in the single‐atom catalyst literature [31, 32].

XRD spectra of Ru(SA)–Co_2_TiO_4_/Ti show no additional diffraction peaks compared to Co_2_TiO_4_/Ti, which is consistent with aberration‐corrected HAADF‐STEM observations and supports atomic‐scale Ru dispersion (Figure 1j) [33]. In contrast, RuO_2_‐related peaks, particularly the (110) reflection at ∼28°, (111) at 41.7°, (211) at 54.8°, and (220) at 57.2°, are clearly observed in Ru(NC)–Co_2_TiO_4_/Ti, confirming the presence of aggregated RuO_2_ domains (see Section S2.3 and Figure S5). The diffraction peak in Co_2_TiO_4_/Ti at ∼45.6°, attributed to the (400) planes of Co_2_TiO_4_ and Co_3_O_4_ (see Figure S5), is suppressed in Ru(SA)–Co_2_TiO_4_/Ti, likely due to distortion of the (400) planes caused by EDTA‐mediated Ru dispersion. Additionally, the dominant RuO_2_ (101) reflection near 35° is not clearly resolved in Ru(NC)–Co_2_TiO_4_/Ti, likely due to overlap with the Co_2_TiO_4_ (311) plane (∼35.22°), the Ti_M_ (100) plane (∼35.26°), and the low crystallinity of Ru nanoclusters. Notably, distinct diffraction peaks at 28.0°, 41.7°, 54.8°, and 57.2° corresponding to the (110), (111), (211), and (220) planes of tetragonal RuO_2_ (mp‐825) (see Figure S5)—are clearly visible in the XRD pattern of Ru(NC)–Co_2_TiO_4_/Ti. These peaks are absent in both Co_2_TiO_4_/Ti and Ru(SA)–Co_2_TiO_4_/Ti, confirming the formation of crystalline RuO_2_ nanoclusters in the RuCl_3_‐modified catalyst. Moreover, high‐resolution TEM (HRTEM) analysis of Ru(SA)–Co_2_TiO_4_/Ti shows interplanar spacings of 2.56 Å, matching the Co_2_TiO_4_ (311) plane (Figure 1g and Figure S5) [34]. These results demonstrate that the hydrothermal/annealing synthesis conditions promote the formation of well‐crystallized Co_2_TiO_4_ spinel structures, while Ru‐EDTA facilitates Ru dispersion and lattice incorporation, suppressing aggregation and yielding atomically dispersed Ru single‐atom sites.

**FIGURE 2:**
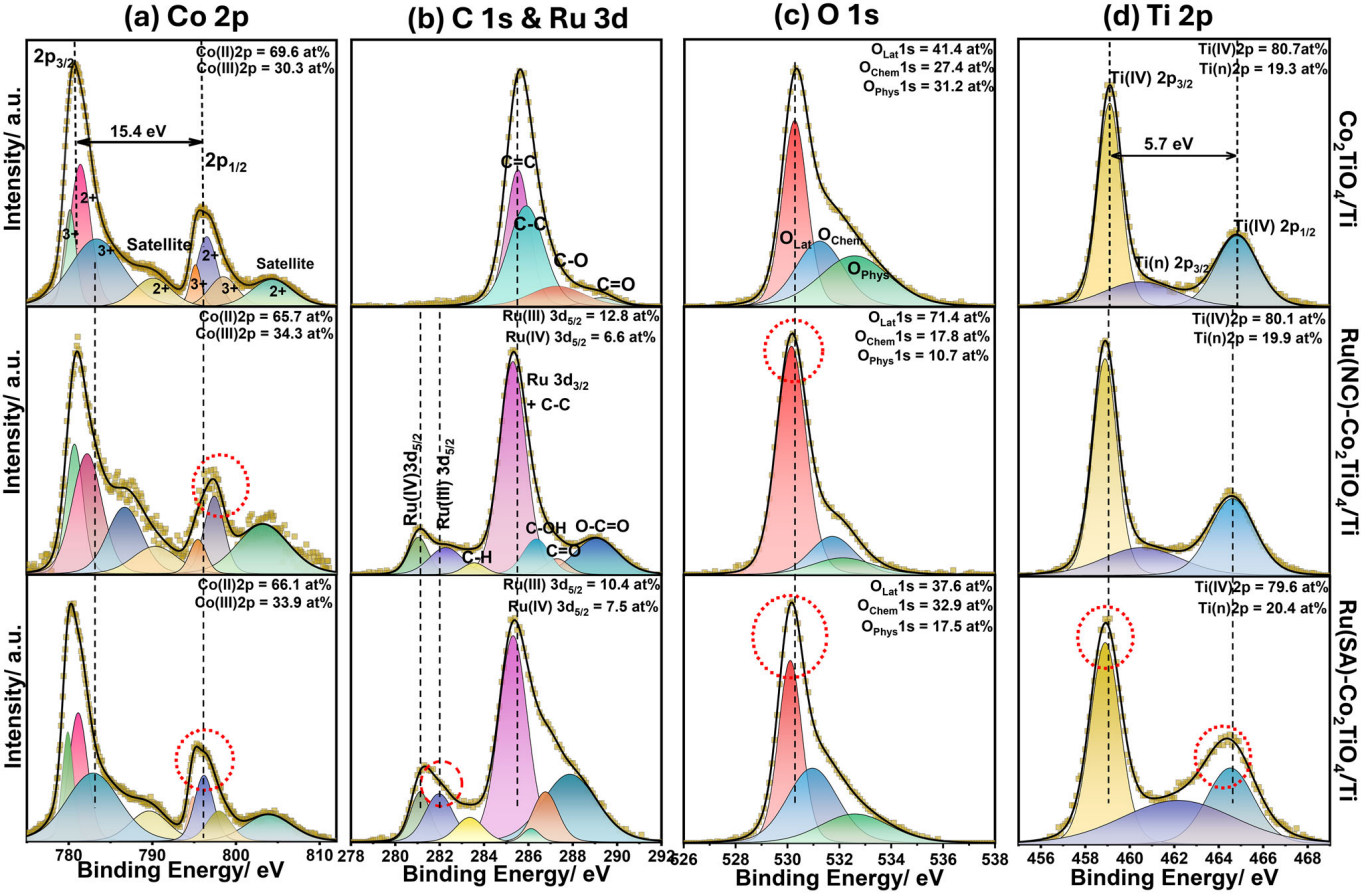
HR‐XPS in (a) Co2p, (b) C1s and Ru3d, (c) O1s, and (d) Ti 2p region aligned of lower to upper panel: Ru(SA)–Co_2_TiO_4_/Ti, Ru(NC)–Co_2_TiO_4_/Ti, and Co_2_TiO_4_/Ti catalysts.

X‐ray photoelectron spectroscopy (XPS) was used to probe the surface composition and electronic states of Ru, Co, and Ti in the prepared catalysts. XPS survey spectra (Figure S6) confirm the presence of all constituent elements, while high‐resolution spectra of Co 2p, Ru 3d, O 1s, and Ti 2p (Figure 2a–d) reveal oxidation states and electronic interactions. Spectral deconvolution details are provided in the Section S2.4.

XPS survey scans (Table S2) show ∼5.1 at% Co and ∼3.8 at% Ti on the surface of the Co_2_TiO_4_/Ti catalyst. High‐resolution Ti 2p analysis indicates that Ti is predominantly present as Ti(IV), accounting for ∼80.7% of the Ti 2p spectral area (Figure 2d), while cobalt is present in mixed Co^2^^+^/Co^3^^+^ oxidation states (Figure 2a), consistent with the chemistry of spinel cobalt titanates. Together with XRD phase identification and high‐resolution TEM (HRTEM) lattice imaging (Figure 1), these results confirm the formation of spinel Co_2_TiO_4_ on the Ti mesh substrate.

The use of urea during hydrothermal synthesis, together with Ru–EDTA introduction in Ru(SA)–Co_2_TiO_4_/Ti, leads to nitrogen incorporation, as evidenced by a weak N1s signal at 401 eV in the survey spectra (Figure S6) and metal‐N coordination component at ∼400.4 eV in high‐resolution N 1s spectra (Figure S7). These features are attributed to nitrogen species coordinated to metal centers (Ru, Co, or Ti) [28, 35]. In addition, Ru(SA)–Co_2_TiO_4_/Ti exhibits a higher fraction of surface‐chemisorbed oxygen (O_Chem_ ∼ 32.9%) than Ru(NC)–Co_2_TiO_4_/Ti (17.8%) and Co_2_TiO_4_/Ti (27.4%) (Figure 2c), which is often associated with increased oxygen mobility and surface redox dynamics. Concurrently, negative shifts in lattice oxygen peaks (O_latt_) following Ru introduction suggest the formation of oxygen vacancies that facilitate electron transfer from the active metal site [36].

High‐resolution Co 2p spectra (Figure 2a) reveal higher Co^3^^+^/Co^2^^+^ ratios for Ru(SA)–Co_2_TiO_4_/Ti and Ru(NC)–Co_2_TiO_4_/Ti (0.51 and 0.52, respectively) relative to Co_2_TiO_4_/Ti (0.43), consistent with enhanced oxidation activity [37, 38]. Furthermore, binding‐energy shifts indicate a higher effective oxidation state of Co (denoted as Co^δ+^) [39, 40] in Ru(NC)–Co_2_TiO_4_/Ti and a relatively lower effective oxidation state of Co (Co^δ−^) in Ru(SA)–Co_2_TiO_4_/Ti compared to Co_2_TiO_4_/Ti, reflecting distinct electronic interactions induced by different Ru incorporation pathways [39, 40].

Ru(SA)–Co_2_TiO_4_/Ti also shows slight negative shifts in Ru 3d and Ti 2p binding energies (Figure 2b, d), suggesting increased electron density at isolated Ru sites and the presence of undercharged Ti species (e.g., Ti^3^^+^) associated with oxygen‐vacancy formation [41, 42]. Together, these coordinated changes in metal oxidation states, oxygen chemistry, and electronic structure indicate that EDTA‐assisted Ru single‐atom incorporation electronically tailors the Co_2_TiO_4_ surface.

X‐ray absorption near‐edge structure (XANES) and extended X‐ray absorption fine structure (EXAFS) were employed to investigate the electronic structure and coordination environments of Co and Ru in Ru(SA)–Co_2_TiO_4_/Ti and Ru(NC)–Co_2_TiO_4_/Ti catalysts. Full experimental details of the test methods and fitting parameters are provided in the Sections S1.4, S2.5, and S2.6.

**FIGURE 3:**
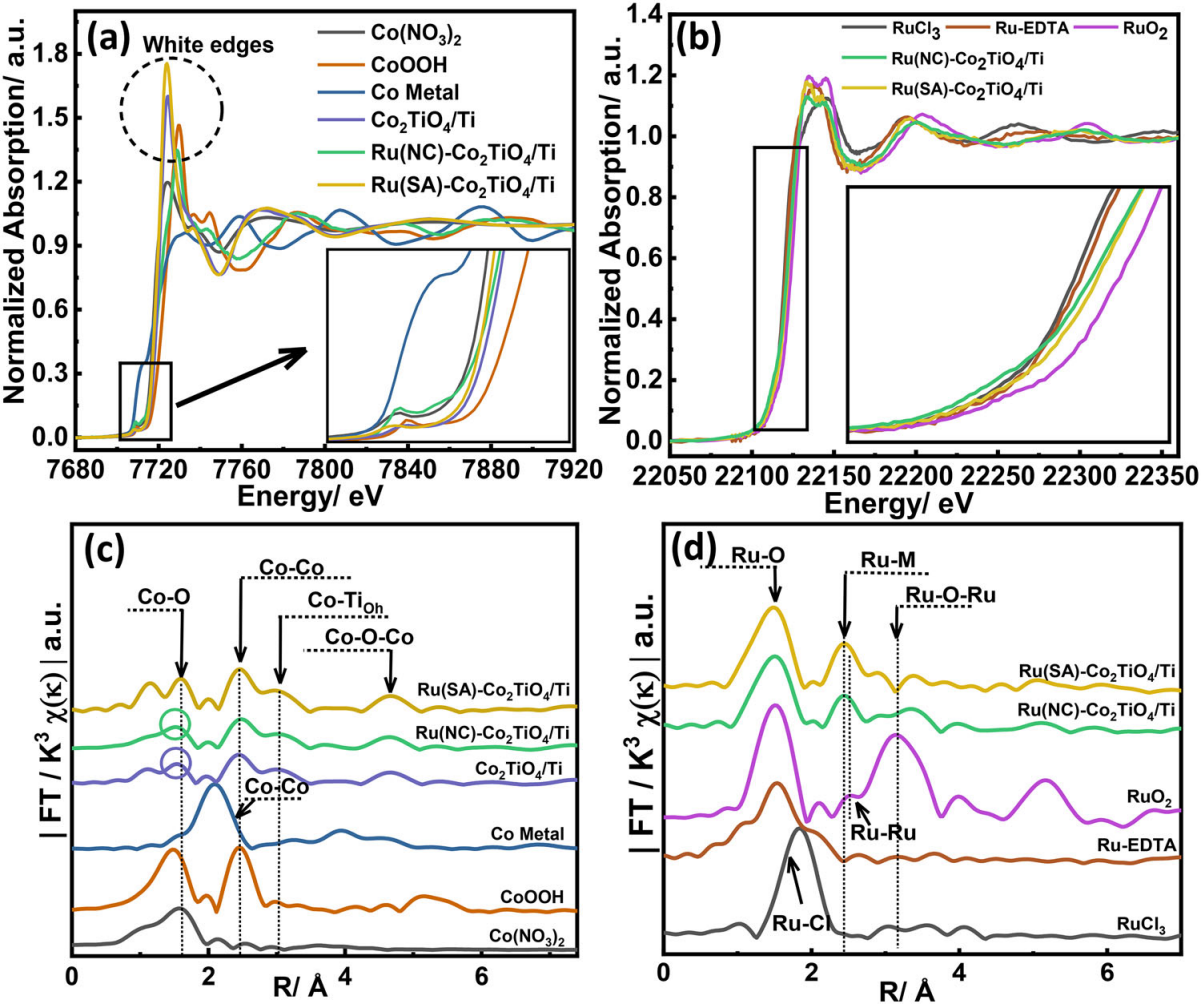
(a) Co K‐edge XANES spectra of Co(NO_3_)_2_, CoOOH, Co foil, Ru(NC)–Co_2_TiO_4_/Ti, Ru(SA)–Co_2_TiO_4_/Ti, and Co_2_TiO_4_/Ti. (b) Ru K‐edge XANES spectra of RuCl_3_, RuO_2_, as prepared Ru‐EDTA, Ru(NC)–Co_2_TiO_4_/Ti, and Ru(SA)–Co_2_TiO_4_/Ti. (c and d) the corresponding FT‐EXAFS spectra of (a and b), respectively.

Co K‐edge XANES (Figure 3a) revealed pre‐edge features at ∼7708 eV, characteristic of 1s→3d transitions and a non‐centrosymmetric coordination environment [43]. The Ru(SA)–Co_2_TiO_4_/Ti, Ru(NC)–Co_2_TiO_4_/Ti, and Co_2_TiO_4_/Ti catalysts all exhibited mixed Co^2^^+^/Co^3^^+^ states as evidenced by edge positions falling between those of Co^2^^+^ of Co(NO_3_)_2_ and Co^3^^+^ (CoOOH) reference compounds [43].

Compared to Co_2_TiO_4_/Ti, Ru incorporation in Ru(SA)–Co_2_TiO_4_/Ti induces a subtle negative edge shift and increased absorption intensity in the Co K‐edge XANES spectrum (Figure S8), indicating increased electron density around Co (Co^δ−^). The enhanced white‐line intensity is attributed to an increased 1s→4p transition probability associated with modified Co electronic structure, while the overall spectral line shape remains consistent with the Co_2_TiO_4_ spinel framework, indicating electronic modulation without lattice disruption. In contrast, Ru(NC)–Co_2_TiO_4_/Ti exhibits a positive shift toward higher energies, attributed to enhanced 1s→4p transitions and partial oxidation of Co species (Figure S8 and related discussion, Section S2.5 [44]). These spectroscopic trends are aligned with XPS analysis, which independently confirms electron redistribution around Co induced by Ru integration.

Ru K‐edge XANES (Figure 3b) shows absorption features at ∼22123 eV, originating from dipole‐allowed 1s→5p transitions. The white‐line energy position of Ru(SA)–Co_2_TiO_4_/Ti and Ru(NC)–Co_2_TiO_4_/Ti was positively shifted compared with RuCl_3_ and Ru‐EDTA but still lower than RuO_2_ (Figure 3b inset), indicating an intermediate oxidation state between Ru^3^^+^ and Ru^4^^+^ [45, 46]. In addition, both Ru‐containing catalysts exhibit higher white‐line intensity than RuCl_3_ and Ru‐EDTA but lower than RuO_2_, consistent with mixed‐valence Ru species and modified Ru–O electronic interactions within the oxide lattice. Quantitative edge‐energy analysis yields average Ru oxidation states of 3.56 for Ru(SA)–Co_2_TiO_4_/Ti and 3.35 for Ru(NC)–Co_2_TiO_4_/Ti (Figure S9). The higher average Ru valence in Ru(SA)–Co_2_TiO_4_/Ti compared to Ru(NC)–Co_2_TiO_4_/Ti reflects distinct Ru electronic environments. Ru‐EDTA exhibited an edge shift relatively close to RuCl_3_, with a calculated oxidation state of +3.13 based on edge‐energy analysis, consistent with literature [47].

To probe structural differences, k^3^‐weighted FT‐EXAFS spectra of Co and Ru‐K‐edges were analyzed (Figure 3c, d). Full EXAFS fitting details are provided in the Section S2.6, Figures S10–S13, and Tables S3‐S7. All EXAFS fits were performed in R‐space using appropriate k‐ and R‐ranges, with the number of fitting variables kept below the number of independent points to ensure statistical reliability, as summarized in Tables S3–S7.

For the Co K‐edge, EXAFS fitting of Co_2_TiO_4_/Ti confirmed key scattering paths associated with Co–O (∼1.92 Å), Co–Co (∼2.84 Å), and Co–Ti (∼3.33 Å), closely matching the orthorhombic Imma structure of Co_2_TiO_4_ (ICSD #5910130; R‐factor = 0.087) (Figure S10, Table S3). Minor deviations in the low‐R region were attributed to likely Co–N or Co–C contributions from surface/light‐atom scattering and disorder introduced during hydrothermal synthesis (e.g., urea/ethanol‐derived species), as discussed in Figure S11. The fitted amplitude reduction factor (S_0_^2^ ≈ 0.735) was used to scale nominal CNs, accounting for surface or defect‐related coordination damping (full fitting details are provided in the Section S2.6 and Table S3). Incorporation of Ru in Ru(SA)–Co_2_TiO_4_/Ti and Ru(NC)–Co_2_TiO_4_/Ti catalysts exhibits EXAFS features similar to those of Co_2_TiO_4_/Ti, except for slight increases in the Co─O peak position observed in the phase‐uncorrected FT‐EXAFS spectra (∼1.54–1.59 Å, Figure 3c). These values appear shorter than crystallographic Co–O bond lengths due to the absence of phase‐shift correction. This increase suggests local lattice distortion associated with Ru incorporation while preserving Co–O and Co–metal scattering paths, confirming the retention of the Co_2_TiO_4_ inverse spinel framework with stable Co^2^^+^ (T_d_) and Co^3^^+^ (O_h_) environments, as supported by XPS analysis. The Co K‐edge EXAFS of Ru(SA)–Co_2_TiO_4_/Ti and Ru(NC)–Co_2_TiO_4_/Ti also showed increased Debye–Waller factors (σ^2^ ≈ 0.0049–0.0050) and modest positive ΔR shifts (≈ +0.05–0.09 Å), indicating modest local structural disorder upon Ru incorporation.

Figure 3d presents the k^3^‐weighted Ru K‐edge FT‐EXAFS spectra for Ru(SA)–Co_2_TiO_4_/Ti, Ru(NC)–Co_2_TiO_4_/Ti, and reference materials, while Figure S12 and Tables S6–S7 summarize the corresponding EXAFS fitting results and parameters [48, 49, 50]. For Ru(SA)–Co_2_TiO_4_/Ti, the EXAFS spectrum shows only first‐shell Ru–O scattering paths at phase‐uncorrected distances of ∼1.97 Å (short), and ∼2.02 Å (long), consistent with a distorted octahedral Ru–O_6_ coordination environment, and no detectable Ru–O–Ru contributions near ∼3.15 Å, which is indicative of isolated Ru single‐atom sites (see SI discussion for Figure S12 and Tables S6–S7). The absence of Ru–Ru scattering beyond 3 Å confirms that no RuO_2_ nanoclusters are present, and the fitted S_0_^2^ ≈ 0.66 and R‐factor = 0.153 indicate good agreement between the experimental data and the fitted model.

In contrast, Ru(NC)–Co_2_TiO_4_/Ti displays clear contributions from Ru–O (∼1.94 Å short and ∼1.99 Å long), Ru–Ru (∼ 3.12  Å), and Ru–O–Ru (∼3.55 Å) scattering paths, consistent with the presence of RuO_2_ nanoclusters with limited periodicity [51]. The unusually low amplitude reduction factor (S_0_^2^ ≈ 0.43, Table S6) is most likely attributed to the nanocluster nature of RuO_2_, reflecting dampened scattering amplitudes caused by nanoscale disorder and reduced long‐range order typical of small, defect‐rich clusters [52]. The fitting for Ru(NC)–Co_2_TiO_4_/Ti yielded R‐factor ≈ 0.131 and N_indep_ = 18.4, supporting reliable fitting of multiple Ru–O and Ru–Ru scattering shells.

Additionally, RuO_2_ exhibits a characteristic Ru–Ru shoulder at ∼2.51 Å [53], which is absent in Ru(SA)–Co_2_TiO_4_/Ti and strongly attenuated in Ru(NC)–Co_2_TiO_4_/Ti. Instead, a peak appears at ∼2.42 Å, consistent with heterometallic Co–Ru [53] or Ti–Ru [54] interactions. The Ti L‐edge spectra (Figure S13) confirm preserved lattice symmetry in Ru(SA)–Co_2_TiO_4_/Ti, whereas RuCl_3_ incorporation in Ru(NC)–Co_2_TiO_4_/Ti leads to noticeable distortions in the Ti L_3_‐edge, indicating local symmetry disruptions [55]. These findings, supported by XRD, HRTEM, and Ru K‐edge EXAFS results, demonstrate that Ru is incorporated into the Co_2_TiO_4_ lattice predominantly as isolated Ru single‐atom sites in Ru(SA)–Co_2_TiO_4_/Ti, rather than forming RuO_2_ clusters.

### 2.1 Electrochemical Investigation

**FIGURE 4:**
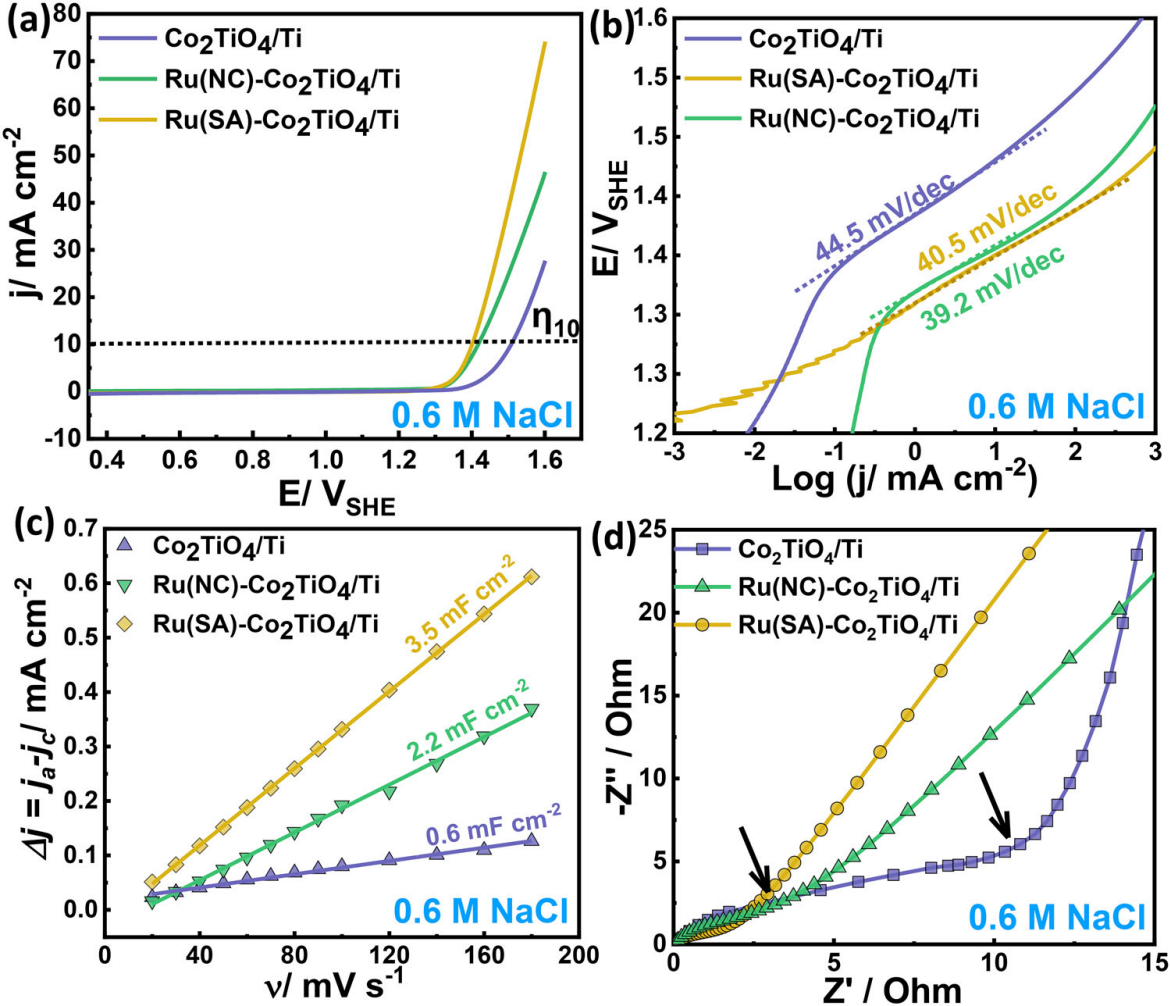
Electrocatalytic performance of Co_2_TiO_4_/Ti, Ru(NC)–Co_2_TiO_4_/Ti, and Ru(SA)–Co_2_TiO_4_/Ti catalysts with corresponding (a) LSV curves and (b) Tafel slopes in 0.6 M NaCl. The scan rate is 2 mV s^−^ [1]. (c) Linear plots of the CV scan rate vs capacitive currents ∆j = (j_anodic_‐j_cathodic_) of TiM, Co_2_TiO_4_/Ti, Ru(NC)–Co_2_TiO_4_/Ti, and Ru(SA)–Co_2_TiO_4_/Ti catalysts in 0.6 M NaCl electrolyte (R^2^ = 0.99). (d) Electrochemical impedance spectroscopy (EIS) spectra of the prepared Co_2_TiO_4_/Ti, Ru(NC)–Co_2_TiO_4_/Ti, and Ru(SA)–Co_2_TiO_4_/Ti catalysts in 0.6 M NaCl electrolyte at an exchange potential of 1.23 V_SHE_.

The performance of the prepared catalysts for CER was evaluated using voltammetric experiments in 0.6 M NaCl and 0.6 M NaClO_4_. These tests determined the voltage required to reach a geometric current density of 10 mA cm^−^^2^ (η_10_) (Figure 4a; Figure S14a) and the Tafel slopes (Figure 4b, Figure S14b). Table 1 summarizes parameters obtained from linear sweep voltammetry in both electrolytes. The NaCl concentration was chosen to approximate the chloride concentration relevant to seawater, focusing specifically on the competitive reaction between chlorine and oxygen evolution. Other cations commonly found in seawater (e.g., Mg^2^^+^, Ca^2^^+^, K^+^) were not included to isolate the role of Na^+^ and Cl^−^.

**TABLE 1:** Electrochemical kinetic parameters of the prepared catalysts determined in 0.6 M of either NaCl or NaClO_4._

Catalyst	*Tafel slope* (mV dec^−1^)	***η**_1_*_0_ (V_SHE_)	*C_dl_* ^b^ (mF cm^−2^)	*R_ct_* ^c^ (Ω)	*j/C_dl_* ^d^	*j_m_* ^e^
	NaCl/NaClO_4_ ^a^					
Co_2_TiO_4_/Ti	44.5/63.8	1.51/>1.6	0.6	37.4	46.17	−
Ru(NC)–Co_2_TiO_4_/Ti	40.5/39.1	1.42/1.55	2.2	6.27	21.14	244.74
Ru(SA)–Co_2_TiO_4_/Ti	39.2/57.5	1.39/1.51	3.5	3.02	20.91	915.00

^a^ The value obtained from similar electrolyte ionic strength of 0.6 m of either NaCl or NaClO_4_.

^b^ The electrochemical double‐layer capacitance (*C_dl_*) was determined by performing cyclic voltammetry (CV) in the non‐Faradaic region (0.30–0.50 V_SHE_).

^c^ The EIS was performed in 0.6 M NaCl, in the frequency range of 1–10^6^ Hz at 1.35 V_SHE_.

^d^ j/C_dl_ ​ (mA cm^−^^2^ mF^−^^1^) = ECSA‐normalized geometric current density in 0.6 M NaCl at 1.6 V_SHE_.

^e^ j_m_ (mA cm^−^^2^ mg_Ru_^−1^) = mass‐specific activity, calculated as j/Ru loading in 0.6 M NaCl at 1.6 V_SHE_

NaClO_4_ served as a chloride‐free control electrolyte, as it supports only oxygen evolution reaction (OER), whereas both chlorine evolution (CER) and OER can occur in NaCl [56]. In NaClO_4_, chlorine in ClO_4_^−^ oxyanions is already at its maximum oxidation state (+7), preventing further anodic oxidation [56]. Thus, OER exclusively dominates in NaClO_4_, while NaCl permits both CER and OER, assuming comparable oxygen evolution kinetics at similar ionic strength [57].

Electrolysis in 0.6 m NaCl (Figure 4a) produces higher geometric current densities compared to 0.6 M NaClO_4_ (chloride‐absent, Figure S14a), indicating concurrent CER and OER at all catalyst interfaces. Although isolating CER‐specific contributions from LSV alone requires comparison to the chloride‐free electrolyte, the present work focuses on benchmarking overall activity in both systems. The voltammograms reveal that both Ru‐containing catalysts achieve higher geometric current densities than Co_2_TiO_4_/Ti in NaCl and NaClO_4_ (Figure 4a; Figure S14a). For example, at 1.6 V_SHE_, Ru(NC)–Co_2_TiO_4_/Ti catalyst delivers 46.5 mA cm^−^^2^ in 0.6 M NaCl and 12.4 mA cm^−^^2^ in 0.6 M NaClO_4_, compared to 27.7 and 4.3 mA cm^−^^2^, respectively, for Co_2_TiO_4_/Ti. These results suggest that the incorporation of Ru enhances the catalyst's performance for both CER and OER. Furthermore, Ru(SA)–Co_2_TiO_4_/Ti exhibits the highest geometric current density (73.2 mA cm^−^^2^ at 1.6 V_SHE_) and the lowest η_10_ values (1.39 V_SHE_), highlighting its highly competitive catalytic performance.

The variation in catalyst performance can be attributed to the increase in the number of accessible active sites and the intrinsic activity toward the CER. The electrochemically active surface area (ECSA) [58] is proportional to the double‐layer capacitance (*C_dl_*), assuming a similar specific capacitance for all catalysts prepared under identical conditions, measured using cyclic voltammetry under non‐faradaic conditions (see Figure 4c and Figure S15). The *C_dl_* of Co_2_TiO_4_/Ti was 0.6 mF cm^−2^, increasing to 2.2 mF cm^−2^ for Ru(NC)–Co_2_TiO_4_/Ti and 3.5 mF cm^−2^ for Ru(SA)–Co_2_TiO_4_/Ti.

To assess whether the superior activity of Ru(SA)–Co_2_TiO_4_/Ti arises solely from a larger ECSA, geometric current densities were normalized by *C_dl_* (ECSA‐normalized activity) and by Ru loading (mass‐specific activity) (Table 1). Even after normalization, Ru(SA)–Co_2_TiO_4_/Ti shows activity comparable to or greater than Ru(NC)–Co_2_TiO_4_/Ti, despite having less than half the Ru loading (0.08 mg cm^−^^2^ vs. 0.19 mg cm^−^^2^). On a mass‐specific basis, Ru(SA)–Co_2_TiO_4_/Ti outperforms Ru(NC)–Co_2_TiO_4_/Ti by ∼3.7 fold, confirming that the enhanced CER performance primarily originates from the intrinsically higher catalytic activity of isolated Ru single‐atom sites rather than ECSA differences alone. This conclusion is consistent with structural evidence from SEM, HRTEM, XPS, and XAS [59, 60], which shows effective utilization of isolated Ru atoms in Ru(SA)–Co_2_TiO_4_/Ti compared with aggregated Ru domains in Ru(NC)–Co_2_TiO_4_/Ti.

To gain insights into electron exchange kinetics during CER, we performed electrochemical impedance spectroscopy (EIS) on Co_2_TiO_4_/Ti, Ru(NC)–Co_2_TiO_4_/Ti, and Ru(SA)–Co_2_TiO_4_/Ti catalysts in 0.6 M NaCl (Figure 4d). Nyquist plots showed two capacitive loops spanning high‐ and mid‐frequency regions, fitted using the equivalent circuits in Figure S16 (χ^2^ < 0.15). The charge transfer resistance (*R_ct_*) for Ru(SA)–Co_2_TiO_4_/Ti (3.0 Ω) is markedly lower than that of Co_2_TiO_4_/Ti (37.4 Ω) and remained lower than that of Ru(NC)–Co_2_TiO_4_/Ti (6.27 Ω), indicating significantly enhanced electron‐transfer kinetics due to the incorporation of isolated Ru atoms into the Co_2_TiO_4_ lattice.

Despite its higher Ru loading (0.19 mg cm^−2^), Ru(NC)–Co_2_TiO_4_/Ti (compared with 0.08 mg/cm^2^ for Ru(SA)–Co_2_TiO_4_/Ti, see ICP analysis in Section S1.3), this catalyst shows slower electron transfer kinetics and lower electrocatalytic performance. The Gerischer impedance (*G*), which describes diffusion‐related impedance associated with reactive intermediates such as chlorine‐ and oxygen‐containing species, shows a higher magnitude in Ru(SA)–Co_2_TiO_4_/Ti, corresponding to faster interfacial reaction kinetics and reduced mass‐transport resistance (see EIS discussion in Section S2.6.3) [61]. Thus, the high oxidizing activity of Ru(SA)–Co_2_TiO_4_/Ti is attributed to its atomic‐level Ru dispersion and the synergistic electronic interactions among Ru, Co, and Ti within the oxide lattice.

### 2.2 Electrocatalyst Stability

To evaluate catalyst stability, chronopotentiometry was performed with Ru(SA)–Co_2_TiO_4_/Ti, Ru(NC)–Co_2_TiO_4_/Ti, and Co_2_TiO_4_/Ti electrodes at 10 mA cm^−2^ in 0.6 M NaCl. The average potentials during the first hour of electrolysis were 1.38 V_SHE_ for Ru(SA)–Co_2_TiO_4_/Ti, 1.41 V_SHE_ for Ru(NC)–Co_2_TiO_4_/Ti, and 1.48 V_SHE_ for Co_2_TiO_4_/Ti. The corresponding overpotentials, 26.2 ± 2.9 mV, 49.5 ± 7.4 mV, and 126.4 ± 3.6 mV, were calculated using an equilibrium potential of 1.358 V_SHE_, a literature‐reported value under standard conditions (1 M Cl^−^, pH 0, 25 °C). This reference value was adopted to ensure consistency with previously reported studies rather than being determined experimentally in this work.

**FIGURE 5:**
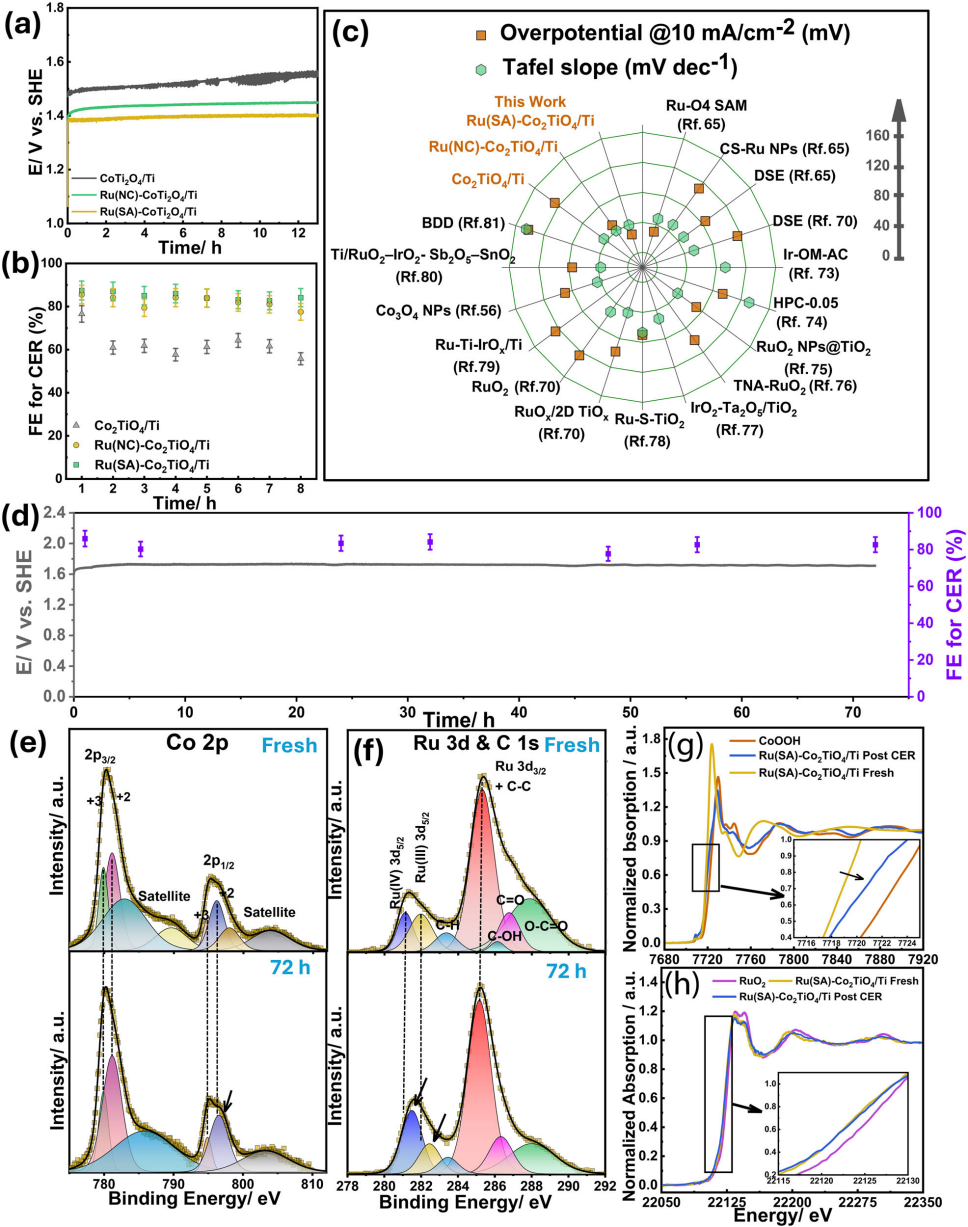
(a) Chronopotentiometry curves obtained from the electrocatalytic process of 0.6 M NaCl at 10 mA cm^−2^ using Co_2_TiO_4_/Ti, Ru(NC)–Co_2_TiO_4_/Ti, and Ru(SA)–Co_2_TiO_4_/Ti. (b) *FE_AC_* for CER (based on active chlorine production) for the data reported under the same experimental conditions as the chronopotentiometry results shown in (a). (c) Overpotentials and Tafel slopes of our prepared catalysts, along with some recently reported CER catalysts in references [56, 65, 70, 73, 74, 75, 76, 77, 78, 79, 80, 81]. (d) Chronopotentiometry curves obtained from the electrocatalytic process of 0.6 M NaCl at 20 mA cm^−2^ and FE of the CER using Ru(SA)–Co_2_TiO_4_/Ti catalyst. The HR‐XPS in (e) Co2p, and (f) C1s and Ru3d regions for the freshly prepared catalyst aligned in the top panel to compare with Co2p, C1s, and Ru3d regions of the same catalyst post 0.6 M NaCl electrolysis at 20 mA cm^−2^ for 72 h. The XANES spectra of (h) Co K‐edge and (g) Ru K‐edge, before and post 0.6 M NaCl electrolysis at 20 mA cm^−2^ for 72 h.

During 10 h of continuous operation, Ru(SA)–Co_2_TiO_4_/Ti maintained the lowest potential, increasing slightly to 1.39 V_SHE_​, while Ru(NC)–Co_2_TiO_4_/Ti and Co_2_TiO_4_/Ti increased to 1.43 and 1.54 V_SHE_ ​, respectively, with the rate of increase in the overpotential estimated to be 1.74, 2.32, and 4.93 mV h^−1^, respectively (Figure 5a).

Notably, the overpotential at 10 mA cm^−2^ for Ru(SA)–Co_2_TiO_4_/Ti is much lower than those reported in recent literature for similar catalysts, highlighting its superior catalytic performance (see Figure 5c and Table S8). Since the first report of atomically dispersed catalysts as a new class of CER electrocatalysts in 2020 [62], SACs supported on carbon and metal oxides have been extensively explored for CER [62, 63, 64, 65, 66, 67, 68]. Representative systems include carbon‐supported Pt‐SACs [63], oxide‐anchored Ru‐SACs [64, 65], coordination‐dependent Ir‐SACs on titanium oxides [66], and earth‐abundant transition‐metal SACs [67, 68]. In comparison with these benchmarks, Ru(SA)–Co_2_TiO_4_/Ti achieves an η_10_ of 26.2 mV while operating with an exceptionally low Ru loading of only 0.08 mg cm^−^^2^, placing it among the most efficient SAC‐based CER electrocatalysts reported.

For instance, when considering Ru loading as an evaluation parameter, only one report by Liu et al. [65] —featuring oxygen‐coordinated Ru–O_4_ single‐atom sites hosted on a carbon‐based substrate (Ru loading of 0.05 mg cm^−^^2^ compared with 0.08 mg cm^−^^2^ in our catalyst) —showed an overpotential of 30 mV at 10 mA cm^−^^2^, slightly higher than our reported value, and a Tafel slope of 48.2 mV dec^−1^. However, their Tafel slope (48.2 mV dec^−^^1^) is higher than the 39.2 mV dec^−^^1^ observed using our Ru(SA)–Co_2_TiO_4_/Ti catalyst—which may reflect a greater contribution from the oxygen evolution reaction (OER) in the mixed CER/OER pathway, as further discussed in a later section on electrokinetic analysis [69]. In contrast, a RuO_x_/2D TiO_x_‐based catalyst required a significantly higher Ru loading (0.255 mg cm^−^^2^) but still delivered inferior performance (η_10_ ≈ 98 mV, Tafel ≈ 43.6 mV dec^−^^1^ (see RuO_x_/2D TiO_x_ in Table S8) [65, 70]. These comparisons highlight the effectiveness of our Ru(III)‐EDTA‐based single‐atom incorporation strategy, enabling superior activity and stability with minimal Ru usage.

Dissolved active chlorine (DAC) was quantified using a standard DPD (N, N‐diethyl‐p‐phenylenediamine) colorimetry procedure [71], enabling calculation of the faradaic efficiency for active chlorine production (*FE_AC_*%) (see Figure 5b). Ru(SA)–Co_2_TiO_4_/Ti and Ru(NC)–Co_2_TiO_4_/Ti achieved the highest *FE_AC_* values at 87.4 ± 4.4% and 85.5 ± 4.3%, respectively, compared to 76.5% ± 3.9% for Co_2_TiO_4_/Ti. After 8 h of electrolysis, *FE_AC_*% decreased by only 3.7% for Ru(SA)–Co_2_TiO_4_/Ti, compared with 9.4% for Ru(NC)–Co_2_TiO_4_/Ti, whereas Co_2_TiO_4_/Ti decreased to 27.1 ± 1.36%. Long‐term testing at a higher applied current density of 20 mA cm^−2^ in a 0.6 M NaCl flow cell configuration over 72 h showed minimal potential increase from 1.68 V_SHE_​ after 1 h to 1.70 V_SHE_ after 72 h, while the faradaic efficiency for CER (*FE_CER_*) remained ∼82% (Figure 5d).

Changes in the Ru(SA)–Co_2_TiO_4_/Ti catalyst before and after 72 h of continuous CER were evaluated through XPS and XAS analyses. XPS survey scans confirmed retention of all constituting elements (Ru, Co, and Ti) with a weak Cl 2p signal indicative of adsorbed chloride (Figure S17). High‐resolution O 1s spectra showed an increase in lattice oxygen and a 0.05 eV negative shift in the O_latt._ peak consistent with increased oxygen vacancy concentration (Figure S18), which can enhance lattice‐mediated electron transfer [36]. The doublet peaks of Co 2p_1/2_ shifted slightly to higher energies post‐electrolysis, indicating increased positive charges (Co^δ+^) [39, 40] (Figure 5e and Figure S18). Ru 3d spectra exhibited positive shifts of +0.38 eV (Ru^4+^ 3d_5/2_) and +0.45 eV (Ru^3+^ 3d_5/2_), indicating a more electron‐deficient environment for the Ru atoms (Figure 5f). This electron deficiency, possibly due to an enhanced oxidation state or altered interactions with electronegative species such as Cl, as indicated in the survey scan [72].

XANES spectra of Ru(SA)–Co_2_TiO_4_/Ti at the Co K‐edge before and after the CER reaction (Figure 5h and inset) revealed a shift to higher energy, indicating a reduction in electron density near Co sites. This reduction, along with increased oxygen vacancies, facilitates electron transfer from the active metal site through the lattice oxygen network. After 72 h of CER, although the Ru K‐edge shows no significant shift (Figure 5g and inset), the FT‐EXAFS of Ru displayed a slightly broader feature around 1.9 Å (dashed line, Figure S19), coinciding with the Ru–Cl coordination distance observed in the RuCl_3_ reference (Figure 3d).

Taken together, the electrochemical stability data and post‐electrolysis spectroscopic analyses provide insight into the coordination environment of Ru under catalytic CER operating conditions. Post‐CER XPS reveals the emergence of a Cl 2p signal together with positive shifts in Ru 3d binding energies (Figure 5f and Figure S17), consistent with Ru–Cl interaction under anodic polarization. Ru K‐edge FT‐EXAFS collected after prolonged CER operation shows retention of the first‐shell Ru–O contribution along with a broadened feature near ∼1.9 Å, consistent with direct Ru–Cl coordination, without the emergence of Ru–Ru scattering. Together with EXAFS fitting features observed in the Figure S12 and Table S6, Section S2.6, including increased structural disorder and fitting instabilities associated with Ru–O coordination, these observations are consistent with dynamic coordination changes most likely involving oxygen and chloride at isolated Ru sites, rather than aggregation into bulk RuO_2_ domains or the formation of extended Ru–Ru coordination. This dynamic coordination behavior is consistent with the pH‐independent CER kinetics observed for Ru(SA)–Co_2_TiO_4_/Ti (Figure 7c) and supports a Ru–Cl adsorption pathway during electrolysis, in line with the Ru–Cl‐mediated CER mechanism proposed by Liu et al. [65], as further discussed in a later section.

### 2.3 Electrochemical Insights Into the CER Mechanism

Based on comparative electrochemical and spectroscopic analyses, the respective roles of the Ru species and the Co_2_TiO_4_ substrate can be clearly delineated. The Co_2_TiO_4_/Ti electrode exhibits substantially higher overpotential, lower chlorine selectivity, and inferior stability than Ru‐containing catalysts, indicating that the bare Co_2_TiO_4_ substrate is not intrinsically active for CER. Introduction of Ru dramatically enhances activity, confirming Ru as the primary catalytic center. Notably, despite its higher Ru loading, Ru(NC)–Co_2_TiO_4_/Ti shows inferior performance relative to Ru(SA)–Co_2_TiO_4_/Ti, demonstrating that catalytic activity is governed by the atomic dispersion and coordination environment of Ru rather than Ru content alone. The Co_2_TiO_4_ lattice, therefore, functions as an electronically active host that stabilizes isolated Ru sites and tunes their electronic structure via metal–support interactions, rather than serving as the primary active site.

The Tafel slope is a valuable tool for understanding the CER mechanism. Despite variations in ECSA, the Ru(SA)–Co_2_TiO_4_/Ti, Ru(NC)–Co_2_TiO_4_/Ti, and Co_2_TiO_4_/Ti catalysts exhibit similar Tafel slopes in the range of ∼40 mV dec^−1^ (Table 1), suggesting that the three catalysts follow a comparable rate‐determining charge‐transfer process [82, 83, 84, 85]. According to the Butler‐Volmer formalism, a Tafel slope near 59, 40, or 24 mV dec^−1^ for a chemically reversible multistep reaction with a single rate‐determining step (RDS) denotes the transfer of the first, second, or third electron, respectively [82, 83, 84, 85]. This suggests that the rate‐limiting step in the CER on these catalysts is the second electron transfer, with slight variations attributed to differences in catalyst composition.[82, 83, 84] The slightly higher Tafel slope for Co_2_TiO_4_/Ti suggests a greater contribution from mixed CER/OER pathways and fractional kinetic behavior, which can lower CER selectivity [86].

**FIGURE 6:**
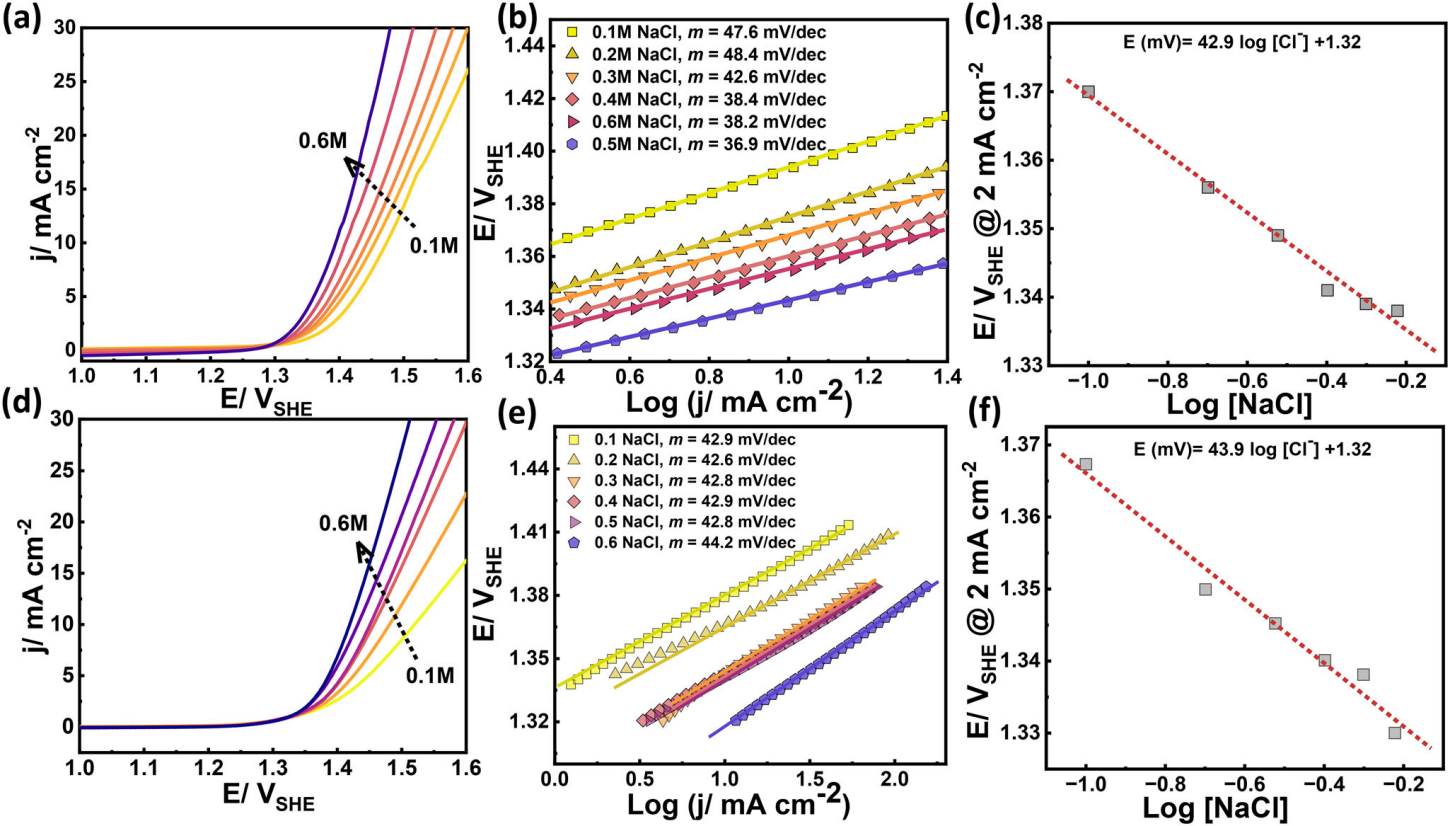
Comparison of (a–c) Ru(SA)–Co_2_TiO_4_/Ti catalyst with (d–f) Ru(NC)–Co_2_TiO_4_/Ti catalyst in terms of LSV at different NaCl concentrations (0.1–0.6 M) (a and d), the corresponding Tafel plots (b and e), and the variation of Log [NaCl] with potential (V_SHE_) at low current density (2 mA cm^−2^; near the exchange current) (c and f). The electrolyte was prepared using NaCl concentrations from 0.1 to 0.6 m while maintaining a constant ionic strength of 0.6 m with NaClO_4_ as a substitute salt.

The reaction order with respect to Cl^−^ was determined by varying the NaCl concentration from 0.1 to 0.6 M while maintaining a constant ionic strength of 0.6 M using NaClO_4_ as a substitute salt to eliminate the diffuse double‐layer effect [86]. Linear sweep voltammetry of the Ru(SA)–Co_2_TiO_4_/Ti and Ru(NC)–Co_2_TiO_4_/Ti catalysts at different Cl^−^ concentrations, along with the corresponding Tafel slopes, are shown in Figure 6b, e. The Tafel slope for the Ru(NC)–Co_2_TiO_4_/Ti catalyst was independent of the Cl^−^ concentration, maintaining a value of ∼42 mV dec^−1^ for concentrations from 0.1 to 0.6 M. However, for the Ru(SA)–Co_2_TiO_4_/Ti catalyst, the Tafel slope increased from ∼40 mV dec^−1^ at Cl^−^ concentrations of 0.3 M and above, to ∼48 mV dec^−1^ at 0.1 – 0.2 M. This variation indicates competition between the OER and CER at low Cl^−^ concentrations, potentially reducing chlorine production efficiency [69, 86].

At a low current density of 2 mA cm^−2^, the gradient in steady‐state potential with Cl^−^ concentration was similar for both Ru(SA)–Co_2_TiO_4_/Ti (42.9 mV M^−1^) and Ru(NC)–Co_2_TiO_4_/Ti (43.9 mV M^−1^) catalysts (Figure 6c, f). Considering both the Tafel slopes (Table 1) and [Cl^−^]‐dependent steady‐state potential, a first‐order electrochemical CER with respect to [Cl^−^] concentration can be inferred for both Ru(SA)–Co_2_TiO_4_/Ti and Ru(NC)–Co_2_TiO_4_/Ti catalysts (See Equations S5–S8).

**FIGURE 7:**
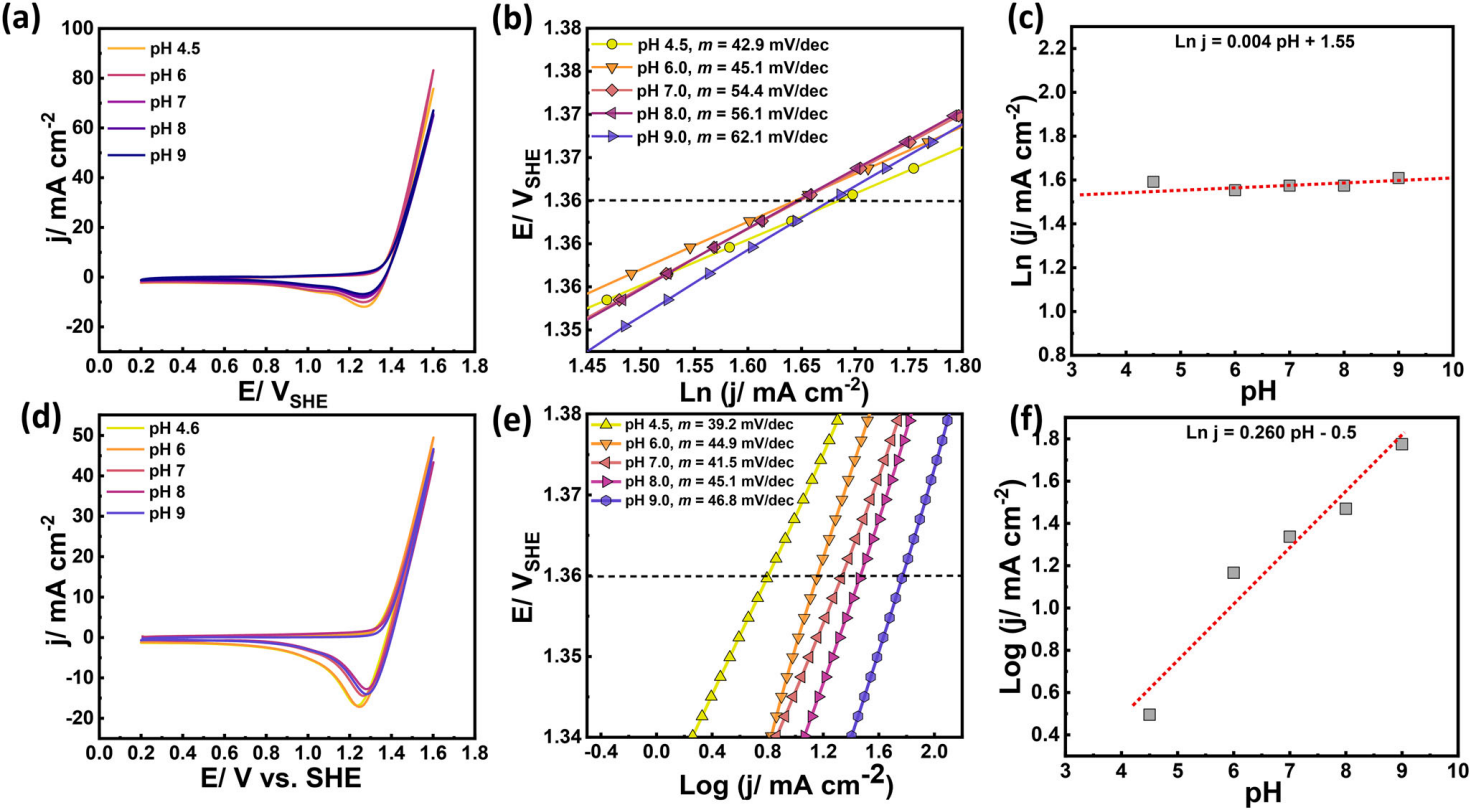
Comparison of (a–c) Ru(SA)–Co_2_TiO_4_/Ti catalyst with (d–f) Ru(NC)–Co_2_TiO_4_/Ti catalyst including cyclic voltammetry (a and d), the corresponding Tafel plots (b and e), and the geometric current density at a constant voltage of 1.36 V_SHE_ (close to the exchange current), as a function of pH (c and f) over the pH range 4.5 to 9.0. The electrolyte was prepared using 0.6 M NaCl in a phosphate buffer of 5 mm ionic strength.

The effect of pH on the CER in 0.6 M NaCl using Ru(SA)–Co_2_TiO_4_/Ti and Ru(NC)–Co_2_TiO_4_/Ti catalysts was studied over the pH range 4.5–9 using a phosphate buffer of 5 mM ionic strength. The voltammograms (Figure 7a, d) show minimal variation in overall oxidation current with pH across this range. However, analysis of the Tafel slopes (Figure 7b, e) reveals an increase in the slope with the Ru(SA)–Co_2_TiO_4_/Ti catalyst from 42.9 mV dec^−1^ at pH = 4.5 to 62.1 mV dec^−1^ at pH = 9.0. In contrast, the Tafel slope for the Ru(NC)–Co_2_TiO_4_/Ti catalyst remains relatively near ∼40 mV dec^−1^. The variation in the Tafel slope may be due to the parasitic OER contributions.

Figure 7c, f show the dependence of current density (indicative of the reaction rate) on pH at a constant potential of 1.36 V_SHE_, close to the electron exchange potential [7]. For the Ru(SA)–Co_2_TiO_4_/Ti catalyst, the current density appears to be independent of pH, while for the Ru(NC)–Co_2_TiO_4_/Ti catalyst, pH influences the electrode reaction rate. This indicates that protons participate in the CER for the Ru(NC)–Co_2_TiO_4_/Ti catalyst but not in the Ru(SA)–Co_2_TiO_4_/Ti catalyst.

These observations are in agreement with the observation made by Consonni et al. when comparing CER electrocatalysis using Ti plates coated with RuO_2_, either in polycrystalline or single crystal form [86]. They found that on polycrystalline RuO_2_, both the CER and OER had Tafel slopes of approximately 40 mV dec^−^^1^, with a proton‐involved mechanism. In contrast, for single‐crystal RuO_2_‐coated Ti, the CER Tafel slope was ∼ 40 mV dec^−^^1^, whereas OER Tafel increased to ∼60 mV dec^−1^, indicative of a proton‐independent CER mechanism [86]. Thus, the CER on Ru(SA)–Co_2_TiO_4_/Ti catalyst can be considered to have zero‐order dependence on H^+^, and the change in Tafel slopes with increasing the pH (4.5 to 9) is likely due to the competing pH‐dependent OER contributions [86].

Based on the post‐CER spectroscopic evidence confirming the persistence of atomically dispersed Ru sites with Ru–Cl coordination under CER conditions, the CER mechanism for Ru(SA)–Co_2_TiO_4_/Ti and Ru(NC)–Co_2_TiO_4_/Ti catalysts can be inferred from the voltammetry data, in particular the Tafel slopes shown in Figures 6 and 7, respectively. Both catalysts exhibit a Tafel slope of about 40 mV dec^−^^1^ in 0.6 M NaCl (pH = 4.5), which is consistent with a CER pathway in which the second electron‐transfer step is the apparent rate‐determining step (RDS) for the overall reaction 2Cl^−^ → Cl_2_ + 2e^−^.

For Ru(SA)–Co_2_TiO_4_/Ti, the pH‐independent current density at fixed potential (Figure 7c) indicates a zero‐order dependence on [H^+^], implying that its CER pathway does not involve proton‐coupled surface intermediates. The mechanism proposed by Krishtalik [87] appears most reasonable, involving initial adsorption and first electron exchange (Equation 1, where S represents a surface‐active site), followed by the electrochemical rate‐determining step of the second electron (Equation 2). This anodic rate‐determining step leads to the formation of adsorbed positively valent chlorine (S–Cl)^+^, which reacts with a second chloride ion to produce Cl_2_ in the subsequent fast step (Equation 3).

(1) $$S+Cl^{-}↔S-\mathrm{Cl}+e^{-}$$

(2) $$S-\mathrm{Cl}\to {\left(S-\mathrm{Cl}\right)}^{+}+e^{-}$$

(3) $${\left(S-\mathrm{Cl}\right)}^{+}+Cl^{-}↔Cl_{2}$$

In contrast, the CER mechanism on Ru(NC)–Co_2_TiO_4_/Ti is different, as evidenced by the pH dependence of the steady‐state current (Figure 7f). The pH dependence observed for Ru(NC)–Co_2_TiO_4_/Ti suggests a mechanism similar to those involving polycrystalline RuO_2_ surfaces, where proton‐involving steps and surface hydroxyl species significantly influence CER kinetics [86]. In this case, the involvement of protons in the CER is significant, and the electrode material may experience multiple simultaneous effects summarized by Consonni et al. [86]. Here, protons may adsorb on active sites, forming interfacial species including S−(OH)_ads_, S−(OH_2_^+^), or S−(O)_ads_ depending on pH, which act as sites for CER (see Equations (4), (5), (6)).

(4) $$S-{\left(\mathrm{OH}\right)}_{\mathrm{ads}}\to S-{\left(O\right)}_{\mathrm{ads}}+H^{+}+e^{-}$$

(5) $$S-{\left(O\right)}_{\mathrm{ads}}+Cl^{-}\to S-{\left(\mathrm{OCl}\right)}_{\mathrm{ads}}$$

(6) $$S-{\left(\mathrm{OCl}\right)}_{\mathrm{ads}}+H^{+}+Cl^{-}\to S-{\left(\mathrm{OH}\right)}_{\mathrm{ads}}+Cl_{2}+e^{-}$$

The superior CER performance of Ru(SA)–Co_2_TiO_4_/Ti is closely linked to its electronic structure and local coordination environment. The surface‐active sites in Ru(SA)–Co_2_TiO_4_/Ti, have a higher Ru oxidation state than those in Ru(NC)–Co_2_TiO_4_/Ti, along with increased Co^δ+^ levels and oxygen vacancies (see O1s and XANES spectra and discussion, Figures 2, 3), enhancing the electro‐adsorption and desorption of chloride intermediates. This structure facilitates a facile CER with pH‐independent kinetics. The active sites complete the CER step by drawing electrons from Cl^−^ species without requiring proton adsorption, thereby enhancing CER performance under anodic conditions [41, 88].

These electrochemical observations, together with XANES and EXAFS results, indicate that Ru atoms in Ru(SA)–Co_2_TiO_4_/Ti are present as isolated single atoms embedded within distorted Ru–O coordination environments that lack extended Ru–O–Ru connectivity, thereby increasing their electrophilicity and enhancing Cl^−^ adsorption [89]. This atomic configuration selectively stabilizes chloride‐containing intermediates while disfavoring the adsorption of oxygen‐containing intermediates (*OH, *O, *OOH), thereby suppressing the oxygen evolution reaction (OER) [90, 91]. Literature reports on Ru single‐atom catalysts indicate that isolated Ru atoms exhibit a lower free energy barrier for CER due to strong Cl^−^ affinity and suppressed O–O bond formation compared with Ru clusters [92]. This suppression of OER pathways is attributed to the fact that isolated Ru atoms lack adjacent metal centers required to stabilize or couple adsorbed O intermediates, a key step in OER [92].

Additional post‐CER spectroscopic measurements provide further support for this interpretation. FT‐EXAFS reveals a broad feature near ∼1.9 Å, consistent with Ru–Cl coordination, while Co K‐edge XANES shows an edge shift to higher energy, indicating reduced electron density around Co following CER. These changes point to partial oxygen substitution by chloride, direct Cl interaction at Ru sites, and charge redistribution within the Co_2_TiO_4_ lattice, which can stabilize CER intermediates while disfavoring OER. Importantly, no Ru–Ru scattering is detected in Ru(SA)–Co_2_TiO_4_/Ti, confirming the absence of Ru clustering and the preservation of atomic dispersion.

Consistent with these spectroscopic observations, atomic isolation likely contributes to the observed stability by disfavoring the formation of highly oxidized, dissolution‐prone oxygenated intermediates (e.g., O and OOH). Such intermediates have been identified by DFT‐based Pourbaix analyses as key drivers of oxide dissolution under anodic conditions [5]. Altogether, the combined electrochemical and spectroscopic evidence confirms that the EDTA‐assisted, lattice‐embedded Ru single atoms within the local Co_2_TiO_4_ structure enhance Cl^−^ adsorption while suppressing OER, thereby rationalizing the high CER activity, selectivity, and stability of Ru(SA)–Co_2_TiO_4_/Ti.

## 3 Conclusion

In this work, we demonstrated an EDTA‐complex‐assisted strategy to incorporate isolated Ru single atoms into a Co_2_TiO_4_/Ti oxide framework, enabling atomic‐scale dispersion. The synthesis leveraged electrostatic interactions between [Ru(EDTA)]^−^ and metal hydroxide precursors to achieve uniform atomic dispersion of Ru, overcoming agglomeration challenges interactions and offering a viable pathway for engineering oxide‐supported single‐atom catalysts. Comprehensive AC‐HAADF and X‐ray absorption analyses confirmed the presence of isolated, mixed‐valent Ru sites stabilized within the Co_2_TiO_4_ framework in Ru(SA)–Co_2_TiO_4_/Ti catalysts.

When used as electrocatalysts, the Ru(SA)–Co_2_TiO_4_/Ti catalyst significantly enhances performance for the chlorine evolution reaction (CER), relevant to seawater electrolysis. The specific catalytic architecture, with electron‐deficient, atomically dispersed Ru centers exhibiting strong Cl^−^ affinity, addresses the challenges of competing oxygen evolution reaction. Unlike conventional RuO_2_ catalysts that operate through OCl intermediates [14, 15, 57], the isolated Ru single‐atom sites in Ru(SA)–Co_2_TiO_4_/Ti favor chloride activation through direct Ru–Cl interaction rather than via Ru–O–Cl pathways, thereby decoupling CER from OER‐associated oxygen intermediates.

The Ru(SA)–Co_2_TiO_4_/Ti catalyst, with a low Ru loading of 0.08 mg cm^−^^2^, achieved an overpotential of 26.2 mV at 10 mA cm^−^^2^, a Tafel slope of 39.2 mV dec^−1^ and ∼87% selectivity toward active chlorine formation.

Notably, Ru(SA)–Co_2_TiO_4_/Ti outperforms state‐of‐the‐art CER systems in terms of overpotential, Tafel slope, and Ru utilization efficiency. While some reported systems achieve comparable activity, they often rely on expensive supports, higher synthesis temperatures, or significantly higher Ru loadings, limiting scalability and practical application. Our approach introduces atomically distributed Ru sites on metal‐oxide supports using a Ru(III)‐EDTA precursor within a controlled decomposition temperature window. This method serves as a source of Ru single atoms while preventing low‐temperature atomic migrations and high‐temperature agglomeration on metal‐oxide supports. Further work may benefit from advanced in situ spectroscopic and microscopic techniques to probe the structural evolution of Ru sites under reaction conditions. This synthetic strategy can be extended to other metal–oxide systems where controlled atomic dispersion is desired.

Moreover, electrochemical studies confirm that Ru(SA)–Co_2_TiO_4_/Ti exhibits pH‐independent CER kinetics, contributing to stable performance across varying conditions. Post‐electrolysis spectroscopic analyses further indicate that Ru remains atomically dispersed during CER and exhibits Ru–Cl coordination under anodic conditions, while retaining Ru–O lattice coordination, rather than aggregation into RuO_2_ domains, thereby rationalizing both the observed pH‐independent kinetics and the high selectivity toward chlorine evolution. The isolated Ru single‐atom centers, together with increased Co^δ+^ levels and lattice oxygen vacancies, promote efficient Cl^−^ adsorption and facilitate rapid Cl_2_ formation through a positive valent chlorine intermediate. These findings highlight the potential of Ru(SA)–Co_2_TiO_4_/Ti‐based catalysts for achieving stable, efficient, and selective CER under seawater‐relevant conditions, offering a promising pathway for advanced anode materials in sustainable energy applications.

## Author Contributions

N.G.Y. planned the study, executed experiments, analyzed results, and wrote the manuscript. P.K. assisted in catalyst synthesis, interpretation of characterization results, and data curation. M.G.K. and E.P.L.R. supervised the research, provided suggestions, and edited the manuscript.

## Conflicts of Interest

The authors declare no conflict of interest.

## Supporting information




**Supporting File**: smll72934‐sup‐0001‐SuppMat.docx.

## Data Availability

The data that support the findings of this study are available from the corresponding author upon reasonable request.
