# Supplementary material for: Single‐Atom Ruthenium Sites on Cobalt‐Titanium Surfaces for Efficient and Selective Chloride Electrolysis
Source: Small. 2026 Feb 23;22(21):e72934. doi: 10.1002/smll.72934 (PMC13081102; doi:10.1002/smll.72934)
Supplement: Supplementary file 1 — Supporting File: smll72934‐sup‐0001‐SuppMat.docx. [file SMLL-22-e72934-s001.docx]

**Supporting information**

**Single-Atom Ruthenium sites on Cobalt-Titanium Surfaces for Efficient and Selective Chloride Electrolysis**

Nael G. Yasri*, Pawan Kumar, Md Golam Kibria, Edward PL Roberts*

Department of Chemical and Petroleum Engineering, University of Calgary, 2500 University Drive NW, Calgary, AB T2N 1N4, Canada

*Email: Nael G. Yasri ([nael.yasri@ucalgary.ca](mailto:nael.yasri@ucalgary.ca)); Edward PL Roberts ([edward.roberts@ucalgary.ca](mailto:edward.roberts@ucalgary.ca))

Contents

Contents

[1. Experimental details 3](#_Toc217650295)

[1.1 Reagent and Materials 3](#_Toc217650296)

[1.2 Synthesis of Ru-EDTA 3](#_Toc217650297)

[1.3 Catalyst Preparation [Ru(SA)-Co_2_TiO_4_/Ti and Ru(NC)-Co_2_TiO_4_/Ti] 3](#_Toc217650298)

[1.4 Physicochemical Characterization 4](#_Toc217650299)

[2. Supporting Results 8](#_Toc217650300)

[2.1 Ru-EDTA characterization 8](#_Toc217650301)

[2.2 Raman spectroscopy of annealed catalysts. 11](#_Toc217650302)

[2.3 SEM 12](#_Toc217650303)

[2.4 X-ray diffraction (XRD) 13](#_Toc217650304)

[2.5 X-ray photoelectron spectroscopy (XPS) 15](#_Toc217650305)

[2.6 X-ray absorption spectroscopy (XAS) 20](#_Toc217650306)

[2.7 Extended X-ray Absorption Fine Structure (EXAFS) Analysis: 24](#_Toc217650307)

[2.8 Electrochemical investigation 35](#_Toc217650308)

[3. References 42](#_Toc217650309)

**Supplementary Figures**

[Figure S1. Raman spectra of the synthesized Ru-EDTA and Na-EDTA chelates and annealed catalysts. 9](#_Toc203077121)

[Figure S2. SEM images of the prepared catalysts. 12](#_Toc203077122)

[Figure S3. STEM image of Ru(NC)-Co_2_TiO_4_/Ti catalyst with the corresponding elemental distribution. 12](#_Toc203077123)

[Figure S4. HR-TEM and AC-HAADF-STEM images of Ru(SA)-Co_2_TiO_4_/Ti. 13](#_Toc203077124)

[Figure S5. XRD pattern of reference materials alone with and Co_2_TiO_4_/Ti catalyst. 15](#_Toc203077125)

[Figure S6. XPS survey scan of catalysts. 19](#_Toc203077126)

[Figure S7. High-resolution XPS spectra in N1s region for Ru(SA)-Co_2_TiO_4_/Ti and Co_2_TiO_4_/Ti. 20](#_Toc203077127)

[Figure S8. The XANES spectra at the pre-edge and white line of the Co K-edge in catalysts. 22](#_Toc203077128)

[Figure S9. Analysis of the Ru valence state from the corresponding Ru K‐edge absorption energies. 23](#_Toc203077129)

[Figure S10: FT-EXAFS for Co K-edge spectrum (k³-weighted, R-space) of the Co₂TiO₄/Ti catalyst 25](#_Toc203077130)

[Figure S11. EXAFS fitting results at the Co K-edge for Co₂TiO₄-based catalysts 28](#_Toc203077131)

[Figure S12. EXAFS fitting results at the Ru K-edge in the prepared catalysts. 31](#_Toc203077132)

[Figure S13. Ti L-edge XANES spectra of the prepared catalysts. 34](#_Toc203077133)

[Figure S14. Electrocatalytic process using the prepared catalysts with corresponding LSV curves and Tafel slopes in 0.6 M NaClO_4_.. 35](#_Toc203077134)

[Figure S15. Cyclic voltammograms at different scan rates (10-200 mV/s) for the prepared catalysts in 0.6 M NaCl electrolyte. 36](#_Toc203077135)

[Figure S16. Nyquist plots and their EIS equivalent fitting circuit model for the prepared catalysts in 0.6M NaCl electrolyte. 37](#_Toc203077136)

[Figure S17. XPS survey scan of Ru(SA)-Co_2_TiO_4_/Ti catalyst post 0.6M NaCl electrolysis 39](#_Toc203077137)

[Figure S18. The HR-XPS of Co2p, Ru3d and C1s, Ti2p, O1s, and N1s regions, in the freshely prepared and post- 72 h CER for Ru(SA)-Co_2_TiO_4_/Ti catalyst. 40](#_Toc203077138)

[Figure S19. FT-EXAFS spectra of Ru(SA)-Co_2_TiO_4_/Ti catalyst in Ru K-edge and Co K-edge regions before and after 72h electrocatalysis. 41](#_Toc203077139)

**Supplementary Tables**

[Table S1. Raman bands of Na_2_-EDTA and Ru–EDTA complexes (in cm^-1^) 10](#_Toc201838518)

[Table S2. Elemental composition of the as prepared catalysts determined using XPS survey scan (at%) 19](#_Toc201838519)

[Table S3. Fitting parameters for Co K-edge EXAFS of Co₂TiO₄/Ti.. 26](#_Toc201838520)

[Table S4. Fitting parameters for the Co–O first-shell path in Ru(SA)-Co_2_TiO_4_/Ti_,_ Ru(NC)-Co_2_TiO_4_/Ti catalysts, and Ru(SA)-Co_2_TiO_4_/Ti catalyst after CER. 27](#_Toc201838521)

[Table S4. Summary of Co K-edge EXAFS Fitting Parameters for Co₂TiO₄-based Catalysts 29](#_Toc201838522)

[Table S6. EXAFS fitting parameters for Ru K-edge of reference RuO₂, Ru(SA)-Co_2_TiO_4_/Ti, Ru(NC)-Co_2_TiO_4_/Ti, and Ru(SA)-Co_2_TiO_4_/Ti post-cer data.. 32](#_Toc201838523)

[Table S7: Summary of Ru K-edge EXAFS Fitting Parameters 33](#_Toc201838524)

[Table S8. Comparison of overpotentials obtained at 10 mA/cm^-2^ reported in recent literature for CER using various electrocatalysts. 38](#_Toc201838525)

# Experimental details

## Reagent and Materials

Ethylenediaminetetraacetic disodium salt; EDTA-Na_2_ (99%), ruthenium chloride trihydrate (RuCl_3_·3H_2_O), (99.9% trace metals basis), acetone (99%), diethyl ether (99%), cobalt (II) chloride hexahydrate (CoCl_2_·6H_2_O) (ACS reagent, 98%), urea CO(NH_2_)_2_ (ACS reagent, 99.0-100.5%) were purchased from Millipore Sigma. HPLC-grade solvents were used without purification, and aqueous solutions were prepared with 18.2 MΩ cm deionized water (EMD, Millipore).

## Synthesis of Ru-EDTA

The Ru-EDTA complex was prepared in a 5 mL aqueous medium using a stoichiometric ratio of 1 mM EDTA-Na₂ (0.372 g) and 1 mM RuCl₃·3H₂O (0.261 g). The solution was stirred vigorously until fully dissolved. The Ru-EDTA complex was then isolated via crystallization using 5 mL each of acetone and diethyl ether. The mixture was stirred at 600 rpm for 40 min and left to crystallize at room temperature for 24h. The resulting brown crystalline Ru-EDTA complex was characterized and used to prepare the Ru(SA)-Co_2_TiO_4_/Ti catalyst (see ICP-OES and Raman spectra).

## Catalyst Preparation [Ru(SA)-Co_2_TiO_4_/Ti and Ru(NC)-Co_2_TiO_4_/Ti]

The cobalt and titanium hydroxides growth on titanium mesh (TiM) was performed hydrothermally followed by pinning Ru sites either using RuCl_3_ or Ru-EDTA precursors. TiM of 400 mesh size with 0.15 mm thickness, 150 g m^−2^ surface density was used for hydrothermal preparation (BOLIN Corporation). The preparation was performed by dissolving 2 mmol of CoCl_2_·6H_2_O (0.26 mg) and 15 mmol (0.9 mg) of urea CO(NH_2_)_2_ in 60 mL ultrapure Milli-Q deionized water (resistivity >18.2 MΩ cm). A piece of TiM was cleaned in dilute HCl via sonication, ethanol washed, and immersed into the prepared solution of Co(II)/urea, followed by the addition of 40 mL ethanol, and sonicated for 30 min. The prepared solution and the TiM was than transferred to a 200 mL Teflon-lined stainless-steel autoclave, sealed, and heated to 150 °C for 24h. The autoclaved TiM was than washed with water to remove any loose particles. The prepared material at this stage is named hereafter as [Co-Ti(OH)_x_/Ti] and used subsequently for Ru surface modification. The TiM was used in this work to provide the mesh substrate of the catalyst as well as being a source of Ti element throughout the Co-Ti growth on the substrate. The hydrothermal treatment process of Co(II) chloride in the presence of urea and ethanol mixture was intended to provide mild reducing conditions. During the hydrothermal reaction, the pH increases (~ 10.1) due to urea decomposition (NH_3_ + HNCO) that enhances the alkalization of the system,^[1]^ nucleation of the atoms mixture, and growth of Co and Ti hydroxide.^[2]^ It has been reported that the slow release of NH_3_ due to the thermal decomposition of urea controls the hydrolysis and the growth of existing metal hydroxide nanoparticles.^[2]^

Ru was introduced onto a 5 × 5 cm² piece of Co-Ti(OH)_x_/Ti using two solutions, each with 10.0 mg Ru (metal basis) —either using 43.12 mg Ru-EDTA or 25.7 mg RuCl₃ dissolved in 10 mL methanol and 2 mL triethylamine. The samples were immersed, covered, and kept at room temperature for 24h. After immobilizing Ru, the electrodes were heated in air at 5 °C/min to 500 °C and held for 2h. A similar procedure was applied to Co-Ti(OH)x/Ti without Ru addition to obtain Co₂TiO₄/Ti. The resulting catalysts were named Ru(SA)-Co_2_TiO_4_/Ti for the catalyst modified with Ru-EDTA, Ru(NC)-Co_2_TiO_4_/Ti for the catalyst modified with RuCl_3_, and Co_2_TiO_4_/Ti for the annealing of Co-Ti(OH)_x_/Ti without Ru addition.

ICP-OES analysis was used to determine the surface content of Ru and Co. A 1 x 5 cm^2^ catalyst was digested using 5 mL 20% (v/v) aqua regia and diluted with water for the analysis. Ru contents of 0.08 mg/cm² and 0.19 mg/cm² were found on Ru(SA)-Co_2_TiO_4_/Ti and Ru(NC)-Co_2_TiO_4_/Ti, corresponding to 20.2% and 47.5% retention of the initial Ru, respectively. The Co content on Co_2_TiO_4_/Ti was 2.42 mg/cm².

## Physicochemical Characterization

### **Transmission electron microscopy (TEM)**

Microscopic TEM images were obtained by JEOL JEM200cf Atomic Resolution Microscope with a cold FEG type emission gun, operating at 200 kV (<https://confluence.nanofab.ualberta.ca/pages/viewpage.action?pageId=33196768>). This microscope is equipped with a Cs image corrector and a STEM high-angle annular dark-field detector (HAADF). The point resolution is 0.06 nm in TEM mode. The elemental mappings were obtained by STEM-energy dispersive X-ray spectroscopy (EDX). For TEM, a small portion of the catalyst was sonicated in ethanol, and a dilute dispersion was deposited on a carbon-coated copper grid.

### **X-ray photoelectron spectroscopy (XPS)**

XPS measurements were carried out using thin films of the developed catalyst deposited on the carbon tape. Room-temperature XPS experiments were performed at nanoFAB using a Kratos Axis (Ultra) spectrometer with monochromatized Al Kα (hυ = 1486.71 eV). Dual beam charge compensation was used for all measurements. The spectrometer was calibrated by the binding energy (84.0 eV) of Au 4f_7/2_ with reference to the Fermi level. The pressure of the analysis chamber during experiments is better than 5×10^-10^ Torr. A hemispherical electron-energy analyzer working at the pass energy of 20 eV was used to collect core-level spectra while survey spectrum within a range of binding energies from 0 to 1100 eV was collected at analyzer pass energy of 160 eV.

The binding energies of each element were calibrated with respect to adventitious carbons (C1s ≈284.8 eV).^[3]^ Data processing for peak fitting and deconvolution, as well as the determination of the atomic percentage of the respective elements, was performed using CasaXPS software (version 2.3.23PR1.0), following established protocols, utilizing a Shirley baseline, Lorentzian asymmetric (LA), and Gaussian-Lorentzian (GL) functions.^[4]^ Elemental quantification was carried out by considering the relative sensitivity factors (RSF) provided by the CasaXPS software. To minimize any charging effects during XPS measurements, the sample’s thin film was deposited on fluorine-doped tin oxide (FTO).

### **Raman spectroscopy**

Raman spectra were recorded using a WITec alpha 300 R Confocal Raman Microscope (WITec GmbH, Germany) equipped with a 532 nm laser, with a power of 3.5 mW cm^-2^ applied. The theoretical lateral resolution is 215 nm, and the spot size is 510 nm.

### **Scanning Electron Microscopy (SEM)**

The surface morphology of the prepared catalysts was studied by SEM-EDS using a Phenom ProX Desktop system, with an operating voltage of 15 kV.

### **X-ray diffraction (XRD)**

X-ray powder diffraction patterns were measured using a MiniFlex600 (Rigaku Corporation, Tokyo, Japan) equipped with Bragg-Brentano geometry and iron-filtered Cu Kα1,2 radiation (λ = 1.54056 Å) at 40 kV and 30 mA. Data were recorded at a scan rate of 0.05° s^−1^ with a 0.02° divergence over a 2θ angular range of 10–80°.

### **Soft X-ray absorption spectroscopy (sXAS)**

XAS of the Ti L-edges was conducted at the spherical grating monochromator (SGM) beamline 11ID-1 at the Canadian Light Source (CLS) synchrotron operating in the energy range of 250-2000 eV (<https://www.lightsource.ca/facilities/beamlines/cls/beamlines/sgm.php#SpectralRange>).^[5]^ For the measurement, the as-prepared catalysts and reference samples in powder forms were deposited on a double-sided carbon tape affixed to the sample holder. The sample holder was mounted in a vacuum chamber at 45° relative to both the detectors and the incident beam. The measurements were performed at an ultrahigh vacuum (~10^-6^ Torr) at room temperature. The samples were exposed to soft X-rays while keeping the spot size of 50- and 100-microns with the help of the Kirkpatrick-Baez mirror system. The energy and numbers of emitted photons were measured using Amptek silicon drift detectors (SDDs) having an energy resolution of ~100 eV. The partial fluorescence yield for each measurement was obtained by summing all emission lines from all SDDs.

### **X-ray absorption near edge structure (XANES) and extended X-ray absorption fine structure (EXAFS)**

The local chemical structure, oxidation state, and coordination environment of the materials were analyzed through X-ray absorption near edge structure (XANES) and extended X-ray absorption fine structure (EXAFS) techniques using hard X-rays energy source. The measurements were conducted using the 06ID-1 Hard X-ray MicroAnalysis (HXMA) beamline at the Canadian Light Source, which operates in the energy range of 5–40 keV. This beamline features a superconducting wiggler source and a photon flux of 10¹² photons per second at 12 keV. Catalysts and reference materials were evenly deposited onto Kapton® tape and mounted in a hollow plastic holder, which was secured on a sample socket for irradiation by the hard X-rays. The X-ray beam had a spot size of 0.8 mm x 1.5 mm, with a spectral resolution of 1×10^−4^. Energy calibration was achieved by measuring the absorption edge of standard samples. All measurements were carried out in transmission mode within energy ranges of 21,920–22,800 eV for Ru and 7,520–8,450 eV for Co.

The raw data were processed, normalized, and exported using the Athena software module within the IFEFFIT package. The k^3^-weighted EXAFS spectra were generated by subtracting the post-edge background from the total absorption and normalizing to the edge-raising step. The resulting k^3^-weighted χ(k) data for the Co and Ru K-edges in both the samples and standard references were Fourier transformed to real (R) space using a Hanning window (dk = 1.0 Å^−1^), enabling separation of EXAFS contributions from different coordination shells. Quantitative structural parameters around the central atoms were then extracted via least-squares fitting, performed in the Artemis module of the IFEFFIT software package.^[6]^

### **Electrolysis and Electrochemical measurements**

The electrolysis of chloride solution (0.6M) was conducted in a three-electrode electrochemical system of 100 mL volume using two identical electrodes (geometrical surface areas of 1 cm^2^) i.e., the prepared catalyst as the working electrode and TiM as the cathode conjugated with an Ag/AgCl reference electrode (3.5M KCl saturated with silver chloride, CH Instruments, Inc.). In some stated experiments, the counter electrode was replaced by a Pt wire (5 cm long and diameter 1.0 mm, Sigma-Aldrich). Chronoamperometry, linear sweep voltammetry (LSV), and electrochemical impedance spectroscopy (EIS) analysis were performed using an electrochemical workstation (PGSTAT302N, Metrohm Autolab, B.V., The Netherlands). The applied potentials were converted from Ag/AgCl (3.5M KCl) scale to the conventional standard hydrogen electrode (SHE) scale by applying the: $E_{SHE}=E_{Ag/AgCl (3.5 M KCl)}+0.205 V$. Unless otherwise specified, all electrochemical potentials reported herein are referenced to SHE. The liquid samples, collected at specified intervals from the electrolyte, were analyzed for total free chlorine using a pre-validated DPD (N,N-Diethyl-p-phenylenediamine) method with Free Chlorine Reagent Powder Kits and a pre-calibrated spectrophotometer from Hach Company/Hach Lange GmbH.^[7]^ The Faradaic efficiency of free chlorine production was evaluated using the following equation:

${FE}_{x}(\%)=\frac{Q_{CER}}{Q_{total}}=\frac{n_{{Cl}_{2}} {\left( 2 e^{-} \right)C}_{{Cl}_{2}}\left( mol L^{-1} \right)\times V\left( L \right)\times96485(C {mol}^{-1})}{i_{total}(A)\times t (s)}\times100$ Eq. 1

where $C_{{Cl}_{2}}$ is the concentration of the generated Cl_2_, V is the volume of the electrolyte, $n_{{Cl}_{2}}$ number of electrons (2) transferred to produce 1 mole of ${Cl}_{2}$, and 96,485 is Faraday constant, $i_{total}$ is the current measured during the reaction, and t is the electrocatalysis duration for CER.

# Supporting Results

## Ru-EDTA characterization

An inductively coupled plasma-optical emission spectrometry (ICP-OES, Thermo Fisher ICAP 7200) was used to obtain Ru content in the chelate. 50 mg Ru-EDTA complex was digested using 5 mL 20%(v/v) aqua regia and diluted accordingly to meet the ICP standard solution. The ICP analysis indicates a Ru content of 11.74 mg, about 98.8% of theoretically calculated Ru content for the EDTA-H-Ru-Cl (M.W. 425.3) complex.

Raman spectra of the brown crystalline solid confirm the Ru-EDTA chelation. **Figure S1** shows the Raman spectra of the prepared chelate and the EDTA-Na_2_, whereas the Raman bands are summarized in **Table S1**. Raman spectra for these types of compounds can be challenging to interpret due to their complexity, manifesting numerous features.^[8]^ To simplify the analysis, we initially confirmed that the pristine EDTA spectra align completely with those reported in the literature.^[8]^ Consequently, our focus was directed towards monitoring the existence of Ru–N, Ru–O, and the change in frequencies of the carboxylic bonding group in the pristine EDTA i.e., COOH (C=O) group.

Kanamori et al. ^[9]^ demonstrated that the 300–600 cm^−1^ Raman spectral region of Metal-EDTA is indicative of the coordination region. Specifically, M–N and M–O stretching modes, observed in the Ru-EDTA spectrum at 436.2 cm^−1^ and 602.1 cm^−1^, respectively, are absent in the free-EDTA salt. Additionally, the frequencies of the COOH (C=O) group notably exhibit high sensitivity to chelation, shifting from 1656 cm^−1^ in the free-EDTA salt to 1705 cm^−1^ in Ru-EDTA. Moreover, as illustrated in the zoomed-in view of **Figure S1**, two bands at 436 and 505 cm^−1^ are attributed to Ru–N stretching, serving as distinctive features of the coordination. These bands exhibit smaller frequency differences (Δω) in the free-EDTA salt compared with the Ru-EDTA complex, suggesting that the coordination in free-EDTA, unlike the Ru-EDTA complex, is either with a lighter element than Ru or involves no coordination.^[8]^

| **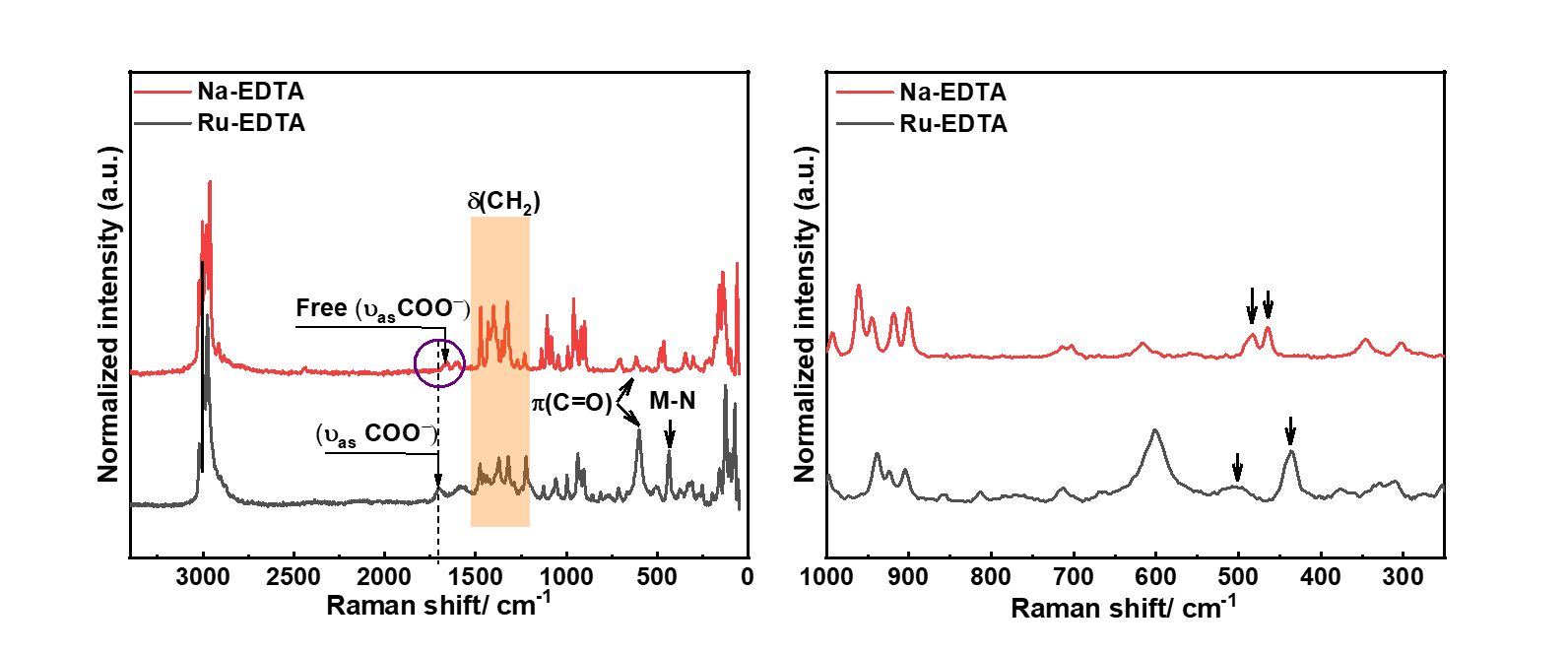**  a)  b) |
| --- |
|   c) |

**Figure S1. a-b)** Raman spectra of the synthesized Ru-EDTA and Na-EDTA chelates in two different frequency regions. **c)** Raman spectra of annealed Co₂TiO₄/Ti, Ru(NC)–Co₂TiO₄/Ti, and Ru(SA)–Co₂TiO₄/Ti catalysts compared with the Ru-EDTA precursor (Figure S1). The absence of Ru–N, Ru–O, and carboxylate-related vibrational features characteristic of Ru-EDTA confirms complete removal of the EDTA ligand after annealing. Differences in low-frequency lattice modes reflect distinct Ru dispersion states.

| Table S1. Raman bands of Na_2_-EDTA and Ru–EDTA complexes (in cm^-1^) |
| --- |
| Na_2_-EDTA  62s, 85sh, 98m, 133sh, 141s, 160s, 179w, 214vw, 231vw, 256vw, 278vw, 305w, 347w, 465m, 482m, 515vw, 559br, 617w, 704 w, 715w, 901m, 918m, 945m, 960s, 993 m, 1045 m, 1082 s, 1107 s, 1138 m, 1230 m, 1270 w, 1325 s, 1334 sh, 1354 w, 1379 sh, 1394 sh, 1402 s, 1413 sh, 1431 s, 1471 s, 1597 br, 1656 br, 2441 wbr, 2885 vw, 2914 w, 2960 vs, 2978 s, 2987 s, 3003 s, 3020 s. |
| Ru-EDTA  56m, 73 s, 102m, 125 vs, 158 m, 197 w, 224vw, 253 w, 310vw, 328 vw, 345 wsh, 359 vw, 374 vw, 436 s, 505 w, 602 s, 667 wsh, 712 w, 773 wbr, 814 w, 860 vw, 904m, 924m, 937m, 1997 m, 1059 m, 1076 wsh, 1126 m, 1201 sh, 1223 m, 1248 vw, 1290 wsh, 1319 w, 1373 w, 1394 wsh, 1421 vw, 1437 vw, 1456 vw, 1475 s, 1581 br, 1705 br, 2873 wbr, 2902 wbr, 2976 vs, 2997 vs, 3020 s. |
| vs, very strong; s, strong; m, medium; w, weak; vw, very weak; sh, shoulder; br, broad. |

## Raman spectroscopy of annealed catalysts.

As established in Section 2.1, the Raman spectrum of the Ru-EDTA complex (Figure S1) exhibits distinct vibrational features associated with EDTA coordination, including Ru–N and Ru–O stretching modes in the 300–600 cm⁻¹ region, as well as characteristic shifts of the carboxylate C=O group upon chelation. These ligand-related features serve as clear spectroscopic fingerprints of intact EDTA coordination.

In contrast, Raman spectra of the annealed Co₂TiO₄/Ti, Ru(NC)–Co₂TiO₄/Ti, and Ru(SA)–Co₂TiO₄/Ti catalysts (Figure S2) show none of the Ru–N, Ru–O, or carboxylate-related bands identified for Ru-EDTA in Figure S1. Instead, all annealed samples are dominated by metal–oxygen lattice vibrations below ~800 cm⁻¹, characteristic of oxide frameworks. The complete disappearance of EDTA-related vibrational modes confirms that the organic chelating ligand is fully decomposed and removed during the annealing treatment at 500 °C.

Minor differences in the low-frequency region between Ru(SA)–Co₂TiO₄/Ti and Ru(NC)–Co₂TiO₄/Ti are attributed to differences in Ru dispersion, with broader features in Ru(NC)–Co₂TiO₄/Ti consistent with RuO₂ nanocluster formation, while the Ru(SA)–Co₂TiO₄/Ti spectrum is consistent with atomically dispersed Ru species. Importantly, no Raman signatures attributable to residual EDTA are detected in either catalyst.

## SEM


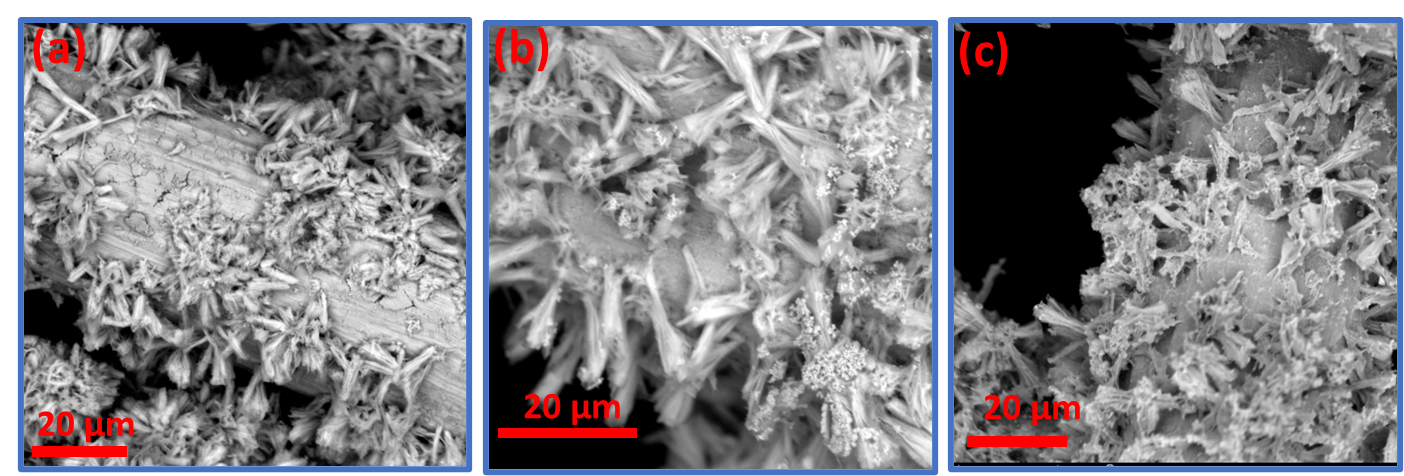


**Figure S2.**  SEM images of the prepared catalysts (a) Co_2_TiO_4_/Ti, (b) Ru(NC)-Co_2_TiO_4_/Ti, and (c) Ru(SA)-Co_2_TiO_4_/Ti.

**
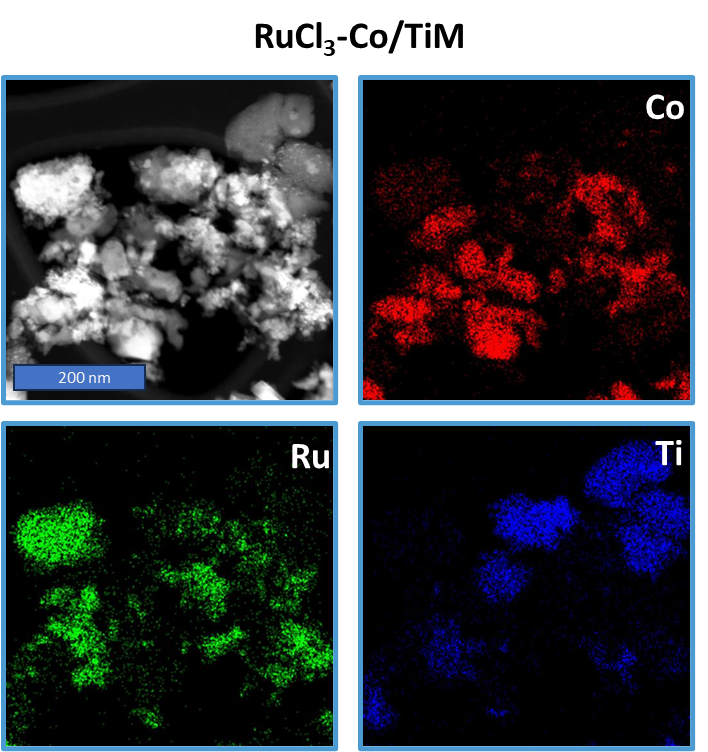
**

**Figure S3.** STEM image of Ru(NC)-Co_2_TiO_4_/Ti catalyst with the corresponding elemental distribution mapping of Co (red), Ru (green), and Ti (blue).

**
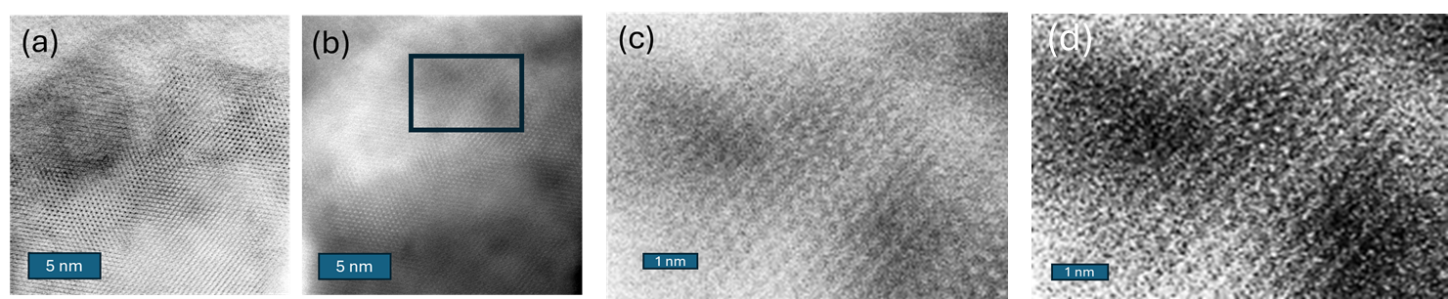
**

**Figure S4.** (a) HR-TEM and (b) AC-HAADF-STEM images of Ru(SA)-Co₂TiO₄/Ti at 5 nm scale bar. (c) An enlarged area of image (b) at a 1 nm scale bar, showing higher Z-contrast atoms consistent with isolated Ru single-atom sites anchored within the oxide framework. (d) Same image as in (c), but with enhanced contrast to better visualize the individual Ru single atoms.

## X-ray diffraction (XRD)

**Figure S5** presents the XRD spectra of the Co_2_TiO_4_/Ti catalyst in comparison with standard CIF spectra obtained from the Materials Project website (https://next-gen.materialsproject.org/), confirming key phase assignments.^[6c]^ The reference spectra were calculated using VESTA software, with the same instrumental parameters (Cu Kα radiation) to ensure consistency in comparison. The XRD spectra of the Co_2_TiO_4_/Ti substrate show diffraction peaks corresponding to the underlying metallic Ti (hcp) mesh at 2θ = 35.2° (100), 38.6° (002), 40.4° (101), and 53.3° (102), with no evidence of crystalline TiO_2_ phase. This likely rules out the formation of TiO_2_ or suggests a non-crystalline structure under the hydrothermal and annealing conditions used for catalyst preparation.^[10]^

The substrate also displays a peak at 35.7°, which corresponds to the most intense peak (100%) in Co_2_TiO_4_ at 35.2°, associated with the (311) plane. A diffraction feature near 36.9° is observed, which coincides with the intense (311) reflection of Co₃O₄ as well as a weaker reflection of Co₂TiO₄, indicating peak overlap between these spinel-related phases.

The introduction of Ru to form Ru(NC)-Co_2_TiO_4_/Ti catalysts resulted in slight variations in the XRD patterns (**Figure 1j**). The spectrum confirms the presence of RuO₂, with peaks at 2θ = 28.0° (110), 41.7° (111), 54.8° (211), and 57.2° (220). Additionally, a slight shift of the Co₃O₄ (311) peak to 2θ ~ 36.6° possible incorporation of Ru into Co-based oxide domains or lattice distortion associated with Ru-containing oxide formation.^[11]^ In contrast, the Ru(SA)–Co₂TiO₄/Ti spectrum excludes the presence of crystalline Ru-containing phases, as no additional diffraction peaks beyond those of Co₂TiO₄/Ti are observed. After introduction of the Ru–EDTA complex, no RuO₂ diffraction peaks are detected, indicating the absence of RuO₂ nanoclusters or other crystalline Ru aggregates and consistent with non-crystalline, atomically dispersed Ru species.^[12]^

Moreover, the diffraction peak in Co_2_TiO_4_/Ti at ~45.6°, attributed to the (400) planes of Co₂TiO₄ and Co₃O₄ (see **Figure S5**), is suppressed in Ru(SA)–Co₂TiO₄/Ti, likely associated with lattice distortion of the (400) planes induced by EDTA-mediated Ru dispersion. Additionally, the dominant RuO₂ (101) reflection near 35° is not clearly resolved in Ru(NC)–Co₂TiO₄/Ti, likely due to overlap with the Co₂TiO₄ (311) plane (~35.2°) and the low crystallinity of Ru nanoclusters.

**Figure S5.** XRD pattern **from bottom to top:** Ti metal (mp-72), RuO_2_ (mp-825), CoO ([mp-22408](https://next-gen.materialsproject.org/materials/mp-22408?formula=CoO)), Co_3_O_4_ (mp-18748), TiO_2_ (Anatase, mp-360), TiO_2_ (Brookite, mp-1814), TiO_2_ (Rutile, mp-1439), Co_2_TiO_4_ (inverse spinel, mp-36765, CoTiO_3_ (ilmenite, mp-19424), and Co_2_TiO_4_/Ti catalyst. CIF files were obtained directly from Material Project website and the XRD patterns were calculated using VESTA software.

## X-ray photoelectron spectroscopy (XPS)

The chemical composition and oxidation states of Ru, Co, and Ti in the as-prepared Ru(SA)-Co_2_TiO_4_/Ti, Ru(NC)-Co_2_TiO_4_/Ti, and Co_2_TiO_4_/Ti catalysts were determined using XPS analysis. Survey scans in **Figure S6** confirmed the presence of Ru, Co, and Ti on the catalyst surfaces, with comprehensive core-level peaks observed for metal species and light elements (C1s, N1s, O1s) as well as inner/sub-core-level peaks (OKLL, O2s, Co3s, Co3p, Ti2s), confirming the presence of all constituent elements.^[13]^ Elemental quantification from XPS survey scans (Table S2) indicates approximately 5.1 at% Co and 3.8 at% Ti on the surface of the Co₂TiO₄/Ti catalyst. High-resolution Ti 2p analysis shows that Ti is predominantly present as Ti(IV), accounting for ~80.7% of the Ti 2p spectral area (corresponding to ~2.8 at% Ti_IV_), with the remaining contribution attributed to metallic Ti originating from the underlying Ti mesh substrate within the XPS probing depth. In contrast, cobalt is exclusively present as oxidized Co²⁺/Co³⁺ species generated during hydrothermal synthesis, consistent with the mixed-valence cobalt chemistry reported for spinel cobalt titanates (See later discussion for HR XPS-Co). The oxidized Ti⁴⁺ component therefore represents Ti incorporated into the surface Co–Ti oxide framework. Together with XRD phase identification and high-resolution TEM (HRTEM) analysis revealing lattice fringes with an interplanar spacing of 2.56 Å, corresponding to the Co₂TiO₄ (311) plane, these results collectively provide clear evidence for the formation of Co₂TiO₄ on the Ti mesh substrate.

Noteworthy to mention that the method of Ru introduction influenced the surface/subsurface (~10 nm depth) exposure percentages of other elements; introducing Ru as RuCl_3_ reduced the surface exposure of Co and increased that of Ti, while using Ru-EDTA had minimal impact on the surface percentages of other elements compared to Co_2_TiO_4_/Ti. This difference is likely due to the formation of RuO₂ nanoclusters in the Ru(NC)-Co_2_TiO_4_/Ti catalyst, covering Co and making it less detectable in the subsurface layers.^[13]^

Carbon frequently appears in XPS spectra due to environmental contamination, sample handling, residual contaminants in the XPS detection chamber, and the inherent presence of carbon in many materials, known as adventitious carbon.^[14]^ The overlapping binding energies of Ru3d peaks with C1s (270–295 eV range) and Ru3p peaks with Ti2p (450–500 eV range) necessitate advanced fitting protocols for high-resolution XPS (HR-XPS) analysis of Ru spectra to accurately resolve and quantify these elements (**Figure 2**).^[14-15]^

**Figure 2.** shows the HR-XPS in (a) Co2p, (b) C1s & Ru3d, (c) O1s, and (d) Ti 2p region aligned of **lower to upper panel**: Ru(SA)–Co₂TiO₄/Ti, Ru(NC)–Co₂TiO₄/Ti, and Co_2_TiO_4_/Ti catalysts. High-resolution XPS spectra of catalysts in Co region (**Figure 2a**) revealed overlapping multiplet and satellite features characteristic of Co^2+^ and Co^3+^ in all catalysts.^[16]^ The asymmetric main peaks at 778-782 eV and 794-798 eV, adjacent to satellite peaks, correspond to Co2p_3/2_ and Co2p_1/2_, respectively, and can be deconvoluted into peaks attributed to Co^3+^ and Co^2+^, indicative of the spinel Co^3+/2+^ structure.^[16a]^ The Co2p spectrum for Co_2_TiO_4_/Ti shows Co2p_3/2_ at 780.6 eV and Co2p_1/2_ at 796.0 eV, with an energy spacing characteristic of Co in the 3+/2+ oxidation state and a calculated Co^3+/2+^ ratio of 0.43.^[16]^ The introduction of Ru in the Ru(NC)-Co_2_TiO_4_/Ti catalyst, increases the Co(III) level (Co^3+/2+^ ratio of 0.520), with the binding energy of Co2p_3/2_ and Co2p_1/2_ shifting to higher energies (781.1 eV and 797.1 eV, respectively) compared to Co_2_TiO_4_/Ti. In contrast, the Ru(SA)-Co_2_TiO_4_/Ti doublet peaks shift slightly to lower energies (780.4 and 795.6 eV, respectively) with a Co^3+/2+^ ratio of 0.514.^[17]^ This suggests that Co in Ru(NC)-Co_2_TiO_4_/Ti carry more positive charges (Co^δ⁺^), while Ru(SA)-Co_2_TiO_4_/Ti has more negative charges (Co^δ⁻^) as compared with Co in Co_2_TiO_4_/Ti. In cobalt-based catalysts, Co³⁺ is the active site, and studies indicate that increased Co^3+^ content enhances catalytic oxidation activity, crucial in electrocatalysis.^[18]^

The high-resolution XPS spectra of O1s (**Figure 2c**) for the Co_2_TiO_4_/Ti catalyst, compared with Ru(SA)-Co_2_TiO_4_/Ti and Ru(NC)-Co_2_TiO_4_/Ti catalysts, show three peaks at 530.2, 531.2, and 532.6 eV, corresponding to lattice oxygen (O_latt._), surface-chemisorbed oxygen (O_chemis_), and surface-physisorbed oxygen (O_phys_.), respectively. O_chemis_ is more active in oxidation reactions due to its higher mobility.^[19]^ The higher O_chemis_ rate in Ru(SA)-Co_2_TiO_4_/Ti (32.9%) compared to Co_2_TiO_4_/Ti (27.4%) and Ru(NC)-Co_2_TiO_4_/Ti (17.8%) likely enhances the catalytic oxidation performance. The negative shift of the O_latt_ peak in Ru(SA)-Co_2_TiO_4_/Ti and Ru(NC)-Co_2_TiO_4_/Ti by 0.12 and 0.14 eV, respectively, compared to Co_2_TiO_4_/Ti, indicates oxygen vacancies facilitating electron transfer from the active metal site through the O_latt_.^[20]^

Furthermore, the formation of metal-N coordination in the Co_2_TiO_4_/Ti and Ru(SA)-Co_2_TiO_4_/Ti catalysts can be confirmed by the HR-XPS analysis of the N1s region (**Figure S7)**. The N1s peak envelope for Ru(SA)-Co_2_TiO_4_/Ti (394-406 eV) can be deconvoluted into four distinct peaks, corresponding to graphitic (403.8 eV), pyrrolic (402.9 eV), metal-coordinated (400.4 eV) Metal-N-C, and pyridinic nitrogen (399.9 eV) (**Figure S7**).^[21]^ In contrast, the Co_2_TiO_4_/Ti catalyst showed only metal-coordinated N (400.1 eV) and pyrrolic nitrogen (402.5 eV). Notably, the Ru(NC)-Co_2_TiO_4_/Ti catalyst did not exhibit a distinguished N1s peak, possibly due to limitations in resolution. Since the Ru(SA)-Co_2_TiO_4_/Ti sample was prepared via the annealing of Ru-EDTA, the metal-coordinated N peak likely arises from coordination with metal centers (predominantly Ru and Co) introduced during EDTA-assisted synthesis.

The Ti spectra (**Figure 2d**) for the Co_2_TiO_4_/Ti catalyst reveal two main peaks at 459.0 eV and 464.8 eV, with an energy spacing (Δ_BE_) of 5.77 eV, characteristic of the Ti^4+^ oxidation state.^[22]^ Additionally, a weak convex feature, deconvoluted into a broad peak at 460.5 eV, is attributed to Ti in a lower oxidation state (Ti⁽ⁿ⁾), likely due to subsurface titanium not being fully oxidized during the calcination process.^[23]^ With Ru incorporation, both Ti2p peaks shifted slightly to lower binding energies: in the Ru(NC)-Co_2_TiO_4_/Ti catalyst, Ti2p_3/2_ shifted by ~0.2 eV and Ti2p_1/2_ by ~0.27 eV, while in Ru(SA)-Co_2_TiO_4_/Ti, Ti2p_3/2_ shifted by ~0.2 eV and Ti2p_1/2_ by ~0.33 eV. These shifts, with no significant changes in the Ti2p_1/2_ to Ti2p_3/2_ intensity ratio across samples, may be due to the presence of undercharged Ti (Ti^3+^), and enhanced charge transfer arising from electronic interaction between Ru species and the oxide lattice.^[24]^

Regarding HR-XPS of Ru element, despite the overlapping of Ru3d XPS spectra with those of C1s, Ru(SA)-Co_2_TiO_4_/Ti and Ru(NC)-Co_2_TiO_4_/Ti catalysts show peaks broadening adjacent to the main C1s peaks, absent in Co_2_TiO_4_/Ti, confirming Ru3d core-level photoemission (**Figure 2b)**.^[25]^ Deconvolution reveals components at approximately 281.1 and 281.9 eV for Ru(III) and Ru(IV), indicating Ru incorporation into the Co₂TiO₄ lattice of the spinel oxide.^[26]^

Comparing binding energies shows a shift for Ru(IV) in Ru(NC)-Co_2_TiO_4_/Ti to a higher energy (282.2 eV), suggesting that Ru in Ru(SA)-Co_2_TiO_4_/Ti is less electronegative than in Ru(NC)-Co_2_TiO_4_/Ti.^[25, 27]^ These observations, along with higher Co^δ⁺^ and oxygen vacancies (**Figure 2a and 2c**), result in electron-deficient isolated Ru centers in Ru(SA)-Co_2_TiO_4_/Ti, optimizing chloride intermediate adsorption/desorption, reducing thermodynamic energy barriers, and enhancing CER performance.^[24b, 28]^ Similar enhancements have been reported for electron-deficient metal oxides by introducing metals (Ir, Rh, Au, Ru) on MoO_3_ embedded in graphitic carbon layers.^[24c]^ This synergy between high-valent isolated metal centers and electron-deficient surface-active sites may provide high stability and superior CER catalytic performance.^[24b, 28]^


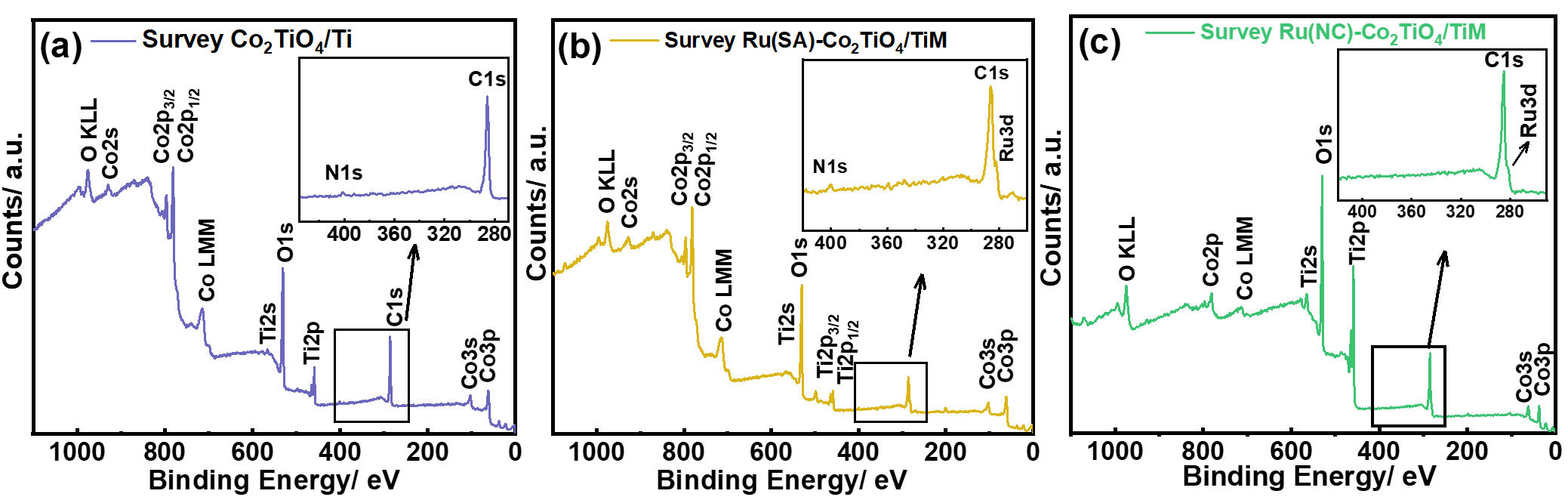


**Figure S6.** XPS survey scan of (a) Co_2_TiO_4_/Ti, (b) Ru(SA)-Co_2_TiO_4_/Ti, and (c) Ru(NC)-Co_2_TiO_4_/Ti catalysts.

|  |
| --- |

| Table S2*.* Elemental composition of the as prepared catalysts determined using XPS survey scan (at%) | | | | | | |
| --- | --- | --- | --- | --- | --- | --- |
| Sample | C | N | O | Co | Ti | Ru |
| Co_2_TiO_4_/Ti | 52.2 | 0.7 | 38.2 | 5.1 | 3.5 | -- |
| Ru(SA)-Co_2_TiO_4_/Ti | 48.2 | 0.9 | 38.9 | 5.4 | 3.1 | 3.41 |
| Ru(NC)-Co_2_TiO_4_/Ti | 48.5 | -- | 35.7 | 1.0 | 11.2 | 3.43 |


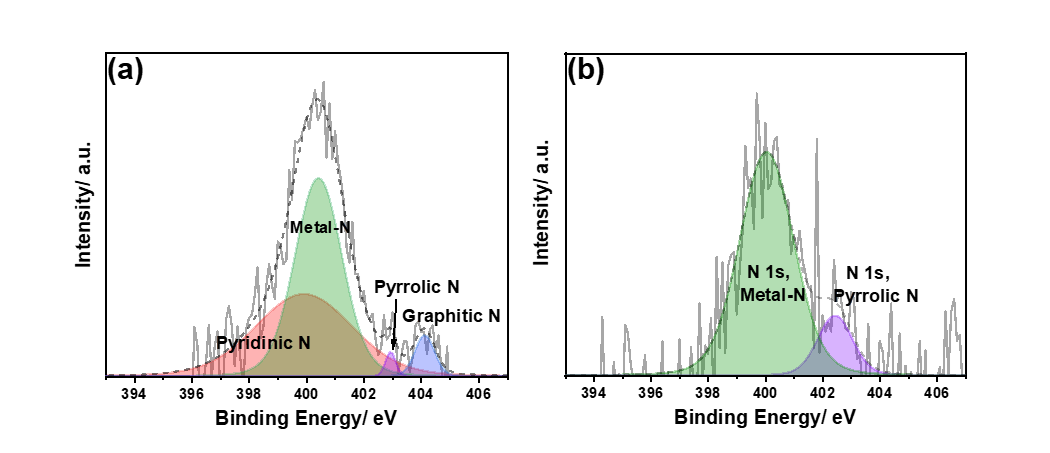


**Figure S7.** High-resolution XPS spectra in N1s region for (a) the Ru(SA)-Co_2_TiO_4_/Ti and (b) Co_2_TiO_4_/Ti catalysts.

## X-ray absorption spectroscopy (XAS)

X-ray absorption near-edge structure (XANES) spectroscopy was used to investigate the electronic structure of Co in Co₂TiO₄/Ti, and both Co and Ru in Ru(SA)-Co₂TiO₄/Ti and Ru(NC)-Co₂TiO₄/Ti catalysts. The normalized μ(E) vs. E (energy) curves for the catalysts and reference materials are shown in **Figures 3a and c**.

**Co K-edge Analysis:** XANES spectra of Co₂TiO₄/Ti exhibit a pre-edge signal at approximately 7708 eV (inset of **Figure 3a**), corresponding to 1s→3d transitions indicative of a locally distorted, non-centrosymmetric coordination environment.^[29]^ The rising edges of the prepared catalysts, located between Co^II^ and Co^III^, exhibit a mixed electronic state of Co²⁺/Co³⁺. The addition of Ru in Ru(SA)-Co_2_TiO_4_/Ti and Ru(NC)-Co_2_TiO_4_/Ti results in a slight negative shift in the rising edges compared to Co₂TiO₄/Ti, but the lines remain between Co^II^ and Co^III^. Notably, the Co₂TiO₄/Ti clone features and the intense white line in Ru(SA)-Co_2_TiO_4_/Ti suggest electronic interaction between Ru and the Co₂TiO₄ lattice formed after annealing. This structure is similar to Co₂TiO₄/Ti but exhibits a negative shift in the rising line and an increased intensity, suggesting enhanced negative charge density on Co (Co^δ⁻^) compared to Co in Co₂TiO₄/Ti (**Figure S8**). A similar increase in negative charges was concluded from the XPS analysis discussed in a previous section.

The increase in intensity of the edge may be due to the increase in electron transitions of the partially occupied p-band. However, there was no indication of a change in the structure when compared with pristine Co₂TiO₄/Ti. The Ru(NC)-Co_2_TiO_4_/Ti catalyst reveals significant changes in the electronic structure of cobalt upon the introduction of RuCl₃. The spectrum shows a pronounced shoulder in the rising edge, attributed to 1s→4p electron transitions (**Figure S8**), indicative of a structural change.^[30]^ The observed positive shift in the white line intensity suggests that the presence of Ru induces electronic charge redistribution within the Co₂TiO₄/Ti host lattice, increasing the positive charge of cobalt species, a conclusion also supported by XPS analysis.

**Ru K-edge Analysis:** XANES spectra at the Ru K-edge (**Figure 3b**) revealed absorption features at approximately 22123 eV, associated with dipole-allowed 1s→5p transitions. In contrast, the pre-edge features are not observed, which may be due to the forbidden 1s→4d transitions in certain structures (forbidden in dipole structures but allowed in quadrupole structures).^[31]^ The white line intensity of Ru(SA)-Co_2_TiO_4_/Ti and Ru(NC)-Co_2_TiO_4_/Ti was significantly higher than RuCl₃ and Ru-EDTA but remained lower than RuO₂ (**Figure 3b inset**). This observation suggests that infused Ru atoms in the Ru(SA)-Co_2_TiO_4_/Ti and Ru(NC)-Co_2_TiO_4_/Ti catalysts exists in a mixed-valence Ru³⁺/Ru⁴⁺ state.^[32]^ The average Ru valence state, estimated by comparing absorption edge energies with those of RuCl₃ (Ru³⁺) and RuO₂ (Ru⁴⁺) reference compounds, were determined to be 3.56 for Ru(SA)–Co₂TiO₄/Ti and 3.35 for Ru(NC)–Co₂TiO₄/Ti (**Figure S9**). The value of 3.56 for Ru(SA)-Co₂TiO₄/Ti indicates a mixed-valence state with Ru predominantly in an oxidized form close to Ru⁴⁺, while the lower value of 3.35 for Ru(NC)-Co₂TiO₄/Ti suggests a significant contribution from reduced Ru species (e.g., Ru³⁺), likely arising from partial reduction or structural disorder within the RuO₂ nanoclusters formed in the Ru(NC) catalyst. These observations, along with the modest positive shift in Co oxidation state observed for Ru(NC)–Co₂TiO₄/Ti—contrasted with the negligible or slightly negative shift in Ru(SA)–Co₂TiO₄/Ti—suggest a stronger electronic interaction and charge redistribution at the metal–oxide interface in the nanocluster system. This differential behavior reflects how Ru incorporation modulates the electronic environment of cobalt, with the extent of interaction varying between nanocluster and single-atom Ru species. Collectively, these findings support the hypothesis that the degree of Ru–Co₂TiO₄ electronic coupling is governed by the dispersion and structural form of the Ru species. Moreover, the XANES spectra of Ru-EDTA (Figure 3b inset) reveal a negative shift in the absorption edge compared to RuCl₃, corresponding to an oxidation state of +3.13, which aligns with the literature.^[33]^

**Figure S8.** The XANES spectra at the pre-edge and white line of the Co K-edge for Co_2_TiO_4_/Ti after Ru addition in the Ru(NC)-Co_2_TiO_4_/Ti and Ru(SA)-Co_2_TiO_4_/Ti catalysts.


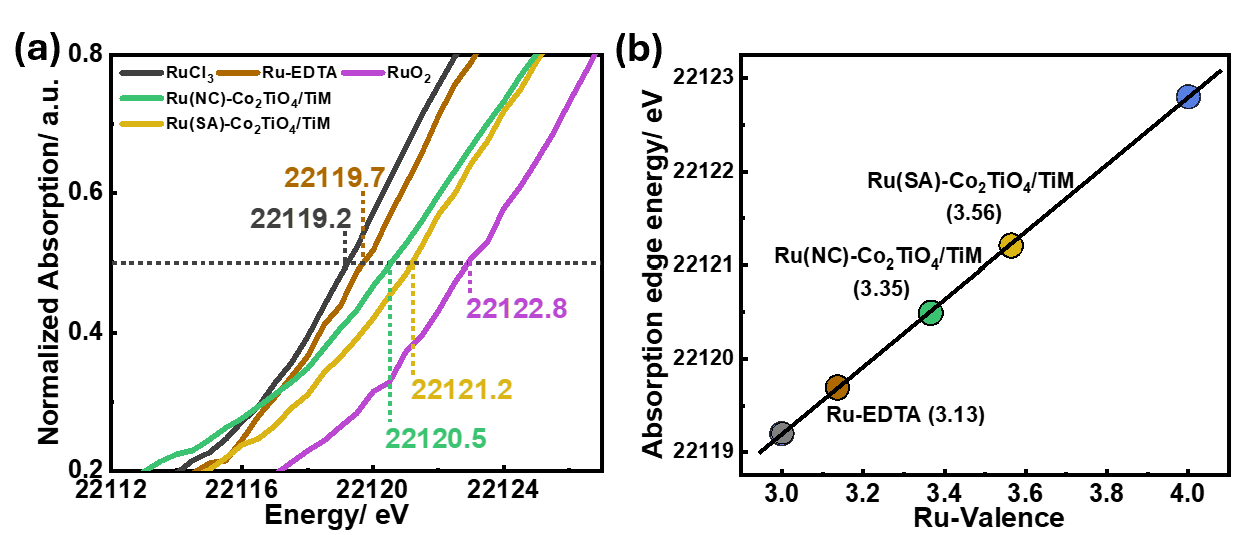


**Figure S9.** Analysis of the Ru valence state from the corresponding Ru K‐edge absorption energies.

## Extended X-ray Absorption Fine Structure (EXAFS) Analysis:

To further verify the structure of the as-prepared catalysts and investigate differences in their coordinative geometry, extended X-ray absorption fine structure (EXAFS) spectra were analyzed using k³-weighted Fourier transforms (FTs) of the spectra (without phase correction). The FT-EXAFS oscillations of the Co and Ru K-edges in the catalysts and corresponding reference materials are shown in **Figure 3c-d**. The prepared catalysts and the corresponding reference materials were fitted against their crystallographic information files (CIF) using the ATHENA and ARTEMIS modules of the IFEFFIT package and are presented in **Figures S10-13**, with corresponding R-factors listed in **Table S3-S7**.

In the Artemis fitting procedure for Co K-edge EXAFS of Co₂TiO₄/Ti (**Table S3**), scattering paths were generated using the crystallographic information file (CIF) of bulk Co₂TiO₄ (ICSD #5910130), and the coordination numbers (N) for the Co–O and Co–Co paths were initially set to 2 for modeling purposes. These were then scaled by the fitted amplitude reduction factor (amp ≈ S₀² ≈ 0.735) to approximate effective CNs, accounting for deviations from ideal bulk structure due to surface effects, nanoscale disorder, or partial coordination—common in supported or defective materials.^[34]^ In the Co₂TiO₄ spinel-like crystal structure, Ti occupies octahedral (O_h_) sites forming TiO₆ units that share corners and edges with CoO₄ tetrahedra (T_d_) and CoO₆ octahedra (O_h_).^[6b, 6c, 35]^ Literature reports Co–O bond lengths of ~1.88 Å in both tetrahedral (CN = 4) and octahedral (CN = 6) sites, while longer Co–metal paths (Co–Co or Co–Ti) appear in the range of 3.16–3.52 Å depending on the geometry (T_d_–O_h_ or O_h_–O_h_).^[36]^ In our Co K-edge EXAFS spectra for the Co₂TiO₄/Ti catalyst fitted against the crystallographic model of Co₂TiO₄ (**Figure S10 and Table S3**, uncorrected for phase shift), Co–O scattering paths are observed at ~1.6 Å and ~1.96 Å (phase-shifted), while Co–metal paths appear at ~2.45 Å and ~3.0 Å. The overall coordination environment is consistent with a mixed-valent spinel framework, where Co²⁺ occupies both T_d_ and O_h_ sites and Co³⁺ and Ti⁴⁺ reside in O_h_ sites.^[37]^ Additional features such as possible Co–N or Co–C scattering contributions may contribute to first-shell misfits, likely arising from residual urea and ethanol used in the hydrothermal preparation.

These paths can overlap with Co–O scattering, as indicated by deviations in the R region of the FT spectrum (**Figure S10**). It should be also noted that the hydrothermally grown Co₂TiO₄ layer on the Ti substrate may not exhibit the same degree of long-range crystallinity as bulk or pure-phase spinel references. The presence of the metallic Ti substrate, interface effects, and possible growth discontinuities can lead to local disorder or partial crystallinity, which is reflected in the EXAFS-derived coordination environments. The EXAFS fitting of the Co₂TiO₄/Ti catalyst confirms the presence of key scattering paths associated with Co–O, Co–Co, and Co–Ti interactions. The experimental data aligns well with the theoretical Co₂TiO₄ structure derived from the CIF (orthorhombic *Imma* space group),^[36]^ yielding a low R-factor of 0.087 (Figure S10, Table S3).^[6]^


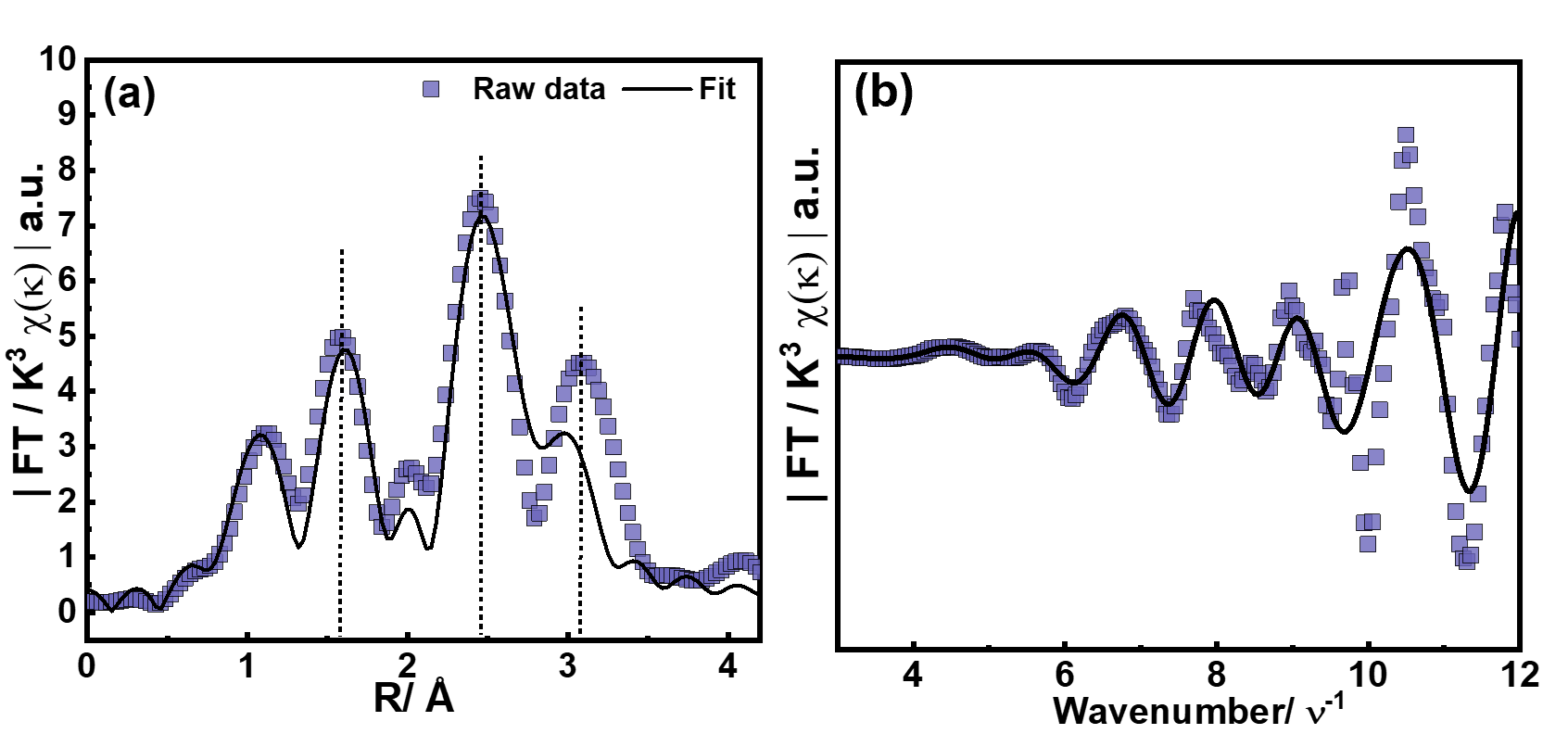


**Figure S10**: **(a)** Fourier-transformed Co K-edge EXAFS spectrum (k³-weighted, R-space) of the Co₂TiO₄/Ti catalyst, overlaid with the best-fit model derived from the Co₂TiO₄ CIF structure (ICSD #5910130). Dashed vertical lines indicate first- and second-shell coordination ranges.
**(b)** Corresponding k-space EXAFS spectrum (χ(k), weighted) and fit over the 3.5–12 Å⁻¹ range. The good agreement between data and fit in both k- and R-space confirms the preservation of the Co₂TiO₄ spinel structure on the Ti mesh substrate. The fit yielded an R-factor of 0.087, indicating high fidelity between experimental and modeled spectra.

| **Table S3***.* Fitting parameters for Co K-edge EXAFS of Co₂TiO₄/Ti. Coordination numbers (N) listed are model values used in the Artemis path list and were set to 2 for the Co–O first-shell and Co-Co second path to improve fit stability. The effective coordination numbers corresponding to crystallographic environments can be approximated by scaling the model CNs with the fitted amplitude reduction factor (S₀² ≈ 0.735).  ΔR indicates the fitted deviation from reference bond distances. The best fit achieved an R-factor of 0.087, indicating excellent agreement between model and experimental spectra. | | | | | | |
| --- | --- | --- | --- | --- | --- | --- |
| Path | N (CN) | R (Å) | ΔR (Å) | σ² (Å²) | E₀ (eV) | S₀² |
| Co–O (1st shell) | 2.0 | 1.92 | –0.09 | 0.0028 | –0.49 | 0.735 |
| Co–Co (2nd shell) | 2.0 | 2.84 | –0.14 | 0.0033 | – | – |
| Co–Ti (2nd shell) | 2.0 | 3.33 | –0.16 | 0.0031 | – | – |
| Co–O–Co (multiple path) | 12.0 | 3.34 | –0.16 | 0.0148 | – | – |
| *Notes:* Crystallographic Co–O coordination in Co₂TiO₄ (ICSD #5910130) is 6, but was modeled as 2 in the EXAFS path list to reflect amplitude damping effects and increased structural disorder associated with the nanoscale surface environment. R values are fitted interatomic distances obtained from Artemis/FEFF modeling (phase-corrected); FT peak positions shown in  figures are phase-uncorrected. The EXAFS fits were performed in R-space with *k* = 2.4–12.2 Å⁻¹ and *R* = 1.4–3.2 Å. | | | | | | |

The Co K-edge EXAFS spectra for Ru(SA)-Co_2_TiO_4_/Ti and Ru(NC)-Co_2_TiO_4_/Ti display similar overall features to Co₂TiO₄/Ti (Figure 3c), but show slightly increased Co–O and Co–metal distances (ΔR ≈ +0.05–0.09 Å) and higher Debye–Waller factors (σ² ≈ 0.0049–0.0050), indicating modest local structural disorder following Ru incorporation (**Table S4 and Figure S11**). The Ru(SA)-Co₂TiO₄/Ti catalyst retains a spinel-like local coordination environment with reduced apparent coordination numbers (CN ≈ 2), consistent with lattice-embedded, atomically dispersed Ru that does not significantly perturb the Co₂TiO₄ framework. In contrast, Ru(NC)-Co₂TiO₄/Ti exhibits a higher apparent Co–O coordination number (model CN ≈ 3) and increased disorder, reflecting stronger local structural perturbation associated with RuO₂ nanocluster formation. Overall, Co K-edge EXAFS, supported by XPS analyses, confirm that Ru incorporation preserves the inverse spinel framework of Co₂TiO₄/Ti, maintaining both Co²⁺ (T_d_) and Co³⁺ (O_h_) coordination environments.

| **Table S4**. *Fitting parameters for the Co–O first-shell path in Ru(SA)-Co_2_TiO_4_/Ti, Ru(NC)–Co₂TiO₄/Ti catalysts, and Ru(SA)-Co_2_TiO_4_/Ti catalyst after CER. Coordination numbers (N) were fixed during EXAFS modeling to improve fit stability: N = 2 for Ru(SA)-Co_2_TiO_4_/Ti before and after CER, and N = 3 for Ru(NC)-Co_2_TiO_4_/Ti. The amplitude factor (amp ≈ S₀²) was fitted and used to approximate effective coordination. ΔR denotes the fitted deviation from the crystallographic Co–O bond length in bulk Co₂TiO₄ (2.01 Å, ICSD #5910130). All fits were performed in R-space with k = 2.4–12.2 Å⁻¹, R = 1.4–3.2 Å, and k-weights 1–3. R values are fitted interatomic distances obtained from artemis/feff modeling (phase-corrected); ft peak positions shown in figures are phase-uncorrected.* | | | | | | | |
| --- | --- | --- | --- | --- | --- | --- | --- |
| Sample | Path | N  fixed | R  Å | ΔR  Å | σ²  Å² | E₀  eV | amp S₀² |
| *Ru(SA)-Co_2_TiO_4_/Ti* | Co–O | 2 | 1.94 | –0.07 | 0.0049 | –0.60 | 0.744 |
| Ru(NC)-Co_2_TiO_4_/Ti | Co–O | 3 | 1.96 | –0.05 | 0.0050 | –0.63 | 0.741 |
| *Ru(SA)-Co_2_TiO_4_/Ti -post CER* | Co–O | 2 | 1.95 | –0.06 | 0.0058 | –0.82 | 0.720 |

**Figure S11**. EXAFS fitting results at the Co K-edge for Co₂TiO₄-based catalysts. (a) R-space (|FT[χ(k)k³]| vs. R) and (b) k-space (χ(k)k³ vs. k) spectra for Co₂TiO₄/Ti, Ru(NC)-Co_2_TiO_4_/Ti, Ru(SA)-Co_2_TiO_4_/Ti, and Ru(SA)-Co_2_TiO_4_/Ti post CER arranged from **bottom to top** in both panels. R-space fitting was performed in the range R = 1.4–3.2 Å and k = 2.4–12 Å⁻¹ using k-weights 1–3 and paths derived from the Co₂TiO₄ CIF (ICSD #5910130). Dashed vertical lines in the R-space indicate approximate positions of the main Co–O (~1.6–2.0 Å) and Co–metal (~2.4 Å) scattering peaks. Only first-shell coordination was modeled for Ru(NC)-Co_2_TiO_4_/Ti, Ru(SA)-Co_2_TiO_4_/Ti, and Ru(SA)-Co_2_TiO_4_/Ti post CER. The plots confirm the retention of Co–O coordination environments across samples.

| Table S5. *Summary of Co K-edge EXAFS Fitting Parameters for Co₂TiO₄-based Catalysts* | | | | | | | | | | |
| --- | --- | --- | --- | --- | --- | --- | --- | --- | --- | --- |
| *Sample* | *R-factor* | *Independent Points* | *Variables* | *k-range (Å⁻¹)* | *R-range (Å)* | *CN (Co–O)* | *CN (Co–Co)* | *CN (Co–Ti)* | *S₀²* | *Notable Features* |
| *Co₂TiO₄/Ti* | *0.0877* | *19.6* | *18* | *2.4–12.2* | *1.4–3.2* | *2.0* | *2.0* | *2.0* | *0.73* | *Reference Co–O, Co–Co, Co–Ti* |
| *Ru(SA)-Co_2_TiO_4_/Ti* | *0.1112* | *10.7* | *6* | *2.5–12.0* | *1.4–3.2* | *3.0* | *3.0* | *3.0* | *0.74* | *Slightly higher CNs* |
| *Ru(SA)-Co_2_TiO_4_/Ti post-CER* | *0.1886* | *11.1* | *6* | *2.5–11.9* | *1.5–3.4* | *2.0* | *2.0* | *2.0* | *0.72* | *Higher R-factor post-CER* |
| *Ru(NC)-Co_2_TiO_4_/Ti* | *0.1400* | *10.6* | *6* | *2.5–12.0* | *1.5–3.3* | *2.0* | *2.0* | *2.0* | *0.93* | *Higher S₀² (denser oxide)* |

**Figure** **3d** presents the k^3^-weighted Ru K-edge FT-EXAFS spectra of the prepared Ru(SA)-Co_2_TiO_4_/Ti and Ru(NC)-Co_2_TiO_4_/Ti catalysts and related reference materials. The FT-EXAFS spectrum for the Ru K-edges shows evidence of scattering paths from the metal ions to neighboring light atoms, such as Ru–O (~1.5 Å , phase-uncorrected), and Ru–Cl (~1.8 Å) contributions, as well as scattering from heavier metal atoms,^[38]^ which provide a qualitative approximation of interatomic distances in the local coordination environment.^[39 ]^

The prominent peak for the Ru–O–Ru scattering feature, which appears at ~3.15 Å in the RuO_2_ spectra, was absent in the Ru(SA)-Co_2_TiO_4_/Ti catalyst, provides evidence of corroborating the atomic dispersion of Ru single atoms. However, the appearance of Ru–O–Ru scattering as sub-peaks in the Ru(NC)-Co_2_TiO_4_/Ti catalyst demonstrates the coexistence of Ru–O–Ru coordination characteristic of RuO₂ nanoclusters. Notably, the Ru–O–Ru peak is much weaker for Ru(NC)-Co_2_TiO_4_/Ti than for RuO_2_, which is most likely due to the small cluster size and lack of structural periodicity.^[40]^

Additionally, RuO₂ exhibits a shoulder at ~2.51 Å (uncorrected for phase shift), corresponding to the Ru–Ru scattering path at ~3.11 Å in the rutile structure. ^[41]^ This feature is not present in Ru(SA)-Co2TiO4/Ti and is strongly attenuated in Ru(NC)-Co2TiO4/Ti, indicating the absence of bulk-like RuO₂ domains in these samples. Instead, a peak at about 2.42 Å appears in both catalysts, suggesting bonding with an element having a shorter radius, most likely Co–Ru or Ti–Ru distances.^[42]^ However, the Ti L-edge spectra of the Ru(SA)-Co_2_TiO_4_/Ti catalyst show no distortions of the original Co_2_TiO_4_/Ti structure upon the introduction of Ru (**Figure S12**). Unlike in Ru(SA)-Co_2_TiO_4_/Ti, the introduction of RuCl_3_ in Ru(NC)-Co_2_TiO_4_/Ti enhances the destruction of the L₃ edge of e_g_ splitting compared with Ru(SA)-Co_2_TiO_4_/Ti and Co_2_TiO_4_/Ti (see spectral circuit in **Figure S13**), indicating irregular distortions of symmetry in various directions and degrees.^[43]^ This finding aligns with the Ru single-atom configuration observed in the XRD and HRTEM results, which show atomically dispersed Ru single atoms incorporated into the Co₂TiO₄ lattice.

To assess the local coordination environments of Ru species, EXAFS fitting was performed using Artemis with FEFF-based paths derived from tetragonal RuO₂ (Materials Project MP-825).**^[38]^** For Ru(NC)-Co_2_TiO_4_/Ti (**Figure S12, Table S6**), multi-shell fitting revealed that Ru atoms adopt distorted octahedral coordination with two short Ru–O bonds at ~1.95 Å and four longer Ru–O bonds at ~2.00 Å, in agreement with bulk RuO₂ rutile-type structure.**^[39]^** The presence of Ru–Ru scattering paths at ~3.13 Å and higher confirms the existence of nanoclusters deposited on the Co₂TiO₄ surface.^[40]^ Conversely, the EXAFS fitting of Ru(SA)-Co_2_TiO_4_/Ti (**Figure S12, Table S6**) yielded only first-shell Ru–O coordination without any observable Ru–Ru paths, indicating atomically dispersed Ru single-atom centers stabilized on the Co₂TiO₄ lattice. The Ru–O bond distances (~1.96 Å) suggest six-fold oxygen coordination in a distorted octahedral environment typical for isolated Ru single atoms with mixed Ru³⁺/Ru⁴⁺ oxidation states, in line with previous studies on atomically dispersed Ru single sites.**^[41]^**

In the Ru(SA)-Co_2_TiO_4_/Ti catalyst after CER, EXAFS fitting yielded a slightly negative Debye–Waller factor for the short Ru–O path. The weak Ru–Ru contribution introduced in the post-CER fitting likely reflects transient, non-periodic Ru–Ru interactions or fitting instability associated with chloride coordination and increased structural disorder, rather than the formation of crystalline RuO₂ clusters. While this may result from limited signal-to-noise or instability in the fit, it may also reflect partial coordination by non-oxygen ligands such as chloride. Notably, a Ru–Cl path (~1.8 Å) appears in the RuCl₃ reference spectrum (see Figure [3d]), supporting this hypothesis. Additional spectroscopic evidence for chloride retention at Ru sites is discussed later based on post-CER XANES and FT-EXAFS features (see Figure 5g and discussion in Section 3.4). Full fitting parameters and statistical details are summarized in **Tables** **S6–S7**.

**Figure S12.** EXAFS fitting results at the Ru K-edge for RuO₂–Co₂TiO₄/Ti catalysts and reference RuO₂. (a) Fourier-transformed k³-weighted EXAFS spectra (FT-EXAFS) in R-space, showing experimental (black) and fitted (red) spectra for Ru(SA)-Co_2_TiO_4_/Ti (fresh and post-CER), Ru(NC)-Co_2_TiO_4_/Ti, and bulk RuO₂, arranged from top to bottom. The absence of Ru–Ru peaks beyond 3 Å in the EDTA-modified samples supports single-atom Ru coordination, while the appearance of Ru–Ru scattering in the Cl-modified sample indicates the formation of nanoclusters. (b) Corresponding EXAFS spectra in k-space, fitted using FEFF-based paths from tetragonal RuO₂ (MP-825) in Artemis (Demeter 0.9.26). Fitting used k-weights of 1, 2, and 3 over the range 3–12 Å⁻¹.

| Table S6. EXAFS fitting parameters for Ru K-edge of reference RuO₂, Ru(SA)-Co_2_TiO_4_/Ti, Ru(NC)-Co_2_TiO_4_/Ti, and Ru(SA)-Co_2_TiO_4_/Ti post-CER data. N is the model coordination number; ΔR is the fitted deviation from the crystallographic path; σ² is the Debye–Waller factor; s₀² is the amplitude reduction factor. Negative σ² values (flagged with *) are physically implausible and may reflect overfitting or noise-related artifacts. | | | | | | | |
| --- | --- | --- | --- | --- | --- | --- | --- |
| Catalyst | Path | N (model) | R (Å) | ΔR (Å) | σ² (Å²) | E₀ (eV) | S₀² |
| RuO₂ reference | Ru–O (short) | 2.0 | 1.94 | 0.01 | 0.00028 | 0.38 | 0.74 |
|  | Ru–O (long) | 4.0 | 1.99 | 0.01 | 0.0035 |  |  |
|  | Ru–Ru | 2.0 | 3.13 | 0.02 | 0.0012 |  |  |
|  | Ru–Ru (2^nd^) | 8.0 | 3.55 | 0.02 | 0.0085 |  |  |
|  | Ru–O–Ru | 8.0 | 3.56 | 0.02 | 0.0061 |  |  |
| Ru(SA)-Co_2_TiO_4_/Ti | Ru–O (short) | 2.0 | 1.97 | 0.033 | 0.0067 | 3.38 | 0.66 |
|  | Ru–O (long) | 4.0 | 2.02 | 0.034 | 0.0005 |  |  |
| Ru(SA)-Co_2_TiO_4_/Ti (post-CER) | Ru–O (short) | 2.0 | 1.98 | 0.041 | –0.0007* | 6.03 | 0.52 |
|  | Ru–O (long) | 4.0 | 2.03 | 0.043 | 0.00152 |  |  |
|  | Ru–Ru | 2.0 | 3.18 | 0.067 | 0.00640 |  |  |
| Ru(NC)-Co_2_TiO_4_/Ti | Ru–O (short) | 2.0 | 1.94 | 0.0096 | 0.00252 | 3.81 | 0.43 |
|  | Ru–O (long) | 4.0 | 1.99 | 0.0101 | 0.00027 |  |  |
|  | Ru–Ru | 2.0 | 3.12 | 0.0172 | 0.00828 |  |  |
|  | Ru–Ru (2^nd^) | 8.0 | 3.54 | 0.0175 | 0.00859 |  |  |
|  | Ru-O-Ru | 8.0 | 3.55 | 0.0175 | 0.00124 |  |  |
| *Negative σ² for Ru-O (short) in Ru(SA)-Co_2_TiO_4_/Ti post-CER may reflect overfitting or altered coordination due to chloride adsorption post-CER. The weak Ru–Ru contribution in the post-cer fit is interpreted as non-periodic/disordered interactions rather than crystalline ruo₂ clustering (see text).  S₀²: amplitude reduction factor accounting for many-body effects. For comparison of coordination numbers, CN_eff_ = S₀² × n may be used. R values are fitted interatomic distances obtained from artemis/feff modeling (phase-corrected); ft peak positions shown in figures are phase-uncorrected. ΔR = R – R_eff_ (relative to path from RuO₂ CIF, material project referenced mp-825). All data fitted in R-space using *Demeter 0.9.26*, k = 3–11.95 Å⁻¹, r = 1.2–4.5 Å (varied slightly between samples). | | | | | | | |

| **Table S7**: Summary of Ru K-edge EXAFS Fitting Parameters Fitting results obtained using Artemis (Demeter 0.9.26), k-weight = 1–3, with scattering paths from tetragonal RuO₂ (MP-825).^[38]^ | | | | | | | | | |
| --- | --- | --- | --- | --- | --- | --- | --- | --- | --- |
| Sampe | k-range Å⁻¹ | R-range Å | S₀² | R-factor | χ² | Reduced χ² | N_indep_ | N_vars_ | Notes |
| Bulk RuO₂ | 2.5–11.9 | 1.3–6.0 | 0.740 | 0.137 | 4616.68 | 429.15 | 27.76 | 17 | Multiple Ru–Ru, Ru–O paths included |
| Ru(SA)-Co_2_TiO_4_/Ti | 3.0–11.95 | 1.25–3.0 | 0.662 | 0.153 | 663.97 | 247.06 | 16.69 | 14 | Only Ru–O paths; no Ru–Ru detected |
| Ru(SA)-Co_2_TiO_4_/Ti  (post-CER) | 3.0–11.95 | 1.25–3.0 | 0.519 | 0.126 | 196.45 | 52.61 | 9.73 | 6 | Ru–O + Ru–Ru₁ added; possible clustering |
| Ru(NC)-Co_2_TiO_4_/Ti | 3.0–11.95 | 1.2–4.5 | 0.430 | 0.131 | 1021.11 | 121.19 | 18.43 | 10 | Ru–O and Ru–Ru₁,₂ included; nanocluster |

**Figure S13.** Ti L-edge XANES spectra of 1) Co_2_TiO_4_/Ti (red), 2) Ru(SA)-Co_2_TiO_4_/Ti (dark yellow), and 3) Ru(NC)-Co_2_TiO_4_/Ti (green).

## Electrochemical investigation

### **Electrocatalytic process**


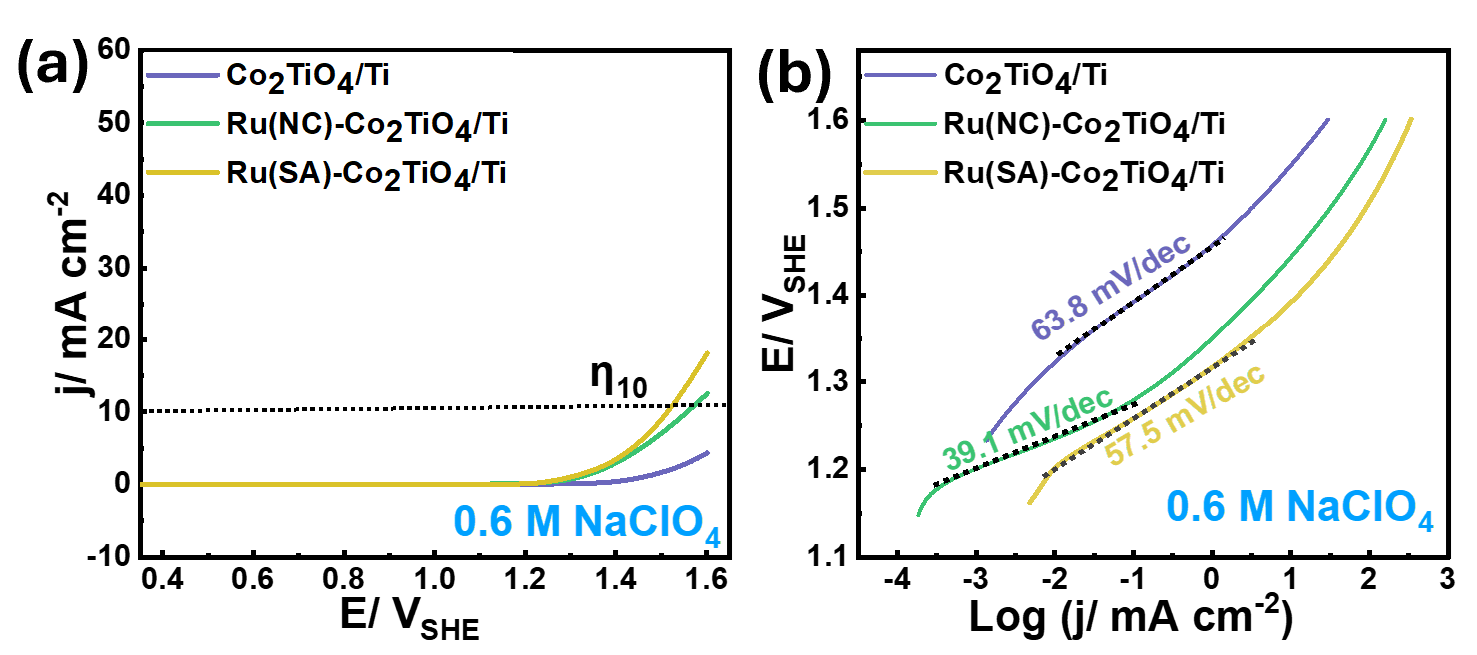


**Figure S14.** Electrocatalytic process using Co_2_TiO_4_/Ti, Ru(NC)-Co_2_TiO_4_/Ti, and Ru(SA)-Co_2_TiO_4_/Ti catalysts with corresponding (a) LSV curves and (b) Tafel slopes in 0.6 M NaClO_4_. The scan rate is 2 mV s^⁻1^.

### **Electrochemical active surface area (ECSA)**


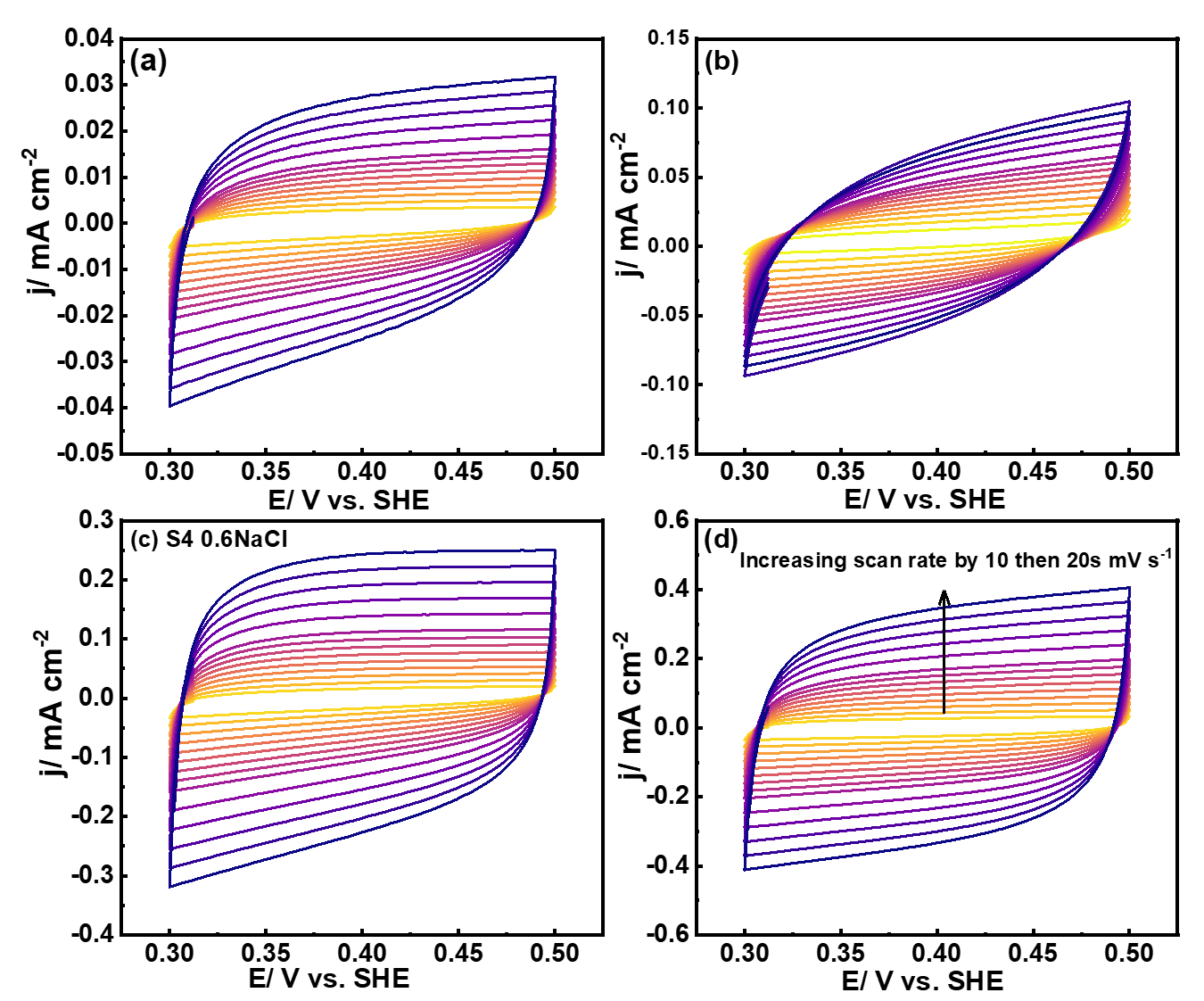


**Figure S15.** Cyclic voltammograms at different scan rates (10-200 mV/s) for (a) TiM, (b) Co_2_TiO_4_/Ti, (c) Ru(NC)-Co_2_TiO_4_/Ti, and (d) Ru(SA)-Co_2_TiO_4_/Ti catalysts in 0.6 M NaCl electrolyte. To ensure the resulting current is non-Faradaic, the CV windows were set from 0.30 to 0.50 V_SHE_. The scan rate increased by 10 mV/s up to 100 mV/s, and then by 20 mV/s to reach 200 mV/s.

### **Electrochemical impedance spectroscopy (EIS)**

The Gerischer impedance was introduced to describe a diffusion-type impedance where species like chlorine and oxygen may participate in first-order chemical reactions along the diffusion path, leading to the formation of higher free chlorine states (Eq. 2-3) ^[42]^. This impedance is distinct from the CPE, which represents a combination of capacitance and resistance. In Gerischer impedance, the interfacial concentration of species involved in homogeneous reactions depends on the forward reaction rate (k_a_), which appears to be the fastest in the Ru(SA)-Co_2_TiO_4_/Ti catalyst (thus, the higher the charge transfer rate, the lower the charge transfer resistance) ^[43]^. Notes that the Gerischer impedance is observed when the interfacial concentration of electroactive species is influenced by mass transport, electronic charge transfer (electrochemical kinetics), and the partial reaction rates of a homogeneous first-order chemical reaction in the diffusion layer, as well as the reversible electrochemical process at an electrode interface.

Cl_2_ + H_2_O→HClO + Cl^−^+ H^+^ Eq. 2

Cl_2_ + 2O_2_ → 2ClO_2_ Eq. 3

O_2_ +4H^+^ +4e^−^ → 2H_2_O Eq. 4


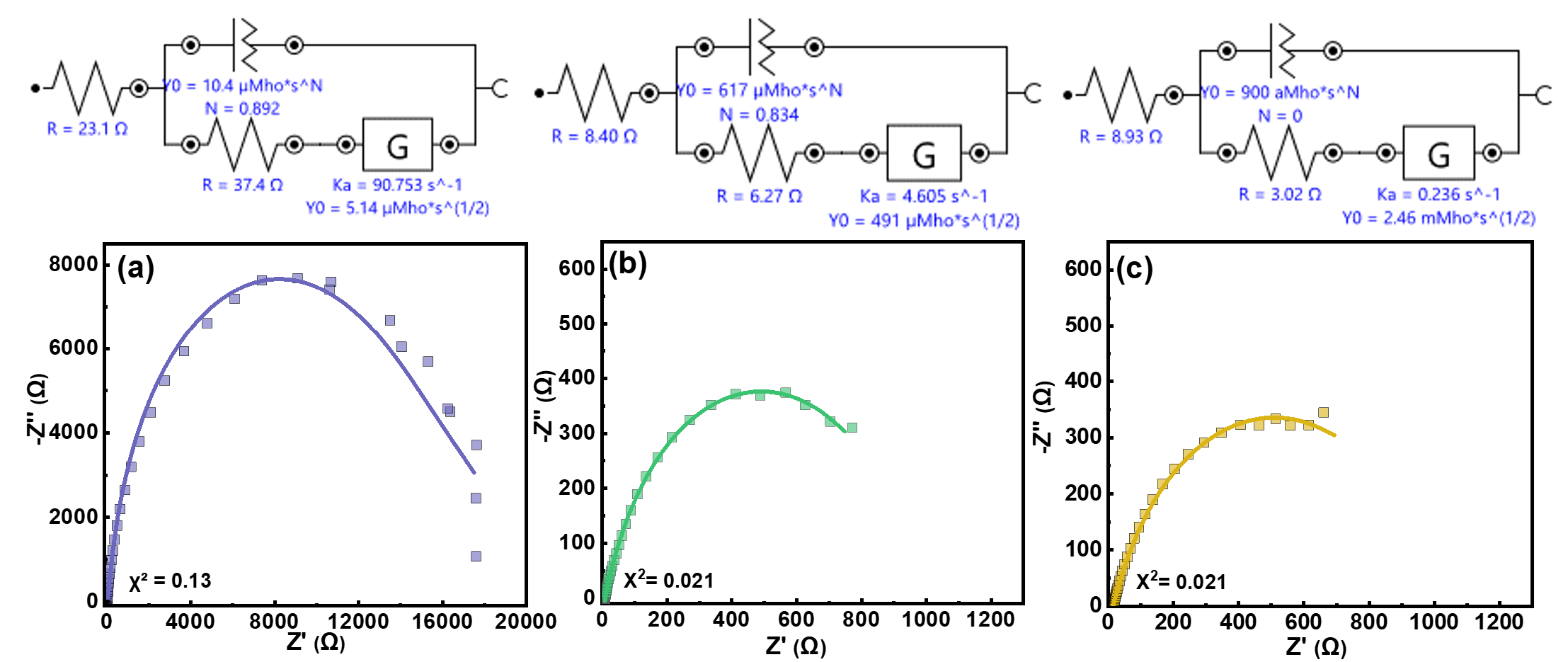


**Figure S16.** Nyquist plots and their inset EIS equivalent fitting circuit model for (a) Co_2_TiO_4_/Ti, (b) Ru(NC)-Co_2_TiO_4_/Ti, and (c) Ru(SA)-Co_2_TiO_4_/Ti catalysts in 0.6M NaCl electrolyte and at exchange potential of 1.23 V_SHE_.

| **Table S8*.*** Comparison of overpotentials obtained at 10 mA cm⁻² reported in recent literature for CER using nanoparticulate, mixed-oxide, and single-atom-based electrocatalysts. | | | | | |
| --- | --- | --- | --- | --- | --- |
| Catalyst | Overpotential  @10 mA cm^-2^ (mV) | Tafel slope  (mV dec^-1^) | Catalyst loading mg cm^-2^ | Conditions | Ref. |
| Ru-O_4_ SAM | 30 | 48.2 | 0.05 | 1 M NaCl, pH 1 | ^[44]^ |
| CS-Ru NPs | 110 | 50.1 | 0.05 | 1 M NaCl, pH 1 | ^[44]^ |
| DSA | 85 | 48.9 | -- | 1 M NaCl, pH 1 | ^[44]^ |
| DSA | 115 | 53.2 | -- | 1 M NaCl, pH 1 | ^[45]^ |
| Ir-OM-AC | 243 | 92 | 2.1% (Ir based) | 0.1 M HClO_4_+3.5 wt% NaCl, pH 1 | ^[46]^ |
| HPC-0.05 | 94 | 131.7 | -- | 4 M NaCl, PH 2 | ^[47]^ |
| RuO_2_ NPs@TiO_2_ | 70 | 39 | -- | Saturated NaCl, pH 3 | ^[48]^ |
| TNA-RuO_2_ | 100 | -- | -- | 0.1 M NaCl, pH 6 | ^[49]^ |
| IrO_2_-Ta_2_O_5_/TiO_2_ | -- | 52 | 0.7 | 4 M NaCl, pH 1 | ^[50]^ |
| Ru-S-TiO_2_ | 70 | 67 | -- | 0.2 M NaCl, pH 1 | ^[51]^ |
| RuOx/2D TiOx | 98 | 43.6 | 0.255 | 1 M NaCl, pH 1 | ^[45]^ |
| 2D TiOx | 339 | -- | 0.255 | 1 M NaCl, pH 1 | ^[45]^ |
| RuO_2_ | 125 | 53.2 | 0.255 | 1 M NaCl, pH 1 | ^[45]^ |
| Ru-Ti-IrO_x_/Ti | 125.2 | -- | -- | 4 M NaCl, pH 2 | ^[52]^ |
| Co_3_O_4_ NPs | 90 | 44 | 1 | 0.6 M NaCl, pH Neutral | ^[53]^ |
| Ti/RuO_2_–IrO_2_- Sb_2_O_5_–SnO_2_ | 75 | 36.3 | 2 | Seawater | ^[54]^ |
| Boron-Doped Diamond | > 142 | 145 | -- | 4.0 M NaCl, pH 1 | ^[55]^ |
| Co₂TiO₄/Ti | 126.4 | 44.5 | 2.42 | 0.6 M NaCl, pH 4.5 | This work |
| Ru(NC)-Co_2_TiO_4_/Ti | 49.5 | 40.5 | 0.19 | 0.6 M NaCl pH 4.5 | This work |
| Ru(SA)-Co_2_TiO_4_/Ti | 26.2 | 39.2 | 0.08 | 0.6 M NaCl pH 4.5 | This work |
| SAM: single atom moiety, NP: nano particles, DSA: dimentionaly stable anode, Ir-OM-AC: Ir organometallic-based electrocatalysts, HPC-0.05: hollow porous carbon nanocages, TNA: TiO_2_ nanotube array, 2D: two dimensional, | | | | | |

| Sample | C | N | O | Co | Ti | Ru | Cl |
| --- | --- | --- | --- | --- | --- | --- | --- |
| Post CER Ru(SA)-Co_2_TiO_4_/Ti | 53.6 | 0.4 | 32.8 | 4.0 | 4.5 | 3.78 | 0.85 |
| Fresh Ru(SA)-Co_2_TiO_4_/Ti | 48.2 | 0.9 | 38.9 | 5.4 | 3.1 | 3.41 | - |

**Figure S17.** XPS survey scan of Ru(SA)-Co_2_TiO_4_/Ti catalyst post 0.6M NaCl electrolysis at 20 mA cm^-2^ for 72h. The table indicates a comparison of elemental composition of the catalyst prior to and after CER determined using XPS survey scan (at%)


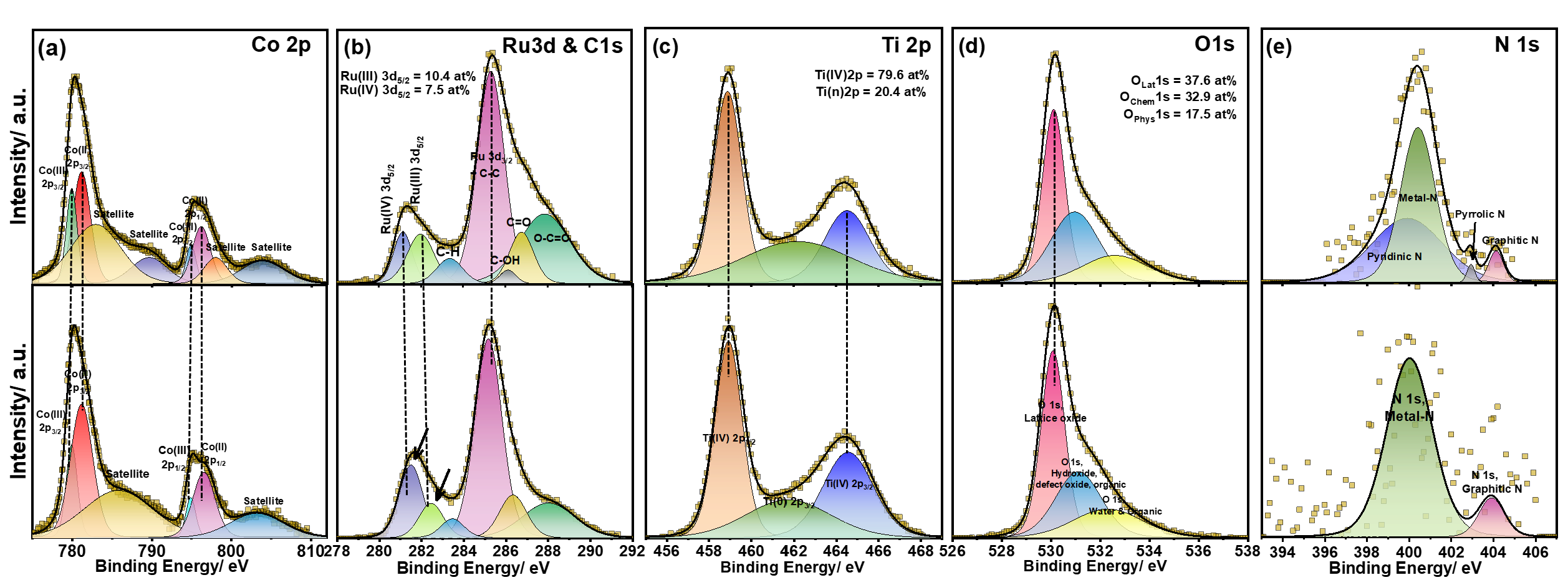


**Figure S18.** The HR-XPS of (a) Co2p, (b) Ru3d and C1s, (c) Ti2p, (d) O1s, and (e) N1s regions, aligned of lower to upper panel: Ru(SA)-Co_2_TiO_4_/Ti catalyst for the freshly prepared catalyst aligned in the top panel to compare with the same catalyst post 0.6M NaCl electrolysis at 20 mA cm^-2^ for 72h.

**
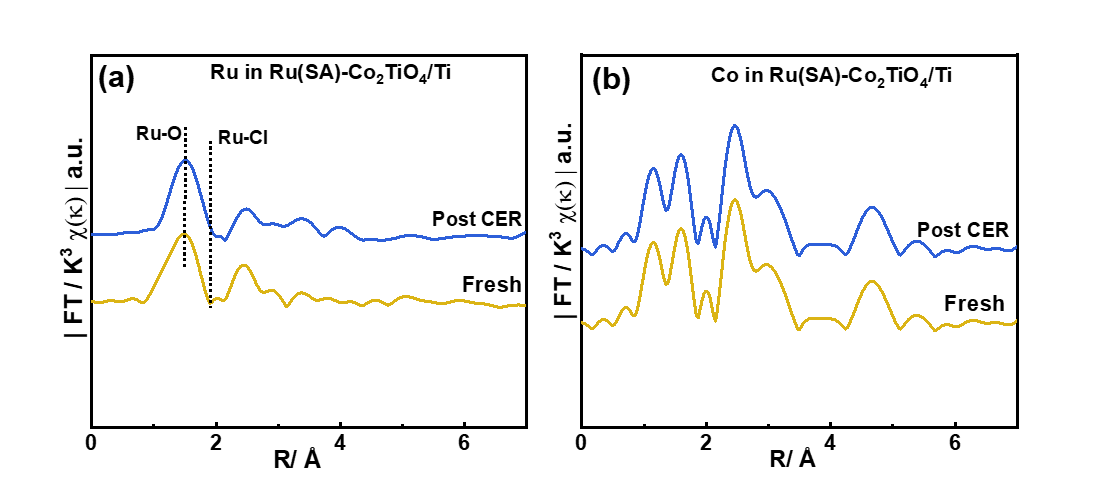
**

**Figure S19.** FT-EXAFS spectra of using Ru(SA)-Co_2_TiO_4_/Ti catalyst in (a) Ru K-edge and (b) Co K-edge regions before and after 72h electrocatalysis in 0.6M NaCl.

**Electrochemical Insights into the CER Mechanism**

Considering both the Tafel slopes (**Table 1**) and [Cl⁻]-dependent steady state potential, a first-order electrochemical CER with respect to [Cl⁻] concentration can be inferred for both Ru(SA)-Co_2_TiO_4_/Ti and Ru(NC)-Co_2_TiO_4_/Ti catalysts.

$\eta_{act}=-\frac{RT}{\propto F}\text{ln}\frac{j_{0}}{j}$ Eq. S5

$j=j_{0}\exp\left( -\frac{\propto F\eta_{act}}{RT} \right)$ Eq. S6

$b=\frac{\partial E}{\partial\log j}=\frac{2.303 RT}{\propto F}$ Eq. S7

$\left( \frac{\partial E}{\partial\log[{Cl}^{-}]} \right)_{j}=-\left( \frac{\partial Log j}{\partial\log[{Cl}^{-}]} \right)_{E}\left( \frac{\partial E}{\partial\log j} \right)_{[{Cl}^{-}]}$ Eq. S8

where $\eta_{act}$ the anodic part of the activation overpotential, *α* is the charge transfer coefficient,*z*is the number of electron transferred,*i_0_*is the exchange current density (A m^−2^), *i* is the current density (A m^−2^),*R*is the ideal gas constant,*T*is the absolute temperature (K), and*F*is the Faraday constant (C mol^−1^).

# References

[1] W. H. R. Shaw, J. J. Bordeaux, *J. Am. Chem. Soc.* **1955**, 77, 4729.

[2] W.-J. Liu, L. Dang, Z. Xu, H.-Q. Yu, S. Jin, G. W. Huber, *ACS Catal.* **2018**, 8, 5533.

[3] D. Fang, F. He, J. Xie, L. Xue, *Journal of Wuhan University of Technology-Mater. Sci. Ed.* **2020**, 35, 711.

[4] CasaXPS. Peak Fitting in XPS., <https://www.casaxps.com/help_manual/manual_updates/peak_fitting_in_xps.pdf>, accessed: January 5, 2025.

[5] T. Regier, J. Krochak, T. K. Sham, Y. F. Hu, J. Thompson, R. I. R. Blyth, *Nuclear Instruments and Methods in Physics Research Section A: Accelerators, Spectrometers, Detectors and Associated Equipment* **2007**, 582, 93.

[6] a) B. K. Teo, *EXAFS: basic principles and data analysis*, Springer Science & Business Media, **2012**; b) B. Ravel, & Newville, M., *J. Synchrotron Radiation* **2005**, 12, 537; c) A. Jain, S. P. Ong, G. Hautier, W. Chen, W. D. Richards, S. Dacek, S. Cholia, D. Gunter, D. Skinner, G. Ceder, K. A. Persson, *APL Mater.* **2013**, 1.

[7] Hach Company. Method 8021—Free Chlorine, DPD Method., <https://www.hach.com> accessed: January 5, 2025.

[8] E. Faulques, D. L. Perry, S. Lott, J. D. Zubkowski, E. J. Valente, *Spectrochimica Acta Part A* **1998**, 54, 869.

[9] K. Krishnan, R. A. Plane, *J. Am. Chem. Soc.* **1968**, 90, 3195.

[10] M. Jafari, M. Vaezzadeh, S. Noroozizadeh, *Metallurgical and Materials Transactions A* **2010**, 41, 3287.

[11] S. K. Jasmin Vijitha, K. Mohanraj, R. P. Jebin, *Chem. Phys. Impact* **2023**, 6, 100183.

[12] K. Feng, H. Zhang, J. Gao, J. Xu, Y. Dong, Z. Kang, J. Zhong, *Appl. Phys. Lett.* **2020**, 116.

[13] a) A. Kim, C. Sanchez, G. Patriarche, O. Ersen, S. Moldovan, A. Wisnet, C. Sassoye, D. P. Debecker, *Catal. Sci. Technol.* **2016**, 6, 8117; b) L. Howard-Fabretto, T. J. Gorey, G. Li, S. Tesana, G. F. Metha, S. L. Anderson, G. G. Andersson, *Nanoscale Adv.* **2021**, 3, 3537.

[14] T. R. Gengenbach, G. H. Major, M. R. Linford, C. D. Easton, *J. Vacuum Sci. Technol. A* **2021**, 39.

[15] C. L. Bianchi, V. Ragaini, M. G. Cattania, *Mater. Chem. Phys.* **1991**, 29, 297.

[16] a) S. Maria, I. Theophilos, in *Cobalt*ISBN (Ed: M. Khan), IntechOpen, Rijeka **2017**, 978-953-51-3668-2; p. Ch. 3; b) P. W. Menezes, A. Indra, D. González-Flores, N. R. Sahraie, I. Zaharieva, M. Schwarze, P. Strasser, H. Dau, M. Driess, *ACS Catal.* **2015**, 5, 2017; c) Y. Lykhach, S. Piccinin, T. Skála, M. Bertram, N. Tsud, O. Brummel, M. Farnesi Camellone, K. Beranová, A. Neitzel, S. Fabris, K. C. Prince, V. Matolín, J. Libuda, *J. Phys. Chem. Lett.* **2019**, 10, 6129.

[17] a) S. J. Gerber, E. Erasmus, *Mater. Chem. Phys.* **2018**, 203, 73; b) M. C. Biesinger, B. P. Payne, A. P. Grosvenor, L. W. M. Lau, A. R. Gerson, R. S. C. Smart, *Appl. Surf. Sci.* **2011**, 257, 2717.

[18] a) L. Ma, C. Y. Seo, X. Chen, K. Sun, J. W. Schwank, *Appl. Catal. B* **2018**, 222, 44; b) Y. Luo, Y. Zheng, J. Zuo, X. Feng, X. Wang, T. Zhang, K. Zhang, L. Jiang, *J. Hazard. Mater.* **2018**, 349, 119.

[19] a) F. Besenbacher, J. K. Nørskov, *Progress in Surf. Sci.* **1993**, 44, 5; b) L. Lukashuk, N. Yigit, R. Rameshan, E. Kolar, D. Teschner, M. Hävecker, A. Knop-Gericke, R. Schlögl, K. Föttinger, G. Rupprechter, *ACS Catal.* **2018**, 8, 8630.

[20] Z. Wang, R. Lin, Y. Huo, H. Li, L. Wang, *Adv. Funct. Mater.* **2022**, 32, 2109503.

[21] a) T. Marshall-Roth, N. J. Libretto, A. T. Wrobel, K. J. Anderton, M. L. Pegis, N. D. Ricke, T. V. Voorhis, J. T. Miller, Y. Surendranath, *Nat. Commun.* **2020**, 11, 5283; b) Y. Cai, J. Fu, Y. Zhou, Y.-C. Chang, Q. Min, J.-J. Zhu, Y. Lin, W. Zhu, *Nat. Commun.* **2021**, 12, 586.

[22] L. Wang, T. Qi, J. Wang, S. Zhang, H. Xiao, Y. Ma, *J. Hazard. Mater.* **2018**, 342, 579.

[23] Y. Xia, Y. Jiang, F. Li, M. Xia, B. Xue, Y. Li, *Appl. Surf. Sci.* **2014**, 289, 306.

[24] a) T. N. Pham, D. Shi, T. Sooknoi, D. E. Resasco, *J. Catal.* **2012**, 295, 169; b) J. Ying, J.-B. Chen, Y.-X. Xiao, S. I. Cordoba de Torresi, K. I. Ozoemena, X.-Y. Yang, *J. Mater. Chem. A* **2023**, 11, 1634; c) J. Lim, D. Park, S. S. Jeon, C.-W. Roh, J. Choi, D. Yoon, M. Park, H. Jung, H. Lee, *Adv. Funct. Mater.* **2018**, 28, 1704796.

[25] D. J. Morgan, **2015**, 47, 1072.

[26] W. I. Choi, S. Choi, M. Balamurugan, S. Park, K. H. Cho, H. Seo, H. Ha, K. T. Nam, *ACS Omega* **2023**, 8, 35034.

[27] a) J. Ying, J.-B. Chen, Y.-X. Xiao, S. I. Cordoba de Torresi, K. I. Ozoemena, X.-Y. Yang, *J. Mater. Chem. A* **2023**, 11, 1634; b) K. Wang, Y. Wang, B. Yang, Z. Li, X. Qin, Q. Zhang, L. Lei, M. Qiu, G. Wu, Y. Hou, *Energy & Environmental Science* **2022**, 15, 2356.

[28] K. Wang, Y. Wang, B. Yang, Z. Li, X. Qin, Q. Zhang, L. Lei, M. Qiu, G. Wu, Y. Hou, *Energy Environ. Sci.* **2022**, 15, 2356.

[29] P. Kumar, K. Kannimuthu, A. S. Zeraati, S. Roy, X. Wang, X. Wang, S. Samanta, K. A. Miller, M. Molina, D. Trivedi, J. Abed, M. A. Campos Mata, H. Al-Mahayni, J. Baltrusaitis, G. Shimizu, Y. A. Wu, A. Seifitokaldani, E. H. Sargent, P. M. Ajayan, J. Hu, M. G. Kibria, *J. Am. Chem. Soc.* **2023**, 145, 8052.

[30] M. Uchikoshi, K. Shinoda, *Structural Chem.* **2019**, 30, 945.

[31] a) K. Getty, M. U. Delgado-Jaime, P. Kennepohl, *Inorganica Chimica Acta* **2008**, 361, 1059; b) W. Ziyu, N. L. Saini, S. Agrestini, D. D. Castro, A. Bianconi, A. Marcelli, M. Battisti, D. Gozzi, G. Balducci, *J. Physics: Cond. Matter* **2000**, 12, 6971.

[32] a) K. Jiang, M. Luo, Z. Liu, M. Peng, D. Chen, Y.-R. Lu, T.-S. Chan, F. M. F. de Groot, Y. Tan, *Nat. Commun.* **2021**, 12, 1687; b) X. Liang, S. Yang, J. Yang, W. Sun, X. Li, B. Ma, J. Huang, J. Zhang, L. Duan, Y. Ding, *Appl. Catal. B* **2021**, 291, 120070.

[33] D. Chatterjee, *New J. Chem.* **2020**, 44, 18972.

[34] a) B. Ravel, & Newville, M., *Journal of Synchrotron Radiation* **2005**, 12, 537; b) J. J. Rehr, R. C. Albers, *Reviews of Modern Physics* **2000**, 72, 621.

[35] H.-Y. Wang, S.-F. Hung, Y.-Y. Hsu, L. Zhang, J. Miao, T.-S. Chan, Q. Xiong, B. Liu, *J. Phys. Chem. Lett.* **2016**, 7, 4847.

[36] C. O. D. (COD), Co₂TiO₄ structure, COD ID: 5910130, <https://www.crystallography.net/cod/5910130.html>, accessed: June 18, 2025, ISBN.

[37] a) Z. Liu, G. Wang, X. Zhu, Y. Wang, Y. Zou, S. Zang, S. Wang, *Angew. Chem.* **2020**, 59, 4736; b) S. Thota, M. Reehuis, A. Maljuk, A. Hoser, J. U. Hoffmann, B. Weise, A. Waske, M. Krautz, D. C. Joshi, S. Nayak, S. Ghosh, P. Suresh, K. Dasari, S. Wurmehl, O. Prokhnenko, B. Büchner, *Phys. Rev. B* **2017**, 96, 144104; c) J. Chen, H. Li, Z. Yu, C. Liu, Z. Yuan, C. Wang, S. Zhao, G. Henkelman, S. Li, L. Wei, Y. Chen, *Adv. Energy Mater.* **2020**, 10, 2002593.

[38] M. Project, Materials Data on RuO_2_ (mp-825), <https://materialsproject.org/materials/mp-825/>, accessed: June 28, 2025, ISBN.

[39] a) B. B. Sarma, F. Maurer, D. E. Doronkin, J.-D. Grunwaldt, *Chem. Rev.* **2023**, 123, 379; b) J. Du, Y. Huang, Z. Huang, G. Wu, B. Wu, X. Han, C. Chen, X. Zheng, P. Cui, Y. Wu, J. Jiang, X. Hong, *JACS Au* **2022**, 2, 1078.

[40] A. I. Frenkel, *Chem. Soc. Rev.* **2012**, 41, 8163.

[41] S. Zuo, Z.-P. Wu, D. Xu, R. Ahmad, L. Zheng, J. Zhang, L. Zhao, W. Huang, H. Al Qahtani, Y. Han, L. Cavallo, H. Zhang, *Nat. Commun.* **2024**, 15, 9514.

[42] A.-K. Meland, D. Bedeaux, S. Kjelstrup, *J. Phys. Chem. B* **2005**, 109, 21380.

[43] M. S. Harding, B. Tribollet, V. Vivier, M. E. Orazem, *J. Electrochem. Soc.* **2017**, 164, E3418.

[44] Y. Liu, C. Li, C. Tan, Z. Pei, T. Yang, S. Zhang, Q. Huang, Y. Wang, Z. Zhou, X. Liao, J. Dong, H. Tan, W. Yan, H. Yin, Z.-Q. Liu, J. Huang, S. Zhao, *Nat. Commun.* **2023**, 14, 2475.

[45] J. Ji, J. Liu, L. Shi, S. Guo, N. Cheng, P. Liu, Y. Gu, H. Yin, H. Zhang, H. Zhao, *Small Struct.* **2024**, 5, 2300240.

[46] J. S. Ko, J. K. Johnson, P. I. Johnson, Z. Xia, *ChemCatChem* **2020**, 12, 4526.

[47] H. Qu, B. Li, Y. Ma, Z. Xiao, Z. Lv, Z. Li, W. Li, L. Wang, **2023**, 35, 2301359.

[48] J. Huang, M. Hou, J. Wang, X. Teng, Y. Niu, M. Xu, Z. Chen, *Electrochim. Acta* **2020**, 339, 135878.

[49] J. Kim, C. Kim, S. Kim, J. Yoon, *J. Ind. Eng. Chem.* **2018**, 66, 478.

[50] L. Deng, Y. Liu, G. Zhao, J. Chen, S. He, Y. Zhu, B. Chai, Z. Ren, *J. Electroanal. Chem.* **2019**, 832, 459.

[51] A. R. Jadhav, X. Liu, P. Silambarasan, V. Kanade, Y. Liu, T. T. T. Nga, T. Yang, M. T. Kim, Y. Han, T. Kim, X. Shao, C. Zhi, C.-L. Dong, H. Lee, *Appl. Catal. B* **2024**, 359, 124456.

[52] A. R. Zeradjanin, N. Menzel, W. Schuhmann, P. Strasser, *PCCP* **2014**, 16, 13741.

[53] H. Ha, K. Jin, S. Park, K.-G. Lee, K. H. Cho, H. Seo, H.-Y. Ahn, Y. H. Lee, K. T. Nam, *J. Phys. Chem. Lett.* **2019**, 10, 1226.

[54] S. Wang, H. Xu, P. Yao, X. Chen, *Electrochemistry (ECSJ)* **2012**, 80, 507.

[55] S. Ferro, A. De Battisti, I. Duo, C. Comninellis, W. Haenni, A. Perret, *J. Electrochem. Soc.* **2000**, 147, 2614.
